# On the validity versus utility of activity landscapes: are all activity cliffs statistically significant?

**DOI:** 10.1186/1758-2946-6-11

**Published:** 2014-04-02

**Authors:** Rajarshi Guha, José L Medina-Franco

**Affiliations:** 1 NIH Center for Advancing Translational Science, 9800 Medical Center Drive, Rockville, MD 20850, USA; 2 Circuito Exterior, Instituto de Química, Universidad Nacional Autónoma de México, Ciudad Universitaria, México D.F. 04510, Mexico; 3 Current address: Mayo Clinic, 13400 East Shea Boulevard, Scottsdale, AZ 85259, USA

**Keywords:** Consensus models, Structure-activity relationships, Significant activity cliffs, Structure Activity Landscape Index

## Abstract

**Background:**

Most work on the topic of activity landscapes has focused on their quantitative description and visual representation, with the aim of aiding navigation of SAR. Recent developments have addressed applications such as quantifying the proportion of activity cliffs, investigating the predictive abilities of activity landscape methods and so on. However, all these publications have worked under the assumption that the activity landscape models are “real” (i.e., statistically significant).

**Results:**

The current study addresses for the first time, in a quantitative manner, the significance of a landscape or individual cliffs in the landscape. In particular, we question whether the activity landscape derived from observed (experimental) activity data is different from a randomly generated landscape. To address this we used the SALI measure with six different data sets tested against one or more molecular targets. We also assessed the significance of the landscapes for single and multiple representations.

**Conclusions:**

We find that non-random landscapes are data set and molecular representation dependent. For the data sets and representations used in this work, our results suggest that not all representations lead to non-random landscapes. This indicates that not all molecular representations should be used to a) interpret the SAR and b) combined to generate consensus models. Our results suggest that significance testing of activity landscape models and in particular, activity cliffs, is key, prior to the use of such models.

## Background

The notion of activity landscapes has been extensively discussed since it was first coined by Maggiora [1]. While such landscapes can be defined in many ways, they are represented fundamentally as two structural dimensions that aim to capture as much as possible the information of the structures in chemical space, plus one activity dimension. This allows for intuitive visual depictions and in particular this view of landscapes is useful when discussing activity cliffs–discontinuities in the landscape that correspond to molecules that are very similar but differ significantly in activity. Most workers have devised alternative numerical characterizations of landscapes including Structure-Activity Similarity (SAS) and related maps [2,3], the Structure Activity Landscape Index (SALI) [4] and the Structure Activity Relationship Index (SARI) [5]. On the basis of these definitions many applications have been described. For example, Guha and Van Drie described the use of SALI curves to characterize the ability of a predictive model to characterize the landscape and correctly predict activity cliffs [6,7] and the use of SALI to enable model selection [8]. Bajorath and co-workers have published a series of papers that identify and characterize different types of SAR’s in datasets [9,10]. They also employ landscapes in the context of target and target family specificity [11,12], which serve to highlight the fact that activity cliffs are dependent on the target [13] This is further supported by the lack of SAR transfer among different target families [14]). Medina-Franco and co-workers have addressed the topic of consensus activity landscapes, which characterize landscapes using multiple structural representations [15]. In general, it is clear that activity cliffs are very dependent on the context, which can be defined in multiple ways–by target, by chemical series or by molecular representation.

A key aspect of the landscape paradigm is that molecular representation can significantly alter the structure of the landscape. As pointed out by Shanmugasundaram et al. [16] molecules that are similar in one representation can be dissimilar in another. As a result, activity cliffs, which in various forms a primary focus of most activity landscape analyses, are generally representation dependent. Some approaches such as consensus approaches [17] allow one to alleviate this problem by identifying pairs of molecules that are activity cliffs in multiple representations (suggesting that they are “true” activity cliffs). Although one cannot eliminate activity cliffs that are formed by only one or few representations, for practical applications one can prioritize the SAR of the consensus activity cliffs [18].

In addition to a structural representation dependence of activity cliffs, there is also a dependence on activity. Past work has generally assumed that the observed activity together with the structure actually encodes a structure-activity relationship. For example, in traditional QSAR modeling, a key test of any model is its performance when faced with a permuted dependent variable (also known as y-scrambling) [19]. However, we are not aware of any work that has addressed the *statistical significance* of a landscape or individual cliffs in the landscape. While Guha et al. identified significant cliffs, [8,20] this was simply based on a threshold (usually the 90th or 95th quantile of the observed SALI values) and did not actually address whether a cliff was a true cliff or an artificial cliff due to the molecular representation used or erroneous measures of activity [21]. In general, there has been little done to address whether landscapes, however they are defined, are significant in terms of being different from a random landscape.

### Validity and utility

In absence of measures of significance, most approaches to activity landscapes have simply assumed that they are *valid*, and then have gone on to explore the *utility* of the landscape for various purposes. For example, it is assumed that reported activity data is correct and there are no attempts to assess if the associated landscapes are not different from random. In this context, a valid landscape implies that an SAR is present. In this paper, we propose that methods that employ the landscape paradigm should first perform checks to assess the validity of the landscape. More specifically, is the landscape being employed different from one that could be generated by chance? Assuming that the landscape does pass such a test and is thus valid, one can then continue to assess the utility of the landscape for the task at hand. The concept of comparing predictive models to models developed using random (or scrambled) data is common when developing and validating quantitative models such as QSAR equations [19]. However, using randomization methods have not been tested when investigating activity landscapes and their applications.

In this study we also consider the validity of individual activity cliffs. Thus, having considered the significance of a landscape, we then go on to assess the significance of individual activity cliffs. While it is possible to envision several ways to address this issue (depending on the activity landscape method), in this work we specifically employ the SALI formulation of activity cliffs and combine the idea of threshold-based identification of SALI cliffs [4,8] with a permutation test to identify significant activity cliffs–significant in the sense of magnitude as well as statistics. To test the hypothesis whether a landscape and its activity cliffs are valid, we used several data sets and different molecular representations previously used to generate activity landscapes.

## Results

### Significance testing for landscapes

**Figure 1:**
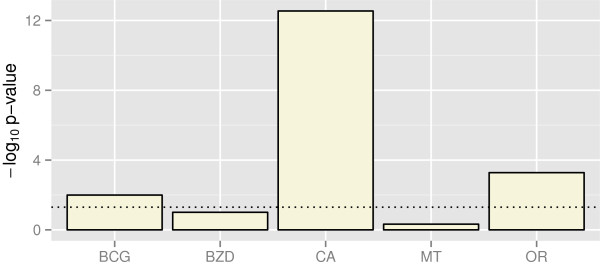
**A comparison of activity landscape significance for the datasets studied in this work, using MACCS fingerprints for structure representation.** The dotted line represents p = 0.05.

We evaluated the significance of entire landscapes for each endpoint/structure representation combination listed in Table 1. We first summarized individual datasets using the MACCS representations and a single target (when activity data for more than one target was available). Figure 1 presents the results of the KS test, where the negative log_10_ of the p-values are plotted so that taller bars indicate greater significance. The plot only shows the results obtained using the MACCS representation. It is evident that not all datasets lead to a landscape that is different from random (in a statistically significant sense). Similar results are observed if we consider other representations. Our data also indicate that there is no representation that consistently leads to non-random landscapes. There was no obvious correlation between representations (and thus chemical structure) and the significance of the landscape. One might expect that artifactual datasets (such as ones with artificially flat or jagged landscapes) would allow one to draw general conclusions and indeed such artificially generated datasets tend to be consistently insignificant. But in practice, it appears that such *a priori* conclusions cannot be made.

**Table 1:** Summary of datasets employed in this study

**Dataset**	**Num. Obs.**	**Targets**	**Endpoints**	**Representations**	**Ref.**
BZD	91	Benzimidazole analogues tested against *G. intestinallis* (Gi)	pIC_50_	MACCS, piDAPH3, TGD	[22-25]
BCG	48	Bicyclic guanidines with Kappa opioid receptor binding affinity	pIC_50_	MACCS, piDAPH3, TGD	[15]
OP	98	Delta, Kappa, Mu opioid receptors	pK_i_	MACCS, Atompairs, Radial, piDAPH3	[26,27]
CA	96	CA I, CA II, CA IX, CA XII	pK_i_	MACCS, Atompairs, Radial, piDAPH3	[26,27]
MT	299	Norepinephrine, serotonin and dopamine transporters	Potency (nM)	MACCS, Atompairs, Radial, piDAPH3	[26,27]

**Figure 2:**
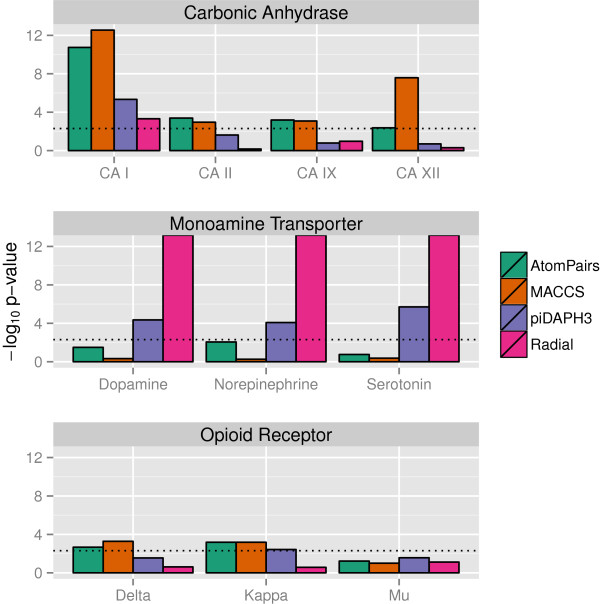
**A summary of the landscape significance test for three target families.** The dotted line corresponds to p = 0.05 and so bars above this line correspond to landscapes that are different from random (in a statistically significant sense).

We then considered landscapes for the same sets of molecules but against multiple target families, viz., the opioid receptor, carbonic anhydrase isoforms and monoamine transporters. In all cases, the same set of representations was employed and the results of the KS test are summarized in Figure 2. Once again it is clear that the results are dataset dependent. Interestingly, it appears that the radial descriptor representation tends to lead to non-significant landscapes, with only three datasets (CA I, CA IX and delta Opioid) exhibiting a non-random landscape with this descriptor. This observation suggests that the landscape significance of a given dataset is not obviously correlated to the complexity of the structure representation used in the calculation. To conduct a comprehensive assessment of different fingerprints and their significance on activity landscapes, large scale studies will be required comparing several different fingerprints across diverse target families.

In terms of cross-target analysis, Figure 2 suggests that the structural representation should be carefully selected, such that it leads to valid landscapes for all targets that are to be compared. For example, in the case of the opioid receptor datasets, there is no representation that leads to a statistically significant landscape across all three targets. For the case of the CA datasets, it is only the atom pair and MACCS representations that lead to statistically non-random landscapes over all four targets. Of course, these conclusions apply for a given level of significance. Finally for the MT datasets, we see that the radial and piDAPH3 representations lead to non-random landscapes, with p-values approaching zero, across all targets.

### Significance testing for individual SALI values

**Figure 3:**
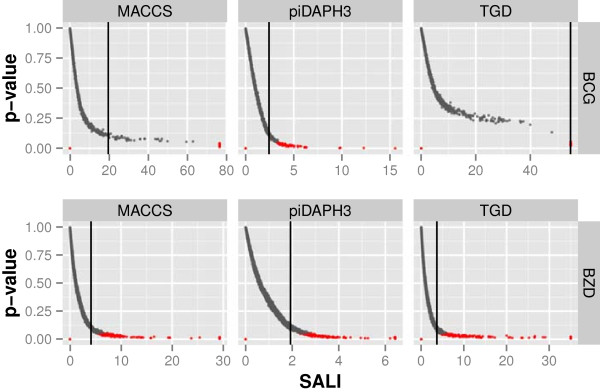
**Summary of per-pair SALI significance calculations for the BCG and BZD datasets using different representations.** Points in red correspond to pairs with a SALI whose empirical p-value < 0.05. The vertical line corresponds to the 95th percentile of the SALI values for that dataset.

We next consider the significance of individual SALI values for the BCG and BZD datasets using MACCS, TGD and piDAPH3, summarized in Figure 3. In each panel, the red points correspond to pairs of molecules for which the SALI value is calculated to be no different from random (at the 0.05 level). The vertical line is drawn at the 95th percentile of the SALI values for that dataset and representation. Note that from Table 2, all representations for these datasets do not lead to non-random landscapes at the 0.05 level of significance. However, even so, it is clear that SALI values for some pairs of compounds are deemed to be significant. At this point, we do not consider the per-SALI significance values for landscapes that are identified as non-significant.

**Table 2:** A summary of KS test p-values for the landscape significance test for the BCG and BZD datasets using multiple representations

**Dataset**	**Representation**	**p-value**	**F**_**magnitude**_	**F**_**significant**_
BCG	MACCS	0.010	0.063	0.063
	piDAPH3	0.307	0.050	NA
	TGD	< 2.2E-16	0.214	0.211
BZD	MACCS	0.098	0.050	NA
	piDAPH3	0.063	0.050	NA
	TGD	0..018	0.050	0.049

F_magnitude_ represent the fraction of SALI values that are identified as significant cliffs based on their magnitude being greater than a cutoff (95th percentile of the SALI values). F_significant_ is similar but only considers SALI values that are greater than the cutoff and exhibit a p-value < 0.05. If the landscape was not deemed significant, the latter was not reported.

If we consider the piDAPH3 representation for the BZD dataset it is clear that in the absence of the permutation test, all SALI values to the right of the vertical line would be considered “significant”, simply in terms of magnitude. However, on the basis of the p-values and a pre-defined level of significance, only a subset of those points are different from random SALI values. For each SALI value that is identified as being significant (196 for the case of the BZD / piDAPH3 combination), the 95% region of the random SALI population does not contain them. Thus an alternative way to define the test of significance is to state that a SALI value is significant if it is not contained between the 5th and 95th quantiles of the random SALI population (for that pair of molecules). Table 2 reports the fraction of SALI values that are identified as significant in terms of magnitude only (F_magnitude_), and the fraction that are significant in terms of magnitude as well as p-value (F_significant_). Since we assume that non-significant landscapes should not be studied, we report F_significant_ = NA for those cases.

### Effects of combining representations

The preceding analyses have focused on activity landscapes generated from single structural representations. Given the fact that cliffs in one representation may not be cliffs in another, consensus approaches have been described [17], that employ multiple representations to evaluate the landscape. There are two approaches that one can take in this scenario. First, one identifies compound pairs that are deemed activity cliffs in the landscapes derived from the individual representations. Second, one can generate an aggregate representation by combining the multiple individual representations [3]; In the first approach, one focuses the SAR analysis on pairs of compounds that are identified by most (or all if possible) representations. In the second case, one analyses the activity landscape derived from the aggregate representation.

**Figure 4:**
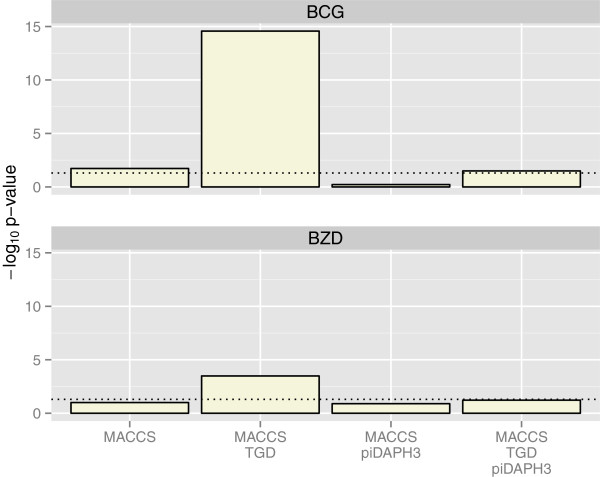
**The results of the landscape significance test for the BCG and BZD datasets using a single representation and various combinations of representations.** The horizontal line is drawn at p = 0.05.

To investigate the effect of aggregate representations we consider the BCG and BZD datasets and various combinations of fingerprint representations. To combine the data we computed the mean similarity (“mean fusion”) of different representations. This approach, based on the principles of data fusion, has been largely used to generate not only consensus models of the activity landscape but also in many documented applications of data fusion [28-30]. Figure 4 summarizes the landscape significance test for the MACCS representation and three combination representations for the BCG and BZD. For the BCG dataset it appears that increasing the number of representations in the fusion does not necessarily lead to significant landscapes. For the BCG dataset, two representations (MACCS + TGD) lead to a non-random landscape, but switching to MACCS + piDAPH3 does not. For both datasets, the three-representation case is identified as non-random. Similar behavior is seen in the BZD dataset. Given that it is not clear as to how one might *a priori* judge whether a given combination of representations will be significant (and the possible number of combinations could easily become too large to examine explicitly), consensus landscapes (and cliffs) are probably best analyzed using multiple, individual representations and the landscapes derived from them.

## Discussion

What are the implications of using random data in SAR studies using activity landscape approaches? What does it mean for a landscape to be random? One interpretation of such landscapes that emerge from the current approach is that they are “artifacts” and the structural representation does not reliably encode a real SAR; in other words, since similar landscapes can be generated from randomized activity and structural data there is no true connection between the structural features captured by the representation and the activity data. The analysis presented in this work has shown that for a given data set, there are structural representations that generate activity landscapes that are not different from random. It follows that conclusions obtained from non-significant landscapes are spurious. This interpretation is in line with the interpretation of QSAR models that fail the y-randomization test [19]. In contrast, non-random landscapes suggest that there is a real association between the observed activity and the chemical space defined by the structural representation(s).

Our analyses suggest that there is no clear *a priori* feature of a dataset and its representation that would suggest that it is not different from random. Instead, one must perform a permutation test such as one described here to determine the validity or non-validity of the landscape. It should be noted that landscapes identified as non-random by the approach described here may not necessarily encode an SAR. An example of such a case would be a flat landscape, in which the activity of all molecules is (near) constant. While such a landscape is certainly not random, it does not represent a useful SAR. However, simply examining the activity and descriptor data readily identifies such anomalous landscapes. In such cases one would probably not consider them for further analyses.

We have approached significance by comparing observed SALI distribution to random distribution of activity data. Alternative would be to fit a distribution to the observed SALI values. But this is difficult since observed SALI distributions are rarely normal. One might consider the distributions of activity differences and dissimilarity separately, but in general, automated distribution fitting will be difficult. Importantly, the random SALI distribution is derived from a scrambled dependent variable. We also considered a modification of the approach whereby we sampled the dependent variable in each randomization step from a normal distribution with the same mean and variance as the observed dependent variable. While the results were somewhat different from those reported here, we believe that this approach is not appropriate, as the observed dependent variable is usually a mixture of a normal and long tailed distribution, due to the presence of activity cliffs. Thus sampling from a single normal distribution is unlikely to generate activity cliffs during the simulation.

While the aim of the proposed significance test is to justify the use of a landscape for further study, the test could also be used to prospectively identify suitable descriptors for use in a landscape analysis. It is still debatable on what is “the best” structure representation to analyze activity landscapes. This issue is more pronounced when developing consensus models (e.g. combining representation with low linear correlation and/or representations with different design [3]). But in either case, it makes sense that at the very least the landscape should be different from random. The fact that one can perform the permutation test on single or combined representations allows one to rationally select representations for landscape analysis. Indeed, earlier work has suggested the use of SALI as part of a model selection procedure^8^. While this ensures that the selected model captures the landscape, it is clear that significance testing should be incorporated to ensure that the model captures a *valid* landscape. In a similar vein, significance testing for individual SALI values (across different representations) is a systematic way to identify statistical significant activity cliffs that should be prioritized for SAR analysis/interpretation. Following the rationale of consensus models of activity landscapes, one can define *consensus significant activity cliffs* as cliffs deemed significant by a number of different representations.

## Conclusions

In this work we have presented an approach to address the important but currently overlooked question of whether the SAR captured in a model of activity landscape constructed with a given molecular representation(s) is different from random. Using the SALI formalism to quantify the activity landscape and its activity cliffs, we have shown that some structure representations lead to activity landscapes that are indistinguishable from landscapes generated using random activity data and randomized similarity values. For the current study that involved six different data sets with activity against one or more molecular targets, and different sets of 2D and 3D fingerprints, we concluded that statistically significant landscapes are dataset and representation dependent (in addition to activity cliffs themselves being target and chemical series dependent). Testing the statistical significance of molecular representations is a rational approach to select molecular representations to generate robust activity landscape models and identify statistically significant activity cliffs. Of course, if observed activity data were available in replicate, one could more directly evaluate whether a landscape or cliff were different from noise. In absence of such data, we believe that the permutation tests described here is an appropriate test of the choice of representation employed in landscape activity analyses. While we have focused on the use of SALI, the proposed method is applicable to other metrics of the landscape that include an activity and similarity term in their formulation (such as SARI or SAS maps) and we hope that future studies involving activity landscapes will employ this (or some other) form of significance testing before asking questions of such models.

## Methods

### Valid vs. non-valid landscapes

The proposed approach is inspired by y-scrambling. Given a set of structures and their observed activity values, we evaluate the observed SALI matrix, defined by

$${\text{SALI}}_{i,j}=\frac{\left|A_{i}-A_{j}\right|}{1-\mathrm{sim}\left(i,j\right)}$$

where *A*_*i*_ and *A*_*j*_ are the observed activities for molecules *i* and *j*, and *sim*(*i*, *j*) is the similarity between the two molecules (measured with a given molecular representation and similarity metric). In contrast to y-scrambling for QSAR models, the dependent variable in the SALI formalism is a function of the activity and similarity. As a result, scrambling the dependent variable requires that one scramble both activity and similarity. In addition, as has been well documented, similarity measures depend critically on the molecular representations and similarity coefficients used [31,32]. Thus, in addition to the uncertainties in the activities, there are also uncertainties in the similarity values. But since similarities are not statistically independent, permuting the similarities directly is not possible. Instead we shuffle the fingerprints and then use the shuffled set to evaluate scrambled similarities. We then scrambled the activities and using the scrambled similarity matrix recalculated the SALI matrix, repeating this N times. Each such SALI matrix thus represents a “random” landscape. While scrambling the activity variable is a well accepted approach in permutation tests (e.g., in QSAR models) there are number of ways one could consider randomization of the similarity values. The approach described here (i.e., shuffle the ordering of the fingerprints, as opposed to scrambling the bits in the fingerprints themselves) is rather simple to implement. But more generally, one would replace scrambled similarities with a component that considered the significance of the similarity value. Given that there is no well accepted and generalized approach to the calculation of the significance of a chemical similarity, the development of such a method is beyond the scope of the current work and so we focus on activity and similarity scrambling as the primary driver of random landscapes. The N SALI matrices are then converted to 1D vectors with *m*(*m* - 1)/2 elements where *m* is the number of molecules in the dataset. The vectors are then concatenated, resulting in a single vector of length *Nm*(*m* - 1)/2 elements.

It is important to note that multiple scramblings are required to build up the distribution of random SALI values. Comparison of the observed SALI values to SALI values computed from a single randomization would not allow a robust differentiation between observed and random values (i.e., a single iteration could appear to be very similar to the observed SALI values, though highly unlikely). Note that if it were possible to analytically define the distribution of SALI values, such an empirical process would not be required. However, based on examination of the current datasets the distribution suggests some form of a combination of normal and exponential distributions. It is unclear exactly how one might define such a mixture of distributions automatically for any dataset.

The final step is to compare the distributions of the original (observed) SALI values and the ones calculated from the N scrambled activities. We employ the Kolmogorov-Smirnov (KS) test [33] to perform this comparison, in which the null hypothesis is that the two distributions are the same. We select *α* such that if the p-value from the KS test is less than *α* we can reject the null hypothesis. In such a scenario we would claim that the observed landscape is different from random and that the difference is statistically significant. We term such a landscape as valid.

We also investigated whether the individual random SALI matrices were distinguishable or indistinguishable from the accumulated distribution using the KS test. As expected, this was dataset dependent. Considering the MACCS fingerprints, the BCG dataset led to 1000 random SALI matrices being characterized as different from the accumulated distribution of SALI values. For the BZD dataset fingerprints, 103 out of 1000 random matrices were identified as different and for the MT (norepinephrine) dataset 727 were identified as different. Importantly, the random matrices identified as being different from the accumulated distribution would not be considered valid, simply because they were constructed to be random.

### Significance of activity cliffs

Assuming a landscape is valid, we can then consider individual pairs of molecules and assess whether they represent statistically significant activity cliffs. For this purpose we first defined a threshold SALI value such that pairs of molecules exhibiting a SALI greater than the threshold were deemed “significant” (in terms of magnitude) activity cliffs. For the selected pairs we then counted the number of times the random SALI value (described above) for that pair was greater than the observed value, denoted by *N*_*greater*_. The ratio *N*_*greater*_/*N* is taken as the empirical p-value and if less than *α*, the pair is deemed to be a statistically significant cliff. (Due to the scrambling procedure one can consider an arbitrary cell in the randomized SALI matrices or even all cells across all the randomized SALI matrices (in which case the denominator would become *Nm*^2^) for the purposes of this calculation. For simplicity of calculation we consider randomized values for the pair in question).

For the purposes of this study we selected the SALI threshold to be the 90th quantile of the observed SALI values for a given dataset. We also set *N* = 1000 and *α* = 0.05. All calculations were performed in R 2.15.0 on a MacBook Pro (OS X 10.8.2, 2.7GHz Intel Core i7 with 16GB of RAM). The multicore package was employed to parallelize calculations. All code is available as Additional file 1.

### Datasets

We considered six datasets, previously used to generate activity landscape models with sizes ranging from 47 to 300 compounds. In some cases, the structures had been tested against multiple targets and thus multiple activity values were available. The structures were characterized by multiple fingerprint schemes frequently used in activity landscape models including MACCS keys, radial fingerprints and pharmacophore fingerprints, available in the Molecular Operating Environment [15] and Canvas [34]. Table 1 summarizes the details of the datasets.

MACCS keys are 166 bit binary fingerprints, where each bit position corresponds to a specific substructure. GpiDAPH3 is a 3-point pharmacophore based fingerprint computed from the two-dimensional graph. piDAPH4 is a 4-point pharmacophore based fingerprint computed from a three-dimensional conformation. Atompair fingerprints are differentiated by the type and distance separating pairs of atoms. Radial fingerprints entail growing a set of fragments radially from each heavy atom over a series of iterations. Similarity matrices for observed activities were taken from the previous published work summarized in Table 1. As discussed in these publications, the different fingerprint representations typically have different ranges of similarity values.

## Competing interests

The authors declare that they have no competing interests

## Authors’ contributions

JMF conceived of the study. RG and JMF participated in the design and implementation of the calculations as well as drafting the manuscript. Both authors read and approved the final manuscript.

## Supplementary Material

Additional file 1 **Code and data to reproduce figures.** The ZIP file contains the R code and datasets required to perform the calculations and generate the figures for this article. It assumes you have R installed and are working on a Unix-like system. In addition you should have the ggplot2, grid, multicore and fingerprint R packages installed. To run the scripts execute run.sh from the command line. Click here for file
