# Supplementary material for: On the validity versus utility of activity landscapes: are all activity cliffs statistically significant?
Source: J Cheminform. 2014 Apr 2;6:11. doi: 10.1186/1758-2946-6-11 (PMC4021161; doi:10.1186/1758-2946-6-11)
Supplement: Additional file 1 — Code and data to reproduce figures. The ZIP file contains the R code and datasets required to perform the calculations and generate the figures for this article. It assumes you have R installed and are working on a Unix-like system. In addition you should have the ggplot2, grid, multicore and fingerprint R packages installed. To run the scripts execute run.sh from the command line. [file 1758-2946-6-11-S1.zip › si/bajorath/2012_BMC_Activity Similarity-Waddell.pdf]

Bioorganic & Medicinal Chemistry 20 (2012) 5443–5452

Contents lists available at SciVerse ScienceDirect

Bioorganic & Medicinal Chemistry

journal homepage: www.elsevier.com/locate/bmc

Bioactivity landscape modeling: Chemoinformatic characterization of structure–activity relationships of compounds tested across multiple targets

Jacob Waddell, José L. Medina-Franco *

Torrey Pines Institute for Molecular Studies, 11350 SW Village Parkway, Port St. Lucie, FL 34987, USA

ARTICLE INFO

Article history:
Available online 1 December 2011
This work is dedicated to Nicolás Medina Sandoval on the occasion of his 80th birthday.

Keywords:
2D/3D structure representations
Consensus activity cliffs
Molecular similarity
SAS maps
Scaffold hoping
Structure-landscape index

ABSTRACT

Characterizing structure–activity relationships (SAR) of sets of compounds screened across different targets is crucial in several drug discovery endeavors. To this end, chemoinformatic approaches are emerging to characterize SARs using the concept of multi-target activity landscapes. Herein, we present the Structure multiple Activity Similarity (SmAS) maps and the Structure multiple Activity Landscape Index (SmALI) as general approaches to navigate through and quantify the most informative regions of multi-target activity landscapes. These methods are extensions of SAS maps and SALI metric used for single targets. To illustrate the use of these methods, SmAS maps and SmALI values were employed for characterizing the SAR of three benchmark sets of compounds screened with different target families. As a follow up of our work, we employed four 2D and 3D structure representations to obtain consensus models for each data set. For the three data sets, we identified pairs of compounds with high structure similarity but very different bioactivity profile across the corresponding targets of each family that is, multi-target activity cliffs. Also, we identified pairs of compounds with low structure similarity but similar bioactivity profile across the different targets that is, multi-target scaffold hops. The consensus SmAS maps and mean SmALI metric are complementary chemoinformatic tools to systematically describe multi-target activity landscapes.

© 2011 Elsevier Ltd. All rights reserved.

1. Introduction

The description of the structure–activity relationships (SAR) of a set of compounds with measured biological activity is of major interest in medicinal chemistry and drug design. Systematic descriptions of the SAR of compound data sets using the emerging concept of *activity landscape* are designed to access, visualize, and to help understand the data generated from general screening or optimization campaigns. In addition, understanding the underlying basis of the SAR may represent a critical step before applying predictive approaches such as quantitative structure–activity relationships (QSAR). The excellent editorial by Maggiora highlights the importance of the early detection of activity cliffs (chemical compounds with highly similar structures but significantly different biological activities) in QSAR modeling.[1] For example, *apparent* outliers in the data may reflect the presence of activity cliffs and may not be due to statistical fluctuations or to measurement errors.[1,2]

There are an increasing number of methods to model the activity landscape for single targets. These methods are reviewed extensively in Bajorath et al.[3] Approaches include Structure–Activity Relationship Index (SARI),[4] Structure–Activity Landscape Index (SALI),[5,6] network-like similarity graphs (NSG),[7] and Structure–Activity Similarity (SAS) maps.[8] Our group has employed SAS and SAS-like maps to characterize the SAR of different compound data sets for one target.[9–13] Some of the current approaches to model activity landscapes of single targets have been recently adapted to explore the SAR of data sets with biological activity across different biological endpoints[10,14–16] being the research group of Bajorath et al. pioneer of the concepts of multi-target activity landscape modeling and multi-target activity cliffs.[14,15]

Multi-target activity landscape modeling is attractive to characterize the SAR of compound data sets associated with selectivity[15] or promiscuity. For example, 'scaffold-based promiscuity' of the targets.[17] As pointed out by Dimova et al. modeling multi-target activity landscapes, however, is not an easy task because of the challenge to combine potency relationships for several targets.[16] A graph representation[16] and a novel approach using self-organizing maps[18] were recently proposed to visually represent and characterize multi-target activity landscapes. Dual and triple activity-difference (DAD/TAD) maps were also recently introduced to characterize the SAR of compound data sets screened against two or three targets.[11,13] However, DAD and TAD maps are not easily applied to model landscapes for more than three targets.

* Corresponding author. Tel.: +1 772 345 4685; fax: +1 772 345 3649.
E-mail address: jmedina@tpims.org (J.L. Medina-Franco).

0968-0896/$ - see front matter © 2011 Elsevier Ltd. All rights reserved.
doi:10.1016/j.bmc.2011.11.051

Herein, we present an intuitive approach to represent multi-target activity landscapes using the principles of SAS maps. The pairwise SAR of three benchmark data sets used previously to model multi-target activity landscapes[13,16] is explored in this work. For each pair of compounds in the different data sets, the computed molecular similarity was compared with the *bioactivity profile similarity* across multiple targets that is, multi-target activity similarity. Pairwise structure-multi-target activity relationships were visually depicted in 2D plots called in this work Structure multiple Activity Similarity (SmAS) maps. SmAS maps readily identify multi-target activity cliffs, scaffold hops and *smooth* regions in the landscape (regions with pair of compounds with highly similar structures and similar bioactivity profile across multiple targets). Following up on our work, we employed multiple structure representations to generate *consensus* models of the activity landscapes and thus *reduce* the dependence of activity landscape on chemical representation.[19–21] As pointed out previously, consensus models are designed to prioritize the SAR analysis of activity cliffs and other consistent regions in the activity landscape that are captured by several structure representations. Consensus data points are not meant to eliminate data, disregarding for example, 'true' activity cliffs that are not identified by some structure representations.[12,21] Multi-target activity cliffs and scaffold hops were quantified expanding the application of the recently proposed mean SALI measure to multiple targets. We want to emphasize that methods described in this work are not restricted to the data sets employed, the structure representations, similarity functions, or molecular targets. The focus in this work is on the consensus SmAS maps and mean SmALI for the comprehensive modeling of multi-target activity landscape of compounds tested across different targets using multiple structure representations.

2. Methods

2.1. Data set

We employed three data sets tested against different target families recently used in the work of Dimova et al.;[16,22] 299 compounds tested across three monoamine transporters, MT (norepinephrine, serotonine and dopamine transporters); 98 compounds tested across four opioid receptors, OR (delta, kappa, mu, and nociceptin receptor) and 96 compounds tested across four carbonic anhydrases, CA (I, II, IX, and XII). Each molecule in the data set has K_i values reported against each target. The distribution of activities for each target is summarized in Table S1 (Supplementary data). The three data sets were employed previously to model multi-target activity landscapes with NSG[16] and were used as benchmark sets in this work. In the previous study the activity profile was encoded classifying the compounds in three categories.[16] In contrast, in this work we used the potency values with no previous encoding. We also employed a consensus fingerprint representation as opposed to a single representation used in the previous analysis.[16]

2.2. Multiple activity similarity (mAS)

The potency for each molecule (reported in nM units) was expressed in log units using the equation:

$$V_{i,k} = -\log\left[\frac{A_{i,k}}{1.0 \times 10^9}\right] \qquad (1)$$

where $V_{i,k}$ is the potency in log units of the kth target for the ith molecule, and $A_{i,k}$ is the activity value in nM units.

The pairwise activity similarities across p targets that is, multiple activity similarity, were calculated using the Tanimoto coefficient for real-valued vectors:[23]

$$mAS(i,j) = \frac{\sum_{k=1}^{p} v_{i,k} \cdot v_{j,k}}{\sum_{k=1}^{p} v_{i,k}^2 + \sum_{k=1}^{p} v_{j,k}^2 - \sum_{k=1}^{p} v_{i,k} \cdot v_{j,k}} \qquad (2)$$

where $mAS(i,j)$ is the multiple activity similarity of the ith and jth molecules, p is the number of targets, and $V_{i,k}$ and $V_{j,k}$ denote the value of the potency calculated with Eq. 1 of the kth target for the ith and jth molecules, respectively. In this work, $p = 3$ for the compounds tested against the three monoamine transporters and $p = 4$ for the compounds tested against the opioid receptors and carbonic anhydrases, respectively. It is worth nothing that the multiple activity similarity captures with a single measure the similarity of the bioactivity profile of each pair of molecules across all targets. Of note, the multiple activity similarity does not provide information for the change in activity for each target and does not distinguish for example, single-, dual-, triple-target activity cliffs or scaffold hops (vide infra). However, quantitative methods to describe these types of cliffs and scaffold hops have been proposed by the authors and other groups.[13,16]

2.3. 2D and 3D fingerprint representations and structure similarity

A total of four 2D and 3D fingerprints were computed, namely radial (2D) fingerprints (equivalent to the extended connectivity fingerprints, ECFPs)[24] and atom pairs (2D) implemented in Canvas,[25] MACCS keys (2D) (166 bits) and three-point pharmacophores (3D) (piDAPH3) implemented in Molecular Operating Environment (MOE).[26] The distribution of the 44,551, 4753 and 4560 pairwise similarities of the 299 monoamine transporter inhibitors, 98 opioid receptor antagonists, and 96 carbonic anhydrase inhibitors calculated with the four molecular representations are summarized in Table S2 (Supplementary data), respectively. It is worth nothing that each data set has different intra-molecular diversity for example, the set of monoamine transporter inhibitors is more diverse than the opioid receptor antagonists as indicated by all used 2D and 3D fingerprints (Table S2). Despite the inherent issues of employing a single conformer to represent 3D structures, the 3D fingerprints are valuable to characterizing activity landscapes.[9,10,12] To simplify the analysis, a single low-energy conformation of each molecule was used.[9,27] Several conformations can also be considered as the authors recently reported.[12] Structure similarities were computed with the Tanimoto coefficient,[28] which has been successfully applied in a number of cases of activity landscape modeling. For example refer to the several cases reviewed in Wassermann et al.[29] and Wawer et al.[30] and recent applications by

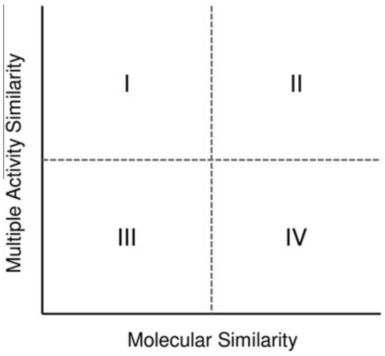

Figure 1. Prototype Structure multiple Activity Similarity (SmAS) map. The plot can be roughly divided in four major regions; I and II contain pairs of compounds for scaffold hopping and smooth SAR, respectively. Region IV contains multi-target activity cliffs. See text for details.

Table 1
Distribution of the pairwise multiple activity similarity (mAS) values for each data set

Set	Max	Q3[a]	Median	Q1[b]	Min	U95[c]	Mean	L95[c]	STD
MT	1	0.973	0.941	0.847	0.350	0.881	0.880	0.879	0.140
OR	1	0.991	0.981	0.958	0.776	0.970	0.969	0.968	0.032
CA	1	0.982	0.953	0.904	0.661	0.936	0.935	0.933	0.061

[a] Third quartile.
[b] First quartile.
[c] 95% Confidence of the mean Upper (U95) and Lower (L95) limits.

our and other groups.[9–13,16] However, other measures such as Euclidean distance can be used.

2.4. Multi-target activity landscapes with consensus SmAS maps

For each pair of compounds the multiple activity similarity was plotted against their structural similarity generating the SmAS maps which are an extension of the SAS maps proposed for single targets.[8] The major difference is that SmAS maps include the bioactivity profile information of each pair of compounds across several targets. A prototype SmAS map is depicted in Figure 1 where the molecular and multiple activity similarities are represented on the *X*- and *Y*-axes, respectively. Four major regions can be *roughly* distinguished in the SmAS maps. Pairs of compounds that fall in region I have low structural similarity, but high multiple activity similarity. These pairs are indicative of multi-target scaffold or side chain (R-group) hopping.[31,32] Region II denotes pairs of compounds with both high structure and high multiple activity similarity and represents compounds in smooth or continuous SARs for all targets. Compounds in region IV have high structure similarity, but low multiple activity similarity that is, very different bioactivity profile across all targets and therefore correspond to multi-target activity cliffs or discontinuous SARs. This quadrant is a region of high SAR information content[23,33] because it reveals structural pat-

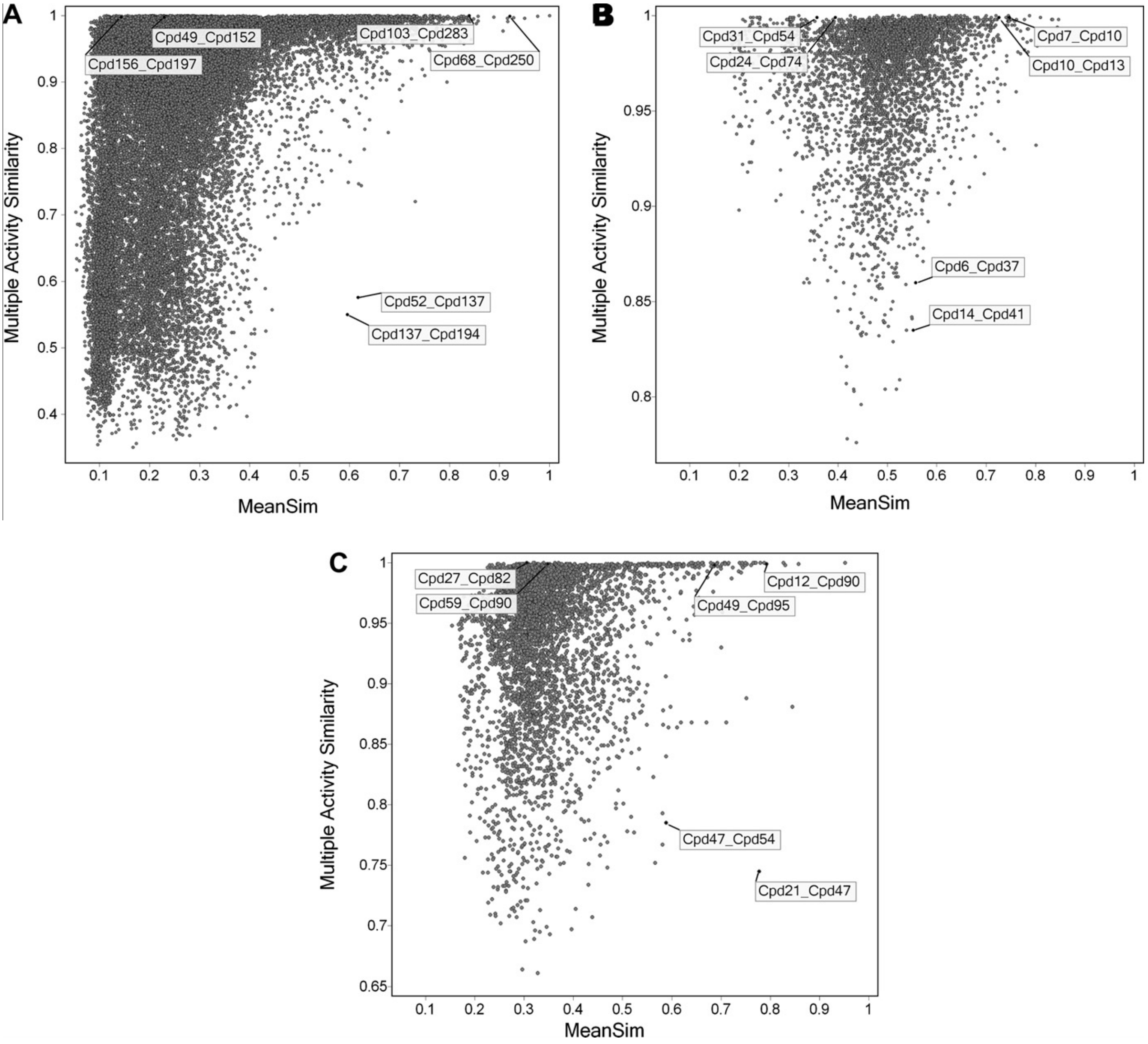

Figure 2. Consensus SmAS maps for (A) 299 monoamine transporter inhibitors showing 44,551 pairwise comparisons; (B) 98 opioid receptor antagonists depicting 4753 pairwise comparisons and (C) 96 carbonic anhydrase inhibitors showing 4560 pairwise comparisons. Selected activity cliffs, scaffold hops and pairs of compounds in the smooth region of the activity landscapes are labeled with the compound numbers. The values of multiple activity similarity and structure similarity are summarized in Tables 2–4, respectively. See text for details.

terns that are crucial for activity for all targets. Region III is the least interesting containing pairs of molecules with low molecular similarity and low multiple activity similarity.

Any similarity measure can be employed in a SmAS map. Following a strategy employed before for single targets, consensus SmAS maps can be obtained when several structure representations that capture different aspects of the chemical structures are considered.[10,12] This strategy has been used to *reduce* the well-known dependence of the activity landscape with structure representation.[9–12,21] One approach to reduce such dependence is identifying activity cliffs common to a series of structure representations, that is, consensus activity cliffs.[9] An alternative strategy is combining similarity measures obtained by different methods using the principles of data fusion,[34–36] for example by computing the mean similarity ('mean fusion') of selected representations.[11,12] Using the second approach, consensus SmAS maps for each of the three targets were obtained in this work by plotting the corresponding multiple activity similarities against the mean structure similarity of radial, atom pairs, MACCS, and piDAPH3 fingerprints (the distribution of the mean similarities for each data set is summarized in Table S2 in the Supplementary data). These fingerprints were used because they showed relatively low linear correlations for the pairwise structure similarities for each data set (for each data set, Table S3 in the Supplementary data show the correlation matrices of the Pearson's correlation coefficient between all the pairwise similarities for each pair of 2D and 3D representations). Also, and more importantly, these fingerprints were employed because they have conceptually different designs capturing dissimilar aspects of the chemical structures. For example, MACCS keys used in this work are a pre-defined set of 166 structural keys; radial fingerprints entail growing a set of fragments radially from each heavy atom over a series of iterations;[24,37] piDAPH3 are spatial three-point pharmacophores employing the following atom types: in pi system, is donor, is acceptor.

2.5. Activity landscapes with Structure multiple Activity Landscape Index (SmALI)

For each data set, the presence of multiple-target activity cliffs was measured by extending the SALI measure proposed by Guha and Van Drie[5,6] to multiple targets using the expression:

$$\mathrm{SmALI}_{i,j} = \frac{1 - mAS_{i,j}}{1 - \mathrm{sim}(i,j)} \quad (3)$$

where $\mathrm{SmALI}_{i,j}$ is the Structure multiple Activity Landscape Index, $mAS_{i,j}$ is the multiple activity similarity of the ith and jth molecules calculated with Eq. 2, and sim(i,j) is the similarity coefficient between the two molecules. Any similarity method can be used to compute SmALI. Mean SmALI values were computed in this work using the mean structure similarity of four 2D and 3D molecular representations (radial, atom pairs, MACCS, and piDAPH3 fingerprints, vide supra).[9,13] As will be shown later, the mean SALI values were consistent in interpreting the SAR of the data set highlighting the feasibility of using mean fusion similarity values in characterizing activity landscapes.

3. Results and discussion

3.1. Consensus SmAS maps

The distribution of the pairwise multiple activity similarity values for each data set, calculated with Eq. 2 described in the Methods is summarized in Table 1. In general, for the three target families, compounds show similar bioactivity profile across the corresponding three or four targets as indicated by the high median (⩾0.941), mean (⩾0.880) of the multiple activity similarities. This is not unexpected since the targets that belong to each target family are, of course, related. Overall, the monoamine transporter

Table 2
Representative consensus multi-target activity cliffs identified in the SmAS maps

Pair	ΔpK_i T1[a]	ΔpK_i T2	ΔpK_i T3	ΔpK_i T4	mAS[b]	Radial	Atom pairs	MACCS	piDAPH3	Mean sim[c]
Monoamine transporter inhibitors (MT)										
**137_194**	4.69	4.65	3.51	NA[d]	0.550 (1.113)	0.35	0.58	0.80	0.65	0.60
**52_137**	−4.52	−4.10	−3.92	NA	0.576 (1.107)	0.36	0.63	0.80	0.67	0.62
**91_137**	3.71	4.03	2.17	NA	0.720 (1.043)	0.37	0.79	0.90	0.87	0.73
**136_137**	−4.09	−2.79	−2.61	NA	0.749 (0.623)	0.35	0.58	0.80	0.66	0.60
Opioid receptor antagonists (OR)										
**14_41**	−2.10	−1.11	−3.33	−3.28	0.835 (0.369)	0.11	0.56	0.85	0.69	0.55
**6_37**	−1.88	−2.74	−2.60	−2.54	0.860 (0.316)	0.08	0.50	0.98	0.67	0.56
**6_36**	−2.15	−1.57	−3.10	−3.46	0.841 (0.353)	0.06	0.55	0.94	0.66	0.55
**48_75**	1.47	2.49	3.65	3.36	0.835 (0.358)	0.11	0.43	0.90	0.71	0.54
Carbonic anhydrase inhibitors (CA)										
**21_47**	−4.76	−2.76	−3.39	2.87	0.745 (1.146)	1.00	0.64	0.82	0.65	0.78
**21_72**	−4.59	−2.98	−3.11	2.44	0.767 (0.556)	0.26	0.32	0.89	0.86	0.58
**47_54**	4.63	2.69	2.80	−1.84	0.785 (0.522)	0.41	0.55	0.77	0.63	0.59
**15_47**	−4.63	−2.85	−3.60	0.77	0.752 (0.571)	0.35	0.51	0.75	0.65	0.57

Values of potency difference, multiple activity similarity and structure similarity are indicated. Mean SmALI values are in parenthesis.
[a] T1–T4 are the corresponding targets in each family and are defined in Table S1 of the Supplementary data.
[b] Multiple activity similarity.
[c] Mean structure similarity.
[d] Not applicable.

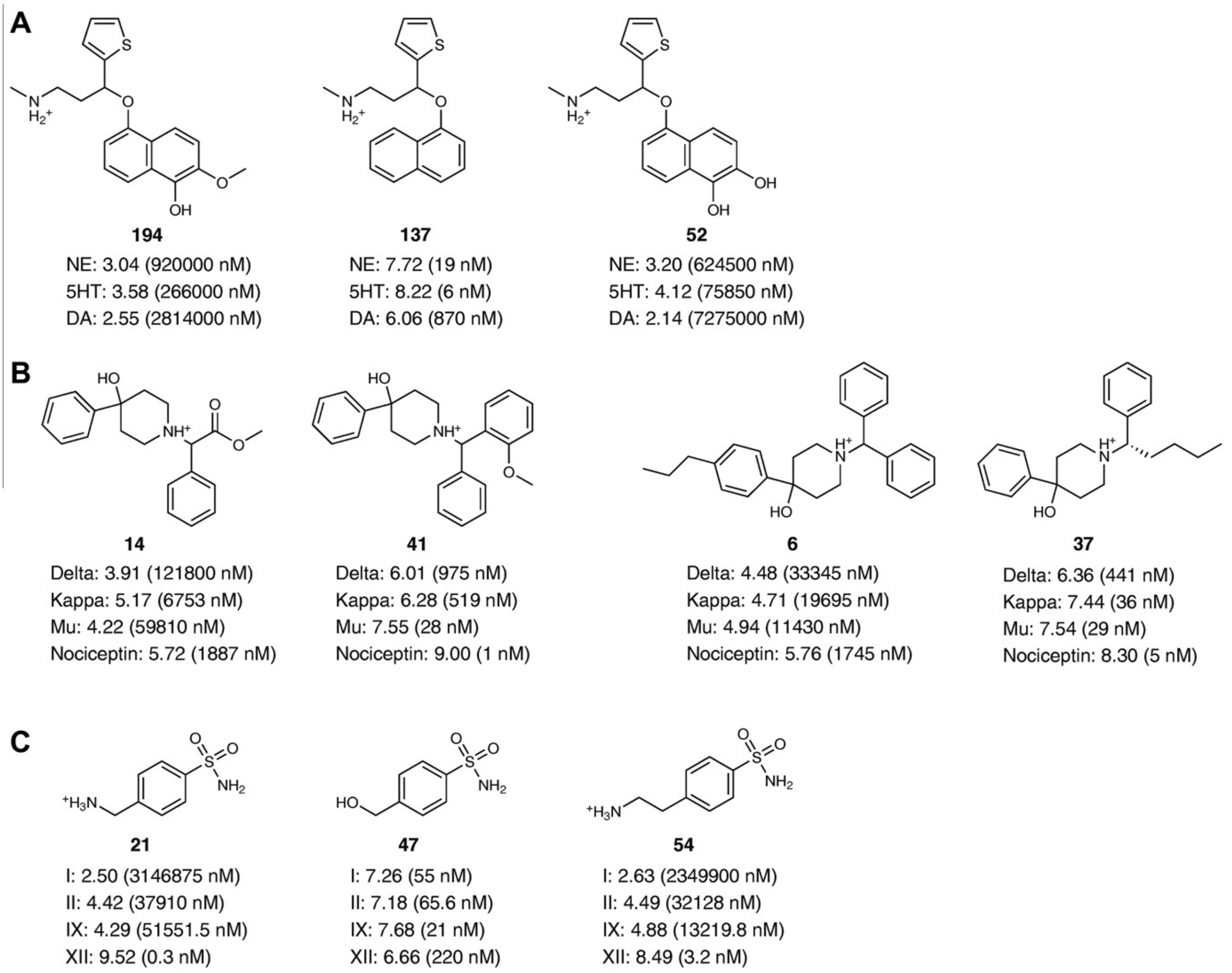

Figure 3. Examples of multi-target activity cliffs identified in the SmAS maps that is, pairs of compounds with high structure similarity and different bioactivity profile across different targets: (A) monoamine transporters (**137_194**, **52_137**); (B) opioid receptors (**14_41**, **6_37**); and (C) carbonic anhydrases (**21_47**, **47_54**). The pK_i of each compound for the three or four targets is indicated (the K_i value is in parenthesis). The position of the molecule pairs in the SmAS maps is indicated in Figure 2. The values of multiple activity similarity and structure similarity are summarized in Table 2. See text for details.

inhibitors had slightly lower multiple activity similarities (as compared to the opioid receptors antagonists and carbonic anhydrase inhibitors) as indicated by the mean and median values and other statistics in Table 1. Overall, these results indicate a slightly lower activity similarity profile for the data set of 299 compounds tested across the norepinephrine, serotonine, and dopamine transporters. For all target families, however, some low values of multiple activity similarity were observed (⩽0.661) indicating the presence of pairs of compounds with very different bioactivity profile across the corresponding individual targets.

Figure 2 shows the consensus SmAS maps for the three data sets (each data point represents a pairwise comparison): monoamine transporter inhibitors with 44,551 data points (Fig. 2A); opioid receptor antagonists with 4753 pairwise comparisons (2B); and carbonic anhydrase inhibitors with 4560 pairwise comparisons (2C).

Overall, the distribution of the data points is different for each data set. For example, for the monoamine transporter inhibitors, data points in Figure 2A are shifted, in general, towards low mean similarity values. In contrast, most of the data points for the opioid receptor antagonists are located towards the middle of the SmAS map (Fig. 2B). Clearly, this is because each data set studied in this work has different intra-molecular similarity (Table S2). The set of monoamine transporter inhibitors considered in this study are more diverse than the opioid receptor antagonists for example, the mean of the mean similarities are 0.249 and 0.495, respectively (Table S2). The carbonic anhydrase inhibitors have intermediate diversity (mean of the mean similarities of 0.345).

The SmAS maps can be analyzed quantitatively dividing the plots in four major quadrants using two thresholds; one for structure similarity and the second for multiple activity similarity (*shallow* and *deep* activity cliffs can be further differentiated by using a second threshold for multiple activity similarity that distinguishes cliffs with large and exceptionally large activity difference, respectively).[9,10,12] It is worth noting that there is not a unique criterion to impose these thresholds. For example, the different diversity of each data set considered in this work (vide supra) would influence the value of the threshold to define high/low structure similarity for each set. Likewise, the different distribution of multiple activity similarities for each set (Table 1) will affect the criteria to define a unique threshold for high/low activity profile similarity. In any case, after imposing thresholds, each SmAS map can be further characterized by employing different measures as the authors have previously reported.[9,10,12] Despite the fact there are challenges to quantify these plots arising from the different criteria to impose

Table 3
Representative consensus multi-target scaffold hops identified in the SmAS maps

Pair	ΔpK_i T1[a]	ΔpK_i T2	ΔpK_i T3	ΔpK_i T4	mAS[b]	Radial	Atom pairs	MACCS	piDAPH3	Mean sim[c]
Monoamine transporter inhibitors (MT)										
**49_152**	0.17	0.46	0.36	NA[d]	0.998 (0.003)	0.04	0.17	0.28	0.42	0.23
**44_224**	0.00	−0.51	−0.47	NA	0.998 (0.002)	0.04	0.17	0.47	0.00	0.17
**44_148**	0.43	−0.32	0.09	NA	0.998 (0.002)	0.07	0.18	0.47	0.00	0.18
**156_197**	−0.46	−0.22	−0.40	NA	0.998 (0.002)	0.06	0.14	0.37	0.00	0.14
Opioid receptor antagonists (OR)										
**31_54**	0.12	0.00	0.17	−0.45	0.999 (0.002)	0.07	0.29	0.61	0.46	0.36
**54_84**	0.06	0.60	0.14	0.60	0.996 (0.006)	0.09	0.28	0.54	0.48	0.34
**24_74**	−0.17	0.03	−0.12	0.34	0.999 (0.002)	0.05	0.32	0.60	0.60	0.39
**26_63**	−0.22	−0.27	−0.07	0.11	0.999 (0.001)	0.07	0.28	0.54	0.00	0.22
Carbonic anhydrase inhibitors (CA)										
**27_82**	0.19	0.11	−0.06	0.10	1.000 (0.000)	0.10	0.22	0.51	0.39	0.31
**6_37**	0.14	0.26	0.27	−0.36	0.999 (0.001)	0.05	0.06	0.51	0.36	0.25
**6_59**	0.12	0.20	0.30	0.33	0.999 (0.001)	0.07	0.13	0.54	0.29	0.26
**59_90**	−0.36	−0.02	0.24	0.26	0.999 (0.002)	0.10	0.28	0.54	0.48	0.35

Values of potency difference, multiple activity similarity and structure similarity are indicated. Mean SmALI values are in parenthesis.
[a] T1–T4 are the corresponding targets in each family and are defined in Table S1 of the Supplementary data.
[b] Multiple activity similarity.
[c] Mean structure similarity.
[d] Not applicable.

the thresholds, the SmAS maps are very intuitive tools to navigate through the multi-target activity landscapes which is the main focus of the next sections. Quantitative characterization of the activity landscapes is performed using the SmALI measure (vide infra).

3.2. Characterization of multi-target activity cliffs

Table 2 lists examples of consensus multi-target activity cliffs for each target family. For each representative pair of compounds, Table 2 lists the potency difference for all three (MT) or four targets (OR, CA), the multiple activity similarity, the corresponding structure similarity values for the different fingerprints as well as the mean structure similarity (see Section 2). For all pairs of compounds in this table, the values of the multiple activity similarity are low for example, lower than the corresponding L95 (lower limit of the mean at 95% of confidence) value of the distribution of multiple activity similarities (Table 1). These low values are in agreement with the large potency differences for all the corresponding three or four targets (Table 2). In other words, *small* multiple activity similarity indicates *different bioactivity profile* across the different targets. All pairs of compounds in Table 2 have, however, high structure similarity values as captured by different structure representations for example, the mean structure similarity value of each pair of compounds is higher than the corresponding U95 value of the distribution of the mean similarity values (Table S2). Figure 2A–C shows the position of selected multi-target activity cliffs in the SmAS maps of each target family; pairs of compounds **52_137** and **137_194** in Figure 2A; **6_37** and **14_41** in Figure 2B; and **47_54** and **21_47** in Figure 2C. This figure clearly shows that all activity cliffs are located in the lower-right region of each SmAS map (*roughly* in region IV of the prototype map in Fig. 1). We want to emphasize that, for each target family, additional examples of activity cliffs can be identified on the lower-right region of each SmAS map in Figure 2.

Figure 3A–C shows a side-by-side comparison of the chemical structures for selected activity cliffs of the MT (Fig. 3A), OR (3B), and CA (3C) data sets, respectively. The bioactivity profile across the three or four targets for each target family is also shown. For example, the pairs of compounds **137_194** and **52_137** for the MT target family in Figure 3A have a very different activity profile across the NE, 5HT, and DA transporters with large potency differences (more than three log units for these pairs). These potency differences are well captured by the low multiple activity similarity values (0.550 and 0.576, respectively, Table 2). However, the chemical structures of these pairs of compounds are quite similar as clearly observed in Figure 3A. Similar conclusions can be obtained for the representative multi-target activity cliffs **14_41** and **6_37** for the OR target family (Fig. 3B), and **21_47** and **47_54** for the CA target family (Fig. 3C). Despite the fact the chemical structures of these pairs of compounds are quite similar, all pairs have a different activity profile across the corresponding four tested targets namely, delta, kappa, mu, and nociceptin receptors (Fig. 3B) and carbonic anhydrases I, II, IX, and XII (Fig. 3C). In theses examples, the potency difference across the four targets is more than one log unit. For the pairs of compounds **21_47** and **47_54** (Fig. 3C) it is interesting to note the large impact on the MACCS keys/Tanimoto similarity of the presence/absence of the OH group (Table 2). Similar observations have been made for other compound data sets having hydroxyl groups.[38]

3.3. Multi-target scaffold hops

Scaffold hops are not commonly analyzed in activity landscape modeling that is frequently focused on the analysis of activity cliffs. Despite the fact that activity cliffs provide essential information in the SAR,[1] scaffold hoping is also a useful approach in drug design to ‘jump’ in different areas of chemical

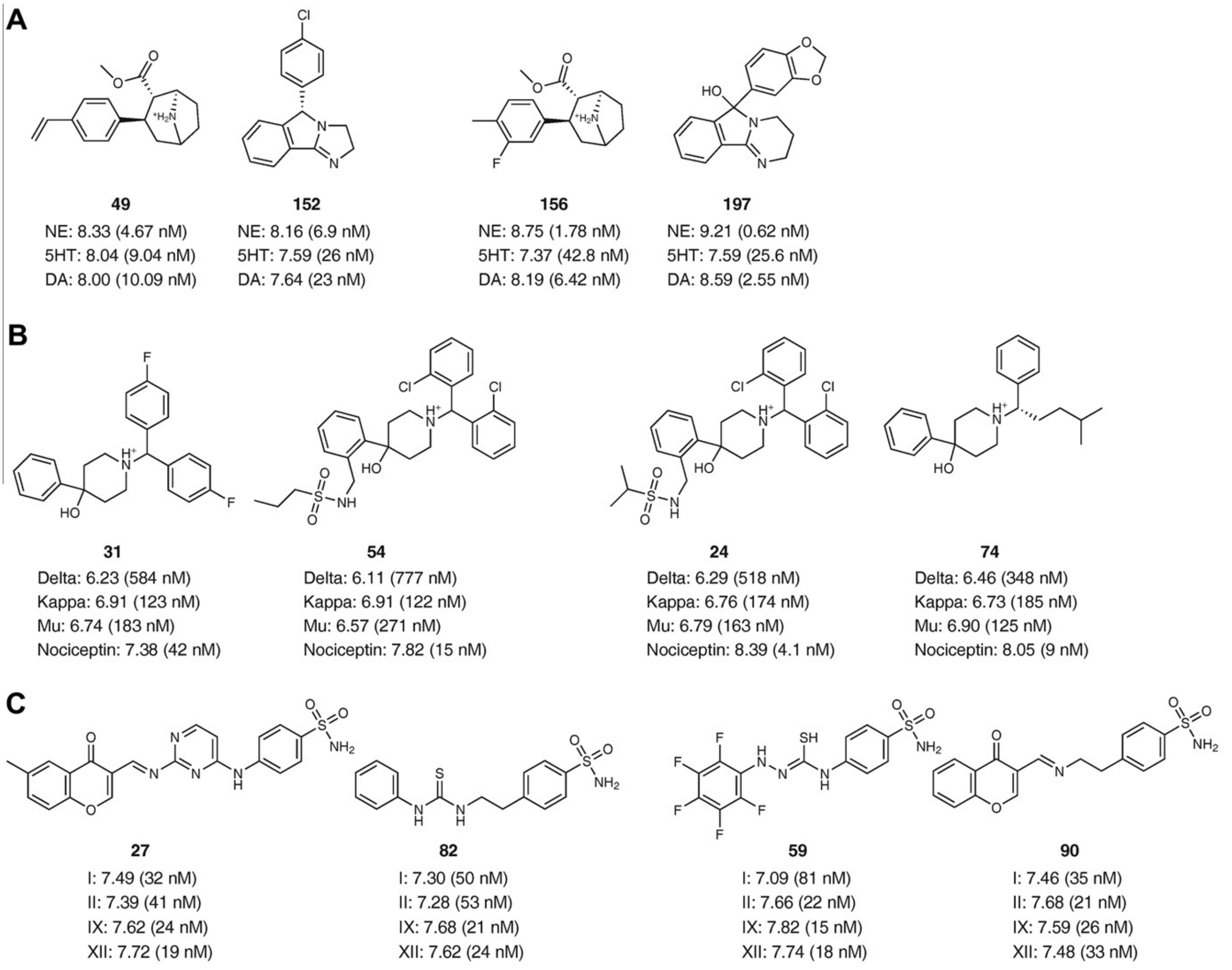

Figure 4. Representative multi-target scaffold hops identified in the SmAS maps that is, pairs of compounds with low structure similarity and similar bioactivity profile across different targets: (A) monoamine transporters (**49_152**, **156_197**); (B) opioid receptors (**31_54**, **24_74**); and (C) carbonic anhydrases (**27_82**, **59_90**). The pK_i of each compound for the three or four targets is indicated (the K_i value is in parenthesis). The position of the molecule pairs in the SmAS maps is indicated in Figure 2. The values of multiple activity similarity and structure similarity are summarized in Table 3. See text for details.

space.[31,39] Scaffold hops are characterized by having structurally different templates but equivalent biological activity. Scaffold hopping is used in drug discovery and optimization for example to avoid some undesirable ADME-tox properties; move from complex natural products to more easily synthesizable small molecules or for Intellectual Property (IP) reasons.[39,40] Table 3 lists representative consensus multi-target scaffold hops identified in the SmAS maps for each target family. Table 3 also lists the potency difference for all three or four targets, the multiple activity similarity, and structure similarity values for each pair. In contrast to multi-target activity cliffs, the values of the multiple activity similarity are high for example, higher than the corresponding U95 value of the distribution of multiple activity similarity values (Table 1). These high values are in agreement with the low potency differences for all the corresponding three or four targets, respectively (Table 3). Of note, in these examples, the potency difference across the three or four targets is equal or less than 0.6 log units. In other words, *high* values of multiple activity similarity indicate *similar bioactivity profile* across the different targets. All pairs of compounds in Table 3 have, however, low structure similarity values as indicated by different representations for example, the mean structure similarity value of each pair of compounds is very close or lower than the corresponding the L95 value of the distribution of the mean similarity values (Table S2). Figure 2A–C shows the position in the SmAS maps of multi-target scaffold hops selected from Table 3; pairs of compounds **49_152** and **156_197** in Figure 2A; **31_54** and **24_74** in Figure 2B; **27_82** and **59_90** in Figure 2C. Scaffold hops are clearly located in the upper-left region of the maps (*roughly* in region I of the prototype map in Fig. 1). It should be noted that, overall, several data points are located in the scaffold hope region of the SmAS maps. Similar results have been obtained for SAS maps for single targets.[9,10,12]

Figure 4A–C shows a side-by-side comparison of the chemical structures of representative scaffold hops of the MT (Fig. 4A), OR (4B), and CA (4C) data sets, respectively. The activity profile across the three or four corresponding targets is also shown. It is clear from Table 3 and Figure 4A–C that pairs of compounds such as **49_152** (Fig. 4A), **31_54** (4B), and **27_82** (4C) have different chemical structures. However, the activity profile is similar across the corresponding three monoamine transporters, four opioid receptors, and four carbonic anhydrases, respectively. Similar conclusions can be obtained for other pairs of compounds presented in Table 3 and Figure 4A–C.

Table 4
Representative pairs of compounds identified in smooth regions of the SmAS maps

Pair	ΔpK_i T1[a]	ΔpK_i T2	ΔpK_i T3	ΔpK_i T4	mAS[b]	Radial	Atom pairs	MACCS	piDAPH3	Mean sim[c]
Monoamine transporter inhibitors (MT)										
**68_250**	−0.51	0.00	0.04	NA[d]	0.999 (0.013)	1.00	0.74	0.95	0.99	0.92
**103_283**	0.07	−0.10	0.06	NA	1.000 (0.000)	0.50	0.99	0.96	0.92	0.84
**206_290**	0.07	−0.06	−0.37	NA	0.999 (0.006)	0.49	0.88	0.98	0.96	0.83
**60_282**	0.10	0.44	−0.20	NA	0.998 (0.018)	1.00	0.73	0.95	0.87	0.89
Opioid receptor antagonists (OR)										
**10_13**	−0.11	0.23	−0.41	0.15	0.999 (0.004)	0.47	0.74	0.90	0.79	0.73
**7_10**	0.05	0.18	0.00	−0.34	0.999 (0.004)	0.47	0.74	0.96	0.82	0.75
**8_18**	0.06	−0.07	0.12	0.02	1.000 (0.000)	0.48	0.62	0.96	0.93	0.74
**48_79**	0.30	−0.30	0.00	−0.10	0.999 (0.005)	0.58	0.80	0.98	0.87	0.81
Carbonic anhydrase inhibitors (CA)										
**12_90**	0.07	−0.46	−0.15	−0.13	0.999 (0.005)	0.43	0.84	0.98	0.92	0.79
**49_95**	0.54	0.30	0.06	0.01	0.998 (0.006)	0.36	0.54	0.92	0.93	0.69
**20_31**	−0.18	0.19	0.18	0.23	0.999 (0.004)	0.33	0.88	0.97	0.83	0.75
**27_33**	0.06	−0.09	−0.06	−0.08	1.000 (0.000)	0.50	0.85	0.99	0.98	0.83

Values of potency difference, multiple activity similarity and structure similarity are indicated. Mean SmALI values are in parenthesis.
[a] T1–T4 are the corresponding targets in each family and are defined in Table S1 of the Supplementary data.
[b] Multiple activity similarity.
[c] Mean structure similarity.
[d] Not applicable.

3.4. Smooth regions in the multi-target activity landscapes

Table 4 presents pairs of compounds that exemplify gently sloped and smooth regions in the multi-target activity landscape of each target family. The gently sloped regions are associated with pairs of compounds with high structure similarity and similar activity profile[23,29] across the different targets. Indeed, the multiple activity similarity of the pairs of compounds in Table 4 are high for example, higher than the corresponding U95 value of the distribution of mAS values (Table 1). Similar to the discussion above for the scaffold hops, high values of the multiple activity similarity are in agreement with the low potency differences for all the corresponding three or four targets, respectively (Table 4). In contrast to the scaffold hops, all pairs of compounds in Table 4 have high structure similarity as captured by the different 2D and 3D representations. Figure 2A–C shows the position of selected pairs of compounds for each target family in the smooth region of the landscape of the SmAS maps; pairs of compounds **103_283** and **68_250** in Figure 2A; **7_10** and **10_13** in Figure 2B; **12_90** and **49_95** in Figure 2C. These pairs are located in the upper-right region of the plot (*roughly* in region II of the prototype SmAS in Fig. 1). Figure 5A–C shows a side-by-side comparison of the chemical structures of selected pairs. This figure clearly shows that pairs of compounds such as **68_250** (Fig. 5A), **10_13** (5B), and **12_90** (5C) have very similar chemical structures and also similar activity profile across the three monoamine transporters, four opioid receptors, and four carbonic anhydrases, respectively.

3.5. Multi-target activity cliffs and scaffold hops with SmALI

The SALI parameter is a metric to easily detect activity cliffs. This index has been used to represent SARs as matrices and as graphs (SALI networks).[5] The authors recently introduced an extension of this approach, the *mean SALI* value that combines the information from different structure representations to detect consensus activity cliffs.[9,13] As a follow up of our work, herein, we computed the mean SmALI for each of the three target families as described in the Methods. A summary of the distribution of the mean SmALI values for each target is presented in Table 5. The overall distribution of the SmALI values depends on each target family. In general, the monoamine transporters had the highest mean SmALI values as indicated by the mean and median of the distribution (Table 5). In contrast, the opioid receptor antagonists had the smallest mean SmALI values. These results suggest more agreement between the SAR of the compounds tested across the four opioid receptors, as compared to the SAR of compounds tested across the other two target families. The corresponding mean SmALI values for selected molecule pairs are indicated in Tables 2–4. Multi-target activity cliffs such as **137_194**, **52_137** for MT; **14_41** and **6_37** for OR; and **21_47** and **47_54** for CA, have high mean SmALI values relative to their corresponding distribution (Table 2). Of note, these values are higher than the corresponding U95 (upper limit of the mean at 95% of confidence) values of mean SmALI for each target family (Table 5). Of note, multiple-target activity cliffs were also identified for the same set of monoamine transporter inhibitors using TAD maps.[13] In sharp contrast, the mean SmALI values for the scaffold hops in Table 3 are very low, lower than the corresponding L95 (lower limit of the mean at 95% of confidence) value of the corresponding distribution of mean SmALI values (Table 5). The low mean SmALI values for the pair of compounds in Table 3 such as **49_152** for MT; **31_54** for OR; and **27_82** for CA are in agreement with the scaffold hop characteristic of these pairs.

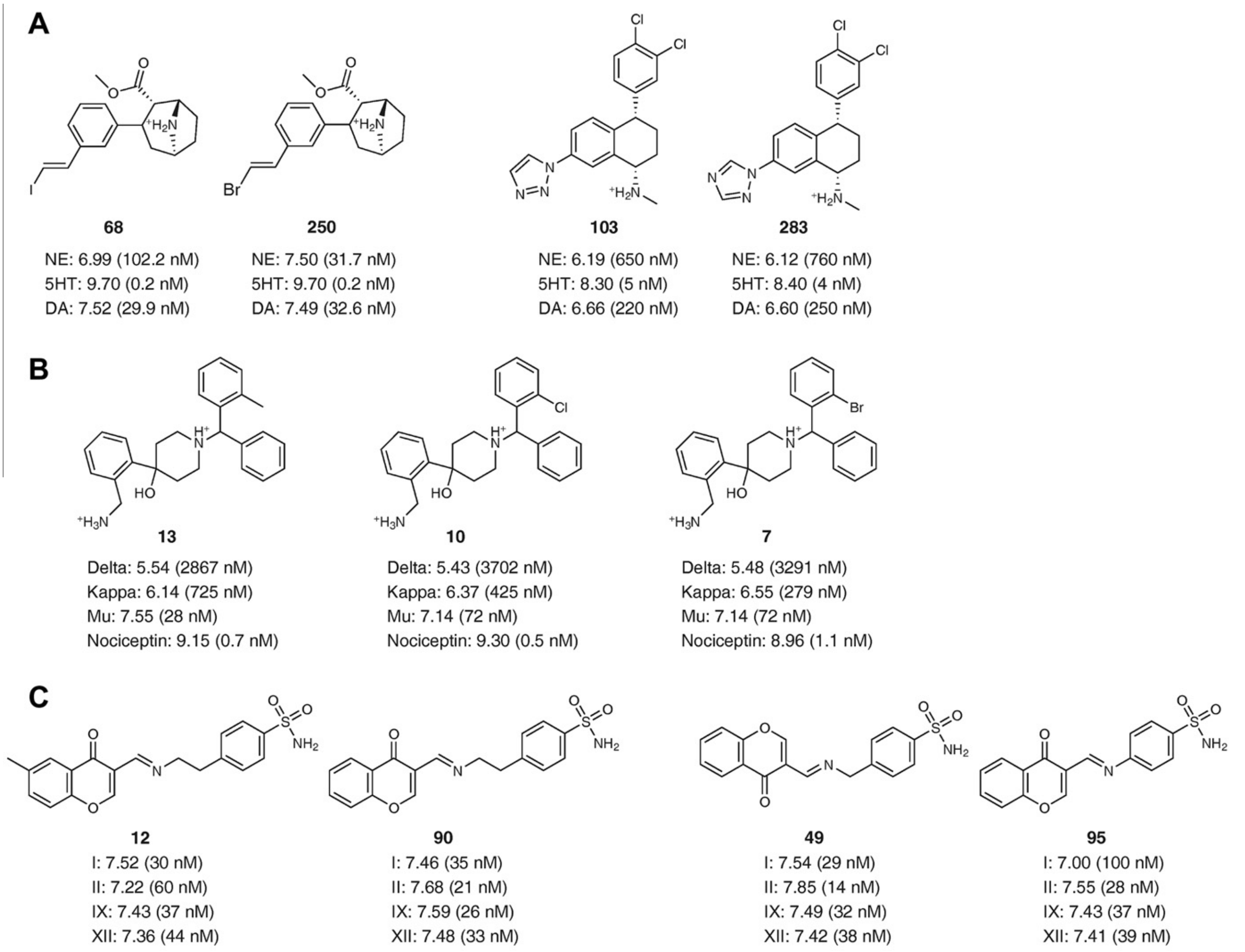

Figure 5. Examples of pairs of compounds in smooth regions of the SmAS maps that is, pairs of compounds with high structure similarity and similar bioactivity profile across different targets: (A) monoamine transporters (**68_250**, **103_283**); (B) opioid receptors (**10_13**, **7_10**); and (C) carbonic anhydrases (**12_90**, **49_95**). The pK_i of each compound for the three or four targets is indicated (the K_i value is in parenthesis). The position of the molecule pairs in the SmAS maps is indicated in Figure 2. The values of multiple activity similarity and structure similarity are summarized in Table 4. See text for details.

Table 5
Distribution of mean SmALI values for each data set

Set	Max	Q3[a]	Median	Q1[b]	Min	U95[c]	Mean	L95[c]	STD
MT	1.113	0.204	0.079	0.037	0	0.155	0.154	0.152	0.172
OR	0.407	0.084	0.038	0.018	0	0.063	0.061	0.059	0.062
CA	1.146	0.146	0.070	0.028	0	0.102	0.099	0.097	0.094

[a] Third quartile.
[b] First quartile.
[c] 95% Confidence of the mean Upper (U95) and Lower (L95) limits.

4. Conclusions

We present the Structure multiple Activity Similarity (SmAS) map and Structure multiple Activity Landscape Index (SmALI) as general approaches for the systematic description of the SAR of compound data sets with bioactivity data against multiple targets. Both approaches describe the SAR in a pairwise manner and are extensions of the SAS Maps and SALI measure initially proposed for single targets. The SmAS maps and SmALI metric are based on the multiple activity similarity measure which captures with a single number, the similarity of the bioactivity profile of a pair of compounds screened across two or more targets. In order to illustrate the use of SmAS maps and the SmALI metric, we applied these approaches to explore the activity landscapes of benchmark sets of compounds tested with three different target families namely, 299 compounds tested across three monoamine transporters; 98 compounds tested across four opioid receptors and 96 compounds tested across four carbonic anhydrases. In order to reduce the dependence of the activity landscape with molecular representation, an aggregated similarity measure was employed by combining the similarity values of four different 2D and 3D fingerprints. Of note, the SmAS maps and the SmALI metric can be applied with any representation (or set of selected representations) or protocol to aggregate similarity measures.

For the three sets of compounds, multi-target activity cliffs, scaffold hops as well as pairs of compounds in continuous regions of the multi-target activity landscape were easily identified in the SmAS maps. Consensus activity cliffs were quantitatively characterized using the mean SmALI metric that captures information from several different 2D and 3D representations. The consensus SmAS maps and mean SmALI metric presented in this work are useful and complementary tools to quantify and navigate through multi-target activity landscapes. The predictive capabilities of current and/or novel approaches used in activity landscape modeling

to anticipate the SAR of new molecules in a prospective manner is a major perspective of this work.

Acknowledgments

We are most grateful to Dr. Gerald M. Maggiora for motivating much of our work on activity landscape modeling and insightful discussions. The authors also thank Professor Dr. Jürgen Bajorath for making available the data sets used in this study. This work was supported by the State of Florida, Executive Office of the Governor's Office of Tourism, Trade, and Economic Development and the Multiple Sclerosis National Research Institute.

Supplementary data

Supplementary data associated with this article can be found, in the online version, at doi:10.1016/j.bmc.2011.11.051.

References and notes

1. Maggiora, G. M. *J. Chem. Inf. Model.* **2006**, *46*, 1535.
2. Scior, T.; Medina-Franco, J. L.; Do, Q. T.; Martínez-Mayorga, K.; Yunes Rojas, J. A.; Bernard, P. *Curr. Med. Chem.* **2009**, *16*, 4297.
3. Bajorath, J.; Peltason, L.; Wawer, M.; Guha, R.; Lajiness, M. S.; Van Drie, J. H. *Drug Discovery Today* **2009**, *14*, 698.
4. Peltason, L.; Bajorath, J. *J. Med. Chem.* **2007**, *50*, 5571.
5. Guha, R.; VanDrie, J. H. *J. Chem. Inf. Model.* **2008**, *48*, 646.
6. Guha, R.; Van Drie, J. H. *J. Chem. Inf. Model.* **2008**, *48*, 1716.
7. Wawer, M.; Peltason, L.; Weskamp, N.; Teckentrup, A.; Bajorath, J. *J. Med. Chem.* **2008**, *51*, 6075.
8. Shanmugasundaram, V.; Maggiora, G. M. Abstract of Papers, 222nd National Meeting of the American Chemical Society, Chicago, IL; American Chemical Society: Washington, DC, 2001; Abstract CINF-032.
9. Medina-Franco, J. L.; Martínez-Mayorga, K.; Bender, A.; Marín, R. M.; Giulianotti, M. A.; Pinilla, C.; Houghten, R. A. *J. Chem. Inf. Model.* **2009**, *49*, 477.
10. Pérez-Villanueva, J.; Santos, R.; Hernández-Campos, A.; Giulianotti, M. A.; Castillo, R.; Medina-Franco, J. L. *Bioorg. Med. Chem.* **2010**, *18*, 7380.
11. Pérez-Villanueva, J.; Santos, R.; Hernández-Campos, A.; Giulianotti, M. A.; Castillo, R.; Medina-Franco, J. L. *Med. Chem. Comm.* **2011**, *2*, 44.
12. Yongye, A.; Byler, K.; Santos, R.; Martínez-Mayorga, K.; Maggiora, G. M.; Medina-Franco, J. L. *J. Chem. Inf. Model.* **2011**, *51*, 1259.
13. Medina-Franco, J. L.; Yongye, A. B.; Pérez-Villanueva, J.; Houghten, R. A.; Martínez-Mayorga, K. *J. Chem. Inf. Model.* **2011**, *51*, 2427.
14. Peltason, L.; Hu, Y.; Bajorath, J. *ChemMedChem* **2009**, *4*, 1864.
15. Wassermann, A. M.; Peltason, L.; Bajorath, J. *ChemMedChem* **2010**, *5*, 847.
16. Dimova, D.; Wawer, M.; Wassermann, A. M.; Bajorath, J. *J. Chem. Inf. Model.* **2011**, *51*, 258.
17. Medina-Franco, J. L.; Martínez-Mayorga, K.; Bender, A.; Scior, T. *QSAR Comb. Sci.* **2009**, *28*, 1551.
18. Iyer, P.; Bajorath, J. *Chem. Biol. Drug Des.* **2011**, *78*, 778.
19. Peltason, L.; Bajorath, J. *Chem. Biol.* **2007**, *14*, 489.
20. Medina-Franco, J. L.; Martínez-Mayorga, K.; Giulianotti, M. A.; Houghten, R. A.; Pinilla, C. *Curr. Comput.-Aided Drug Des.* **2008**, *4*, 322.
21. Medina-Franco, J. L.; Yongye, A. B.; López-Vallejo, F. In Statistical Modeling of Molecular Descriptors in QSAR/QSPR; Matthias, D., Kurt, V., Danail, B., Eds.; Wiley-VCH, 2012; pp. 309–328.
22. Data sets obtained at http://www.limes.uni-bonn.de/forschung/abteilungen/Bajorath/labwebsite (accessed May, 2011).
23. Maggiora, G. M.; Shanmugasundaram, V. In *Chemoinformatics and Computational Chemical Biology Methods in Molecular Biology*; Bajorath, J., Ed.; Springer: New York, 2011; Vol. 672, pp. 39–100.
24. Rogers, D.; Hahn, M. *J. Chem. Inf. Model.* **2010**, *50*, 742.
25. Canvas, version 1.3, Schrödinger, LLC, New York, NY, 2010.
26. Molecular Operating Environment (MOE), version 2009.10, Chemical Computing Group Inc., Montreal, Quebec, Canada. http://www.chemcomp.com (accessed October 20, 2011).
27. The single low-energy conformation was obtained after 'washing' the compounds with MOE and energy-minimize the structures with the Merck Molecular Force Field 94x.
28. Jaccard, P. *Bull. Soc. Vaudoise Sci. Nat.* **1901**, *37*, 547.
29. Wassermann, A. M.; Wawer, M.; Bajorath, J. *J. Med. Chem.* **2010**, *53*, 8209.
30. Wawer, M.; Lounkine, E.; Wassermann, A. M.; Bajorath, J. *Drug Discovery Today* **2010**, *15*, 630.
31. Brown, N.; Jacoby, E. *Mini-Rev. Med. Chem.* **2006**, *6*, 1217.
32. Schneider, G.; Neidhart, W.; Giller, T.; Schmid, G. *Angew. Chem., Int. Ed.* **1999**, *38*, 2894.
33. Iyer, P.; Wawer, M.; Bajorath, J. *Med. Chem. Comm.* **2011**, *2*, 113.
34. Willett, P. *Drug Discovery Today* **2006**, *11*, 1046.
35. Chen, B.; Mueller, C.; Willett, P. *Molecular Informatics* **2010**, *29*, 533.
36. Medina-Franco, J. L.; Maggiora, G. M.; Giulianotti, M. A.; Pinilla, C.; Houghten, R. A. *Chem. Biol. Drug Des.* **2007**, *70*, 393.
37. Sastry, M.; Lowrie, J. F.; Dixon, S. L.; Sherman, W. *J. Chem. Inf. Model.* **2010**, *50*, 771.
38. Owen, J. R.; Nabney, I. T.; Medina-Franco, J. L.; López-Vallejo, F. *J. Chem. Inf. Model.* **2011**, *51*, 1552.
39. Ciapetti, P.; Giethlen, B. In *The Practice of Medicinal Chemistry*; Wermuth, C. G., Ed.; Elsevier, 2008; pp. 290–342.
40. López-Vallejo, F.; Castillo, R.; Yépez-Mulia, L.; Medina-Franco, J. L. *J. Biomol. Screening* **2011**, *16*, 862.
