# Supplementary material for: On the validity versus utility of activity landscapes: are all activity cliffs statistically significant?
Source: J Cheminform. 2014 Apr 2;6:11. doi: 10.1186/1758-2946-6-11 (PMC4021161; doi:10.1186/1758-2946-6-11)
Supplement: Additional file 1 — Code and data to reproduce figures. The ZIP file contains the R code and datasets required to perform the calculations and generate the figures for this article. It assumes you have R installed and are working on a Unix-like system. In addition you should have the ggplot2, grid, multicore and fingerprint R packages installed. To run the scripts execute run.sh from the command line. [file 1758-2946-6-11-S1.zip › si/bcg/2009 JCIM ActivityLandscape-links.pdf]

# Characterization of Activity Landscapes Using 2D and 3D Similarity Methods: *Consensus Activity Cliffs*

Jose L. Medina-Franco,*[,†,‡] Karina Martínez-Mayorga,[†] Andreas Bender,[§] Ray M. Marín,[||] Marc A. Giulianotti,[†] Clemencia Pinilla,[⊥] and Richard A. Houghten[†,⊥]

Torrey Pines Institute for Molecular Studies, 11350 SW Village Parkway, Port St. Lucie, Florida 34987, Instituto Nacional de Cancerologia, Av. San Fernando 22, Mexico City 14080, Mexico, Medicinal Chemistry Division and Pharma-IT Platform, Leiden/Amsterdam Center for Drug Research, Leiden University, 2333 CC Leiden, The Netherlands, Grupo de Química Teórica, Universidad Nacional de Colombia, Bogotá D. C., Colombia, and Torrey Pines Institute for Molecular Studies, 3550 General Atomics Court, San Diego, California 92121

Received October 13, 2008

Activity landscape characterization has been demonstrated to be a valuable tool in lead optimization, virtual screening, and computational modeling of active compounds. In this work, we present a general protocol to explore systematically the activity landscape of a lead series using 11 2D and 3D structural representations. As a test case we employed a set of 48 bicyclic guanidines (BCGs) with $\kappa$-opioid receptor binding affinity, identified in our group. MACCS keys, graph-based three point pharmacophores, circular fingerprints, ROCS shape descriptors, and the TARIS approach, that compares structures based on molecular electrostatic potentials, were employed as orthogonal descriptors. Based on 'activity cliffs' common to a series of descriptors, we introduce the concept of *consensus activity cliffs*. Results for the current test case suggest that the presence or absence of a methoxybenzyl group may lead to different modes of binding for the active BCGs with the $\kappa$-opioid receptor. The most active compound ($IC_{50}$ = 37 nM) is involved in a number of consensus cliffs making it a more challenge query for future virtual screening than would be expected from affinity alone. Results also reveal the importance of screening high density combinatorial libraries, especially in the "cliff-rich" regions of activity landscapes. The protocol presented here can be applied to other data sets to develop a consensus model of the activity landscape.

## INTRODUCTION

Understanding the structure−activity relationships (SAR) of a set of compounds with measured biological activity plays a key role in virtual screening and lead optimization. To this end, several computational techniques can be employed such as pharmacophore modeling, two-dimensional (2D) quantitative structure−activity relationships (QSAR), three-dimensional (3D) QSAR, rule-based methods, or neural networks to name a few.[1] However, several methodologies like traditional QSAR make assumptions that do not necessarily hold true and, thus, may present misleading results.[2−4] For example, one common assumption is that a lead series of compounds has a common binding mode or mechanism of action. For this reason understanding the *activity landscape* and early detection of *activity cliffs*[5] can be crucial to the success of computational models.[6] Similarly, characterizing the activity landscape will be critical in future ligand-based virtual screening campaigns (e.g., for selecting appropriate reference molecules for similarity-based virtual screening).[7]

* Corresponding author phone/fax (772)345-4685. E-mail: jmedina@tpims.org. Corresponding author address: Torrey Pines Institute for Molecular Studies, 11350 SW Village Parkway, Port St. Lucie, FL 34987.
† Torrey Pines Institute for Molecular Studies, Port St. Lucie FL.
‡ Instituto Nacional de Cancerologia.
§ Leiden University.
|| Universidad Nacional de Colombia.
⊥ Torrey Pines Institute for Molecular Studies, San Diego, CA.

The activity landscape has been compared to rolling hills or continuous SAR where small changes in molecular structure are associated with small changes in activity.[5] Such SARs are attractive for molecular-similarity analysis that is based on the similarity-property principle.[8,9] A discontinuous SAR or rugged activity landscape, however, is populated with molecules with small changes in structure but large changes in activity (i.e., activity cliffs).[5] Such landscapes are common in lead optimization. It can also happen that structurally diverse compounds have similar activity, which is the basis of scaffold hopping.[10−14] Additionally, active regions with wide variations in chemical structure but small variations in biological activity may suggest different binding modes or sites or may reveal the effect of additional mechanisms such as the interaction with membranes that are not typically considered in several modeling approaches.[6]

A major challenge when characterizing the chemical space and activity landscape of a set of compounds is the influence of the particular representation used.[7,15] An example is a pair of stereoisomers. If the potency difference is large, 2D methods will consider this pair as an activity cliff, while this is not necessarily the case if the three-dimensional arrangement of features is taken into account. In other words, certain representations for a given data set may lead to *apparent* activity cliffs. An approach to address this problem is to consider multiple representations[16] and derive a consensus model for the activity landscape.

10.1021/ci800379q CCC: $40.75 © 2009 American Chemical Society
Published on Web 01/30/2009

**Table 1.** Chemical Structures of Bicyclic Guanidines Obtained from PC-SCL and Biological Activity in a k-Opioid Receptor Binding Assay[19]

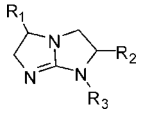


| | $R_1$ | $R_2$ | $R_3$ | $IC_{50}$ (nM) | $pIC_{50}$ |
|---|---|---|---|---|---|
| **1** | *S*-methyl | *S*-4-methoxybenzyl | 3-cyclohexylpropyl | 37 | 7.43 |
| **2** | *S*-methyl | *R*-4-methoxybenzyl | 2-norbornylethyl | 85 | 7.07 |
| **3** | *S*-methyl | *S*-4-methoxybenzyl | 2-norbornylethyl | 185 | 6.73 |
| **4** | *R*-cyclohexyl | *S*-4-methoxybenzyl | 2-norbornylethyl | 219 | 6.66 |
| **5** | *S*-methyl | *R*-4-methoxybenzyl | 1-adamantylethyl | 238 | 6.62 |
| **6** | *R*-cyclohexyl | *R*-4-methoxybenzyl | 2-norbornylethyl | 276 | 6.56 |
| **7** | *S*-cyclohexyl | *S*-4-methoxybenzyl | 4-(Me)-cyclohexylmethyl | 336 | 6.47 |
| **8** | *R*-cyclohexyl | *R*-4-methoxybenzyl | 1-adamantylethyl | 341 | 6.47 |
| **9** | *R*-cyclohexyl | *R*-isobutyl | 2-norbornylethyl | 359 | 6.44 |
| **10** | *R*-cyclohexyl | *R*-4-methoxybenzyl | 4-(Me)-cyclohexylmethyl | 362 | 6.44 |
| **11** | *S*-cyclohexyl | *S*-cyclohexyl | 4-(Me)-cyclohexylmethyl | 365 | 6.44 |
| **12** | *S*-cyclohexyl | *R*-4-methoxybenzyl | 4-(Me)-cyclohexylmethyl | 369 | 6.43 |
| **13** | *S*-methyl | *R*-4-methoxybenzyl | 4-(Me)-cyclohexylmethyl | 425 | 6.37 |
| **14** | *S*-methyl | *R*-4-methoxybenzyl | 3-cyclohexylpropyl | 502 | 6.30 |
| **15** | *R*-cyclohexyl | *S*-cyclohexyl | 2-norbornylethyl | 524 | 6.28 |
| **16** | *R*-cyclohexyl | *R*-4-methoxybenzyl | 3-cyclohexylpropyl | 547 | 6.26 |
| **17** | *S*-cyclohexyl | *S*-cyclohexyl | 2-norbornylethyl | 560 | 6.25 |
| **18** | *S*-cyclohexyl | *S*-4-methoxybenzyl | 2-norbornylethyl | 715 | 6.15 |
| **19** | *R*-cyclohexyl | *R*-isobutyl | 4-(Me)-cyclohexylmethyl | 738 | 6.13 |
| **20** | *R*-cyclohexyl | *S*-4-methoxybenzyl | 4-(Me)-cyclohexylmethyl | 804 | 6.09 |
| **21** | *S*-cyclohexyl | *R*-isobutyl | 2-norbornylethyl | 827 | 6.08 |
| **22** | *S*-cyclohexyl | *R*-4-methoxybenzyl | 2-norbornylethyl | 924 | 6.03 |
| **23** | *S*-cyclohexyl | *R*-isobutyl | 4-(Me)-cyclohexylmethyl | 999 | 6.00 |
| **24** | *R*-cyclohexyl | *S*-cyclohexyl | 4-(Me)-cyclohexylmethyl | 1140 | 5.94 |
| **25** | *S*-cyclohexyl | *R*-isobutyl | 1-adamantylethyl | 1206 | 5.92 |
| **26** | *S*-cyclohexyl | *S*-cyclohexyl | 1-adamantylethyl | 1492 | 5.83 |
| **27** | *S*-methyl | *S*-4-methoxybenzyl | 1-adamantylethyl | 1532 | 5.81 |
| **28** | *S*-methyl | *S*-4-methoxybenzyl | 4-(Me)-cyclohexylmethyl | 1568 | 5.80 |
| **29** | *R*-cyclohexyl | *R*-isobutyl | 1-adamantylethyl | 1747 | 5.76 |
| **30** | *R*-cyclohexyl | *R*-isobutyl | 3-cyclohexylpropyl | 1767 | 5.75 |
| **31** | *S*-methyl | *S*-cyclohexyl | 1-adamantylethyl | 1941 | 5.71 |
| **32** | *S*-methyl | *R*-isobutyl | 2-norbornylethyl | 2309 | 5.64 |
| **33** | *S*-methyl | *S*-cyclohexyl | 3-cyclohexylpropyl | 2479 | 5.60 |
| **34** | *R*-cyclohexyl | *S*-4-methoxybenzyl | 3-cyclohexylpropyl | 3456 | 5.46 |
| **35** | *S*-methyl | *R*-isobutyl | 1-adamantylethyl | 3641 | 5.44 |
| **36** | *S*-cyclohexyl | *R*-isobutyl | 3-cyclohexylpropyl | 3744 | 5.43 |
| **37** | *S*-cyclohexyl | *S*-4-methoxybenzyl | 3-cyclohexylpropyl | 3872 | 5.41 |
| **38** | *S*-methyl | *S*-cyclohexyl | 2-norbornylethyl | 4424 | 5.35 |
| **39** | *S*-cyclohexyl | *S*-cyclohexyl | 3-cyclohexylpropyl | 4482 | 5.35 |
| **40** | *S*-cyclohexyl | *S*-4-methoxybenzyl | 1-adamantylethyl | 4923 | 5.31 |
| **41** | *S*-cyclohexyl | *R*-4-methoxybenzyl | 3-cyclohexylpropyl | 5026 | 5.30 |
| **42** | *S*-methyl | *S*-cyclohexyl | 4-(Me)-cyclohexylmethyl | 5061 | 5.30 |
| **43** | *S*-methyl | *R*-isobutyl | 3-cyclohexylpropyl | 5436 | 5.26 |
| **44** | *S*-methyl | *R*-isobutyl | 4-(Me)-cyclohexylmethyl | 6477 | 5.19 |
| **45** | *R*-cyclohexyl | *S*-cyclohexyl | 3-cyclohexylpropyl | >10,000[a] | 5.00 |
| **46** | *S*-cyclohexyl | *R*-4-methoxybenzyl | 1-adamantylethyl | >10,000[a] | 5.00 |
| **47** | *R*-cyclohexyl | *S*-4-methoxybenzyl | 1-adamantylethyl | >10,000[a] | 5.00 |
| **48** | *R*-cyclohexyl | *S*-cyclohexyl | 1-adamantylethyl | >10,000[a] | 5.00 |

[a] For this work, the actual activity was approximated as $IC_{50}$ = 10,000 nM.

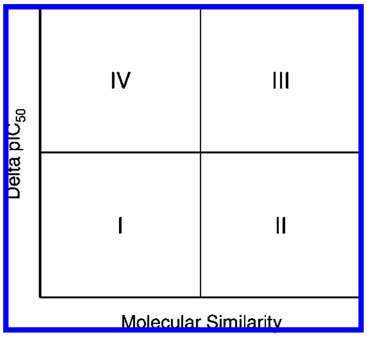


**Figure 1.** Four major regions in potency difference vs structural similarity plots. Regions I and II contain data pairs for scaffold hopping and smooth SAR, respectively. Region III indicates discontinuous SAR and activity cliffs. See text for details.

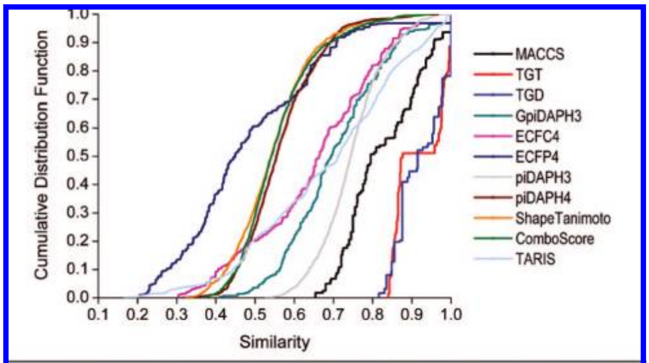


| Representation | Max | Q3 | Median | Q1 | Min | Mean | STD |
|---|---|---|---|---|---|---|---|
| MACCS | 1.00 | 0.92 | 0.80 | 0.75 | 0.65 | 0.83 | 0.10 |
| TGT | 1.00 | 0.98 | 0.88 | 0.86 | 0.84 | 0.92 | 0.06 |
| TGD | 1.00 | 0.98 | 0.92 | 0.88 | 0.82 | 0.92 | 0.06 |
| GpiDAPH3 | 1.00 | 0.79 | 0.69 | 0.62 | 0.41 | 0.71 | 0.12 |
| ECFC4 | 1.00 | 0.77 | 0.66 | 0.55 | 0.30 | 0.65 | 0.16 |
| ECFP4 | 1.00 | 0.63 | 0.45 | 0.37 | 0.20 | 0.49 | 0.18 |
| piDAPH3 | 1.00 | 0.80 | 0.74 | 0.69 | 0.53 | 0.75 | 0.08 |
| piDAPH4 | 0.97 | 0.62 | 0.56 | 0.50 | 0.32 | 0.57 | 0.10 |
| Shape Tanimoto | 0.92 | 0.60 | 0.54 | 0.48 | 0.32 | 0.55 | 0.10 |
| Combo Score/2 | 0.95 | 0.61 | 0.54 | 0.49 | 0.36 | 0.56 | 0.10 |
| TARIS | 1.00 | 0.83 | 0.71 | 0.54 | 0.17 | 0.68 | 0.19 |

**Figure 2.** Cumulative distribution functions of 1128 pairwise similarities using different 2D and 3D methods. The table summarizes the information of the distributions. Q3 and Q1 indicate the third and first quartiles, respectively.

In order to illustrate a new method to identify a consensus model and better understand the activity landscape of a data set and, in particular, to identify consensus activity cliffs, in this work we explore the structure−activity landscape of the 48 bicyclic guanidines (BCGs) as a test case (Table 1). The BCGs were selected from the screening of a positional scanning-synthetic combinatorial library and evaluated in a $\kappa$-opioid receptor binding assay. The set of molecules is part of our efforts to develop compounds for the treatment of pain and has been published in ref 17. In addition to the biological relevance, we found this set suitable for this study due to the presence of several pairs of stereoisomers. Additionally, the nature of the source of the compounds (e.g., combinatorial chemistry) that is attractive to characterize the activity landscape and identify activity cliffs because they are in highly dense regions of chemical space. The molecules show a wide range of activities with compound **1** being the most active ($IC_{50}$ = 37 nM) (Table 1). A total of 11 2D and 3D descriptors were employed to represent compounds. Using 3D molecular representations is particularly important in this set (as in many others) because of the occurrence of stereoisomers. Molecules were analyzed in a pairwise manner, and the computed molecular similarity was compared with the potency difference. Pairwise structure−activity relationships were visually depicted in 2D plots readily identified as continuous and discontinuous regions in the activity landscape. Quantitative analysis of the distribution of data points in these plots included a measure for the degree of consensus between two methods. We also computed the structure−activity landscape index (SALI) recently proposed to detect activity cliffs.[6,18] Here we extended this approach by applying a mean fusion rule of SALI values obtained for

| | MACCS | TGD | TGT | GpiDAPH3 | ECFC4 | ECFP4 | piDAPH3 | piDAPH4 | Shape Tanimoto | Combo Score/2 | TARIS |
|---|---|---|---|---|---|---|---|---|---|---|---|
| MACCS | 1.00 | | | | | | | | | | |
| TGD | 0.84 | 1.00 | | | | | | | | | |
| TGT | 0.88 | 0.94 | 1.00 | | | | | | | | |
| GpiDAPH3 | 0.69 | 0.69 | 0.75 | 1.00 | | | | | | | |
| ECFC4 | 0.69 | 0.52 | 0.54 | 0.62 | 1.00 | | | | | | |
| ECFP4 | 0.75 | 0.61 | 0.62 | 0.79 | 0.84 | 1.00 | | | | | |
| piDAPH3 | 0.53 | 0.53 | 0.58 | 0.79 | 0.40 | 0.56 | 1.00 | | | | |
| piDAPH4 | 0.53 | 0.43 | 0.46 | 0.64 | 0.47 | 0.61 | 0.82 | 1.00 | | | |
| Shape Tanimoto | 0.27 | 0.20 | 0.21 | 0.18 | 0.15 | 0.20 | 0.28 | 0.43 | 1.00 | | |
| Combo Score/2 | 0.38 | 0.30 | 0.32 | 0.31 | 0.28 | 0.34 | 0.41 | 0.56 | 0.82 | 1.00 | |
| TARIS | 0.40 | 0.46 | 0.46 | 0.32 | 0.28 | 0.24 | 0.38 | 0.34 | 0.21 | 0.32 | 1.00 |

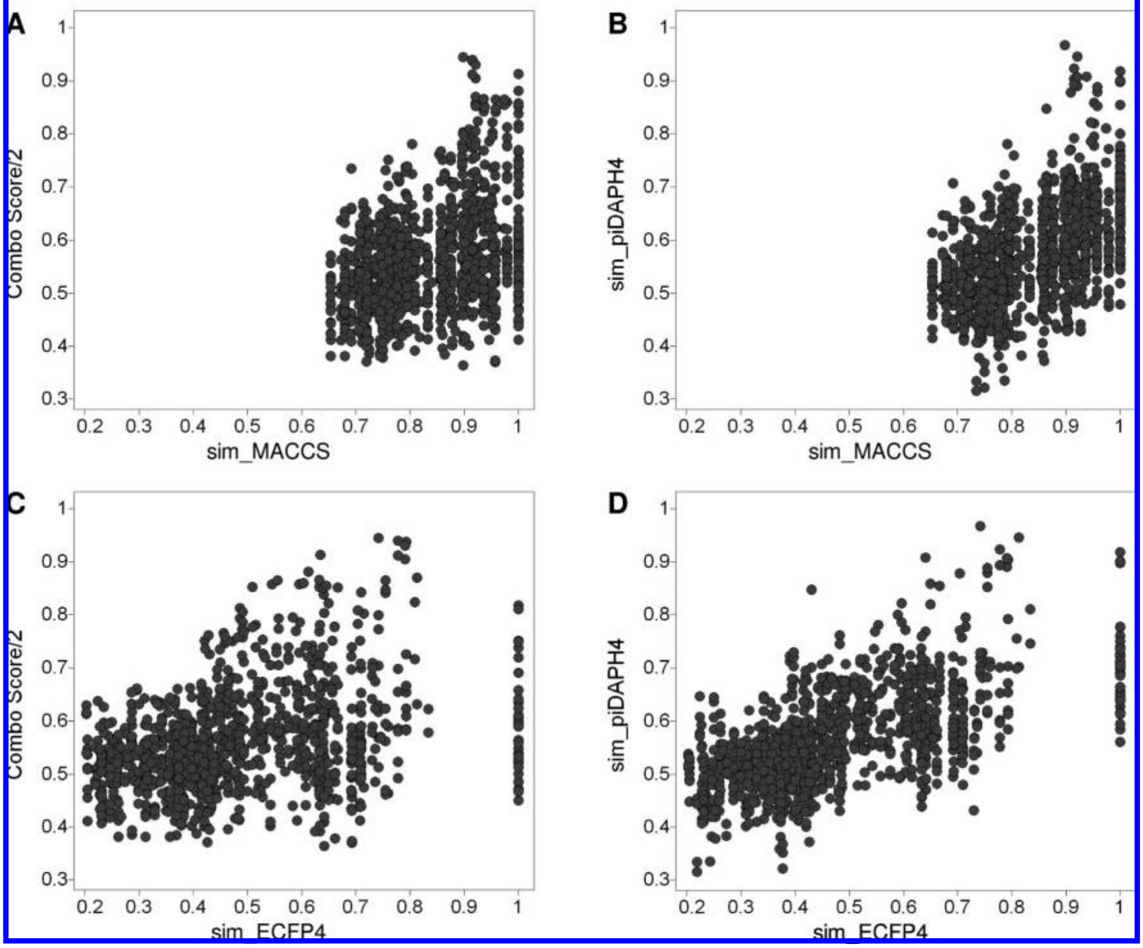


**Figure 3.** Correlation matrix for the pairwise similarities for the 2D and 3D methods considered in this work. Panels below depict the relationship between 2D and 3D similarity for selected structural representations: (A) MACCS keys vs Combo Score (scaled); (B) MACCS keys vs piDAPH4; (C) ECFP4 vs Combo Score (scaled); and (D) ECFP4 vs piDAPH4. Each data point indicates a pairwise comparison of 48 bicyclic guanidines (i.e., 1128 data points total).

a number of 2D and 3D representations. Results lead to the identification of consensus activity cliffs. The method described here, however, is not restricted to the test case or molecular representations employed. The emphasis in this work is on the use of multiple molecular representations to derive a consensus model of the activity landscape and identify consensus activity cliffs.

## METHODS

**Data Set.** A set of 48 BCGs obtained from a position scanning synthetic combinatorial library (PS-SCL) is presented in Table 1.[19] The conceptual and experimental framework of PS-SCL is described in detail elsewhere.[17,19,20] Table 1 lists the biological activity as the $IC_{50}$ value and the corresponding $-\log IC_{50}$ ($pIC_{50}$) obtained from a radioreceptor binding assay specific for the $\kappa$-opioid receptor.[19] Noteworthy, the activity ranges from 37 to greater that 10,000 nM.

**2D and 3D Structural Similarity.** Pairwise similarities were computed using Tanimoto coefficients[21] with the following 2D molecular representations as implemented in Molecular Operating Environment (MOE):[22] MACCS keys (166 bits), pharmacophore graph triangles (i.e., graph-based three point pharmacophores, GpiDAPH3), typed graph distances (TGD), and typed graph triangles (TGT) as well as PipelinePilot ECFC4 and ECFP4 circular fingerprints

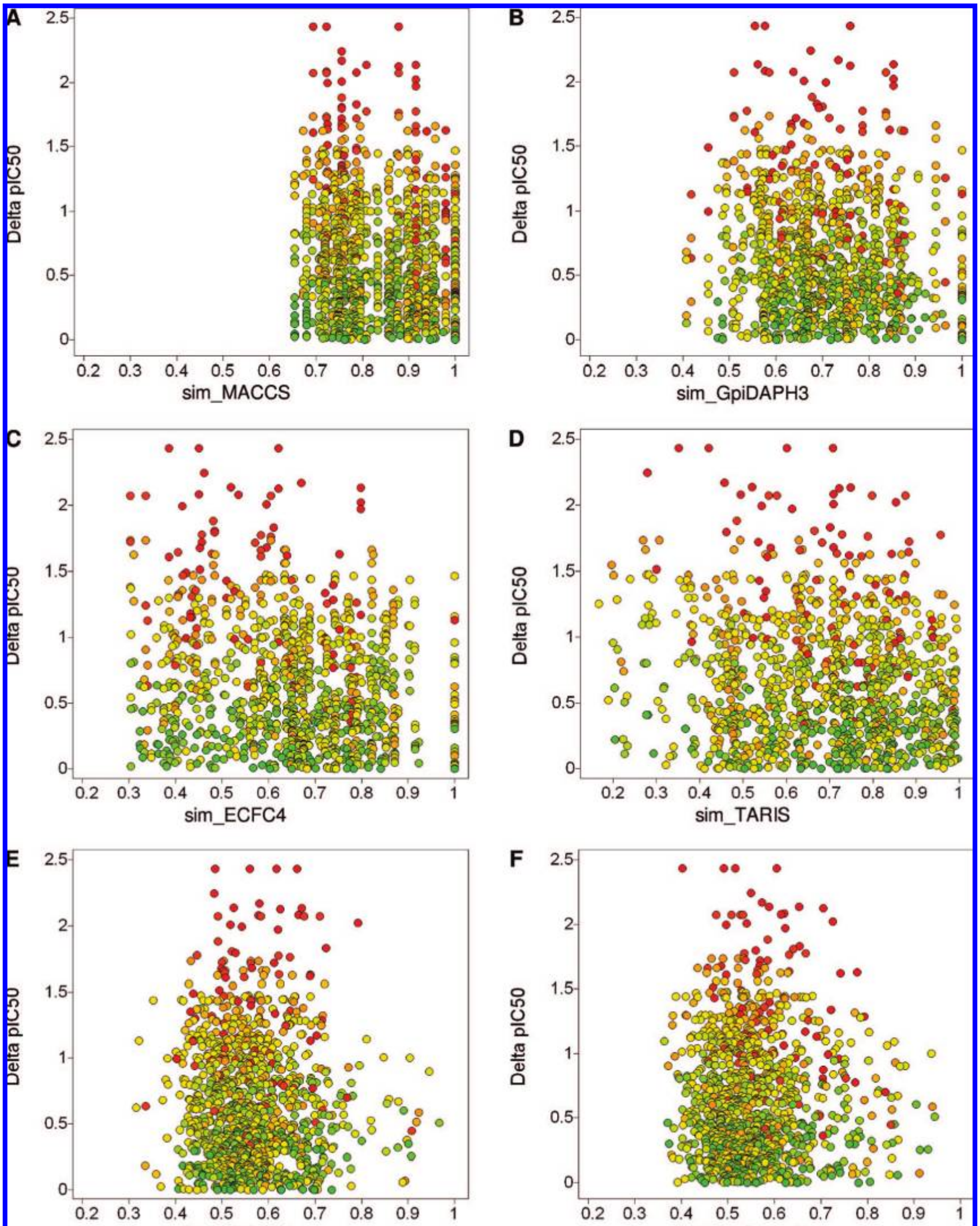


**Figure 4.** Absolute potency difference vs structural similarity with different structural representations. Each data point indicates a pairwise comparison of 48 bicyclic guanidines (i.e., 1128 data points total). Data points are color-coded by the activity of the most active compound in the pair using a continuous scale from green (less active) to red (most active). Each panel corresponds to a different structural representation: (A) MACCS keys; (B) GpiDAPH3; (C) ECFC4; (D) TARIS; (E) piDAPH4; and (F) Combo Score (scaled).

(ECFP4 fingerprints consider only the presence and absence of features and ECFC4 fingerprints consider the frequency of features). Descriptors were selected in a way that they showed maximally orthogonal behavior, as demonstrated in a recent study.[23,24] To compute 3D similarities, a single low-energy conformation was considered for each molecule obtained by superimposing all the molecules with **1**. This was accomplished using the Rapid Overlay of Chemical Structures (ROCS) software[25] and a multifusion approach[26] as previously described.[27] We have recently proposed a putative binding conformation (Y-shape) for the most active molecule (**1**), which has nanomolar activity.[27] Three-dimensional similarity values for each molecule were obtained using ROCS. The resulting Combo Score ("shape" and "color") was divided by two in order to obtain a number within the same scale as the other computed similarity values. The Tanimoto shape component of the Combo Score was also analyzed. Additional 3D similarity values were calculated (using the same conformations for each molecule) using the Tanimoto coefficient. These include the pharmacophore atom triangle (piDAPH3), pharmacophore atom quadruplet (piDAPH4) fingerprints implemented in MOE, and the recently developed Tree Analysis and Representation of Isopotential Surfaces (TARIS).[28] TARIS is a graph theoreti-

cal model used to compare molecular isopotential surfaces and measure molecular similarity. In this method, the molecular electrostatic potential (MEP) is represented with a weighted rooted tree that encodes geometrical information and topological relations of successive isopotential surfaces. The TARIS method is detailed elsewhere.[28] In this work, single point quantum chemical calculations were carried out at a HF/6−31G level with the Gaussian98 Rev. A11 program suite.[29] All the MEP 3D grids were obtained with the same resolution: 0.15 Å, i.e., each point in the grid is separated by 0.15 Å from the next in the *x*, *y*, and *z* directions. Since negative molecular isopotential surfaces have been shown to work better than the positive ones, the scan of the MEP was made from −0.2 to −0.02 au.[28]

**Pairwise Activity Relationships.** For each pair of bicyclic guanidines, potency differences were determined as the absolute difference between their $pIC_{50}$ values given in Table 1. On a relative scale, the activity similarity can be measured with the expression

$$S_{i,j} = 1 - \frac{|A_i - A_j|}{\max - \min} \qquad (1)$$

where $A_i$ and $A_j$ are the activities of the *i*th and *j*th molecules ($pIC_{50}$) values, and max − min indicates the range of activities in the data set.

The activity of molecules **45-48** is available as categorical values (>10,000). For the exploratory nature of the SAR of this study these values were approximated as exact values.

**Activity Landscape.** For each pair of compounds the potency difference was plotted against their structural similarity. A general form of the plot is presented in Figure 1. In this plot the potency (absolute) difference is represented in the Y-axis, and the structural similarity is plotted in the X-axis. The graph is reminiscent of the neighborhood plots[30] and the like that have been reported for different enzyme inhibitors using MACCS keys.[31] Related plots have been published for different analyses.[32,33] Additionally, graphs relating structure similarity (e.g., as defined in eq 1) vs structural similarity, called Structure−Activity Similarity (SAS) maps, have been described.[34]

Four zones can be distinguished in Figure 1, labeled as regions I−IV. Data points that fall into region I correspond to pairs of molecules with low structural-similarity and low potency difference and therefore are associated with regions of scaffold hopping. Points plotted in region II denote pairs of molecules with high structural similarity and low potency difference. Thus, compounds in this region are in a smooth or continuous SAR landscape (*vide supra*). Region III identifies pairs of molecules that have high structural similarity and high potency difference and therefore correspond to activity cliffs or discontinuous SAR. Data points in region IV are perhaps the least informative pairs in the plot since this region denotes pairs of molecules with low structural similarity and high potency difference.

In order to characterize the plots obtained with different similarity measures, each plot was partitioned by imposing potency-difference and structural similarity thresholds along the Y- and X-axis, respectively, and then counting the number of data pairs in the resultant regions I−IV (Figure 1). In this study, a potency difference threshold of one log unit was chosen. For similarity, the median similarity of the most active compounds in the data set ($IC_{50} \leq 500$ nM; compounds **1-13**) was considered. Since the different molecular representations lead to different ranges of similarity values for the same set of compounds, the threshold for the structural similarity depends on the representation used, hence normalizing to the median similarity of a descriptor allows us the ability to compare between different descriptor sets.

To further compare the plots obtained from different structural similarity methods the number of data points consistently put into the same region was computed for a given combination of representations. The following Tanimoto-like expression was adopted to measure the *Degree of Consensus* (DoC) between two methods for each region *R*

$$\mathrm{DoC}^{R}_{m,n} = \frac{\mathrm{Cp}_{m,n}}{p_m + p_n - \mathrm{Cp}_{m,n}} \qquad (2)$$

where $\mathrm{Cp}_{m,n}$ is the number of *Consensus Pairs* in region *R* (R = I−IV in Figure 1) between methods *m* and *n*, $p_m$ is the number of pairs of molecules assigned by method *m* in region *R*, and $p_n$ is the number of pairs of molecules assigned by method *n* in the same region. Thus DoC represents the "similarity" that two methods exhibit for assigning molecules to a given region in Figure 1. Note that DoC depends on the thresholds used to define regions I−IV. Results were summarized in the corresponding DoC matrix for a given region. The Doc matrix for region III was subjected to hierarchical clustering using single linkage and principal component analysis (PCA) in MatLab 7.4.0.287[35] and Spotfire 9.1.1,[36] respectively.

**Activity Cliffs with SALI.** The presence of activity cliffs was also evaluated by computing the SALI values for different structural representations. The SALI parameter is calculated with the expression[6,18]

$$\mathrm{SALI}_{i,j} = \frac{|A_i - A_j|}{1 - \mathrm{sim}(i,j)} \qquad (3)$$

where $A_i$ and $A_j$ are the activities of the *i*th and *j*th molecules, and sim(*i*,*j*) is the similarity coefficient between the two molecules.

## RESULTS AND DISCUSSION

**Distribution of Similarity Measures.** The 1128 pairwise similarities of the 48 BCGs in Table 1 calculated with the 11 molecular representations are summarized in Figure 2 as a corresponding cumulative distribution function (CDF). The table at the bottom of the figure summarizes the statistics of the curves indicating the corresponding maximum, third and first quartiles, median, average, and standard deviation. It can be concluded from the CDF that the TGD and TGT fingerprints showed the lowest resolution for this data set.

The high median similarity of MACCS keys (0.80) is partially a function of the relatively low structural diversity of this set which is not surprising since all of the molecules share a common template, the bicycle guanidine ring (Table 1). Additionally, the high median similarity obtained from MACCS keys might also be attributed to the low resolution of the 166 keys considered in this study. Similar observations of the low resolution of the MACCS keys 166-bits for other data sets have been reported.[6] However, it should be noted that the MACCS keys were not developed for similarity

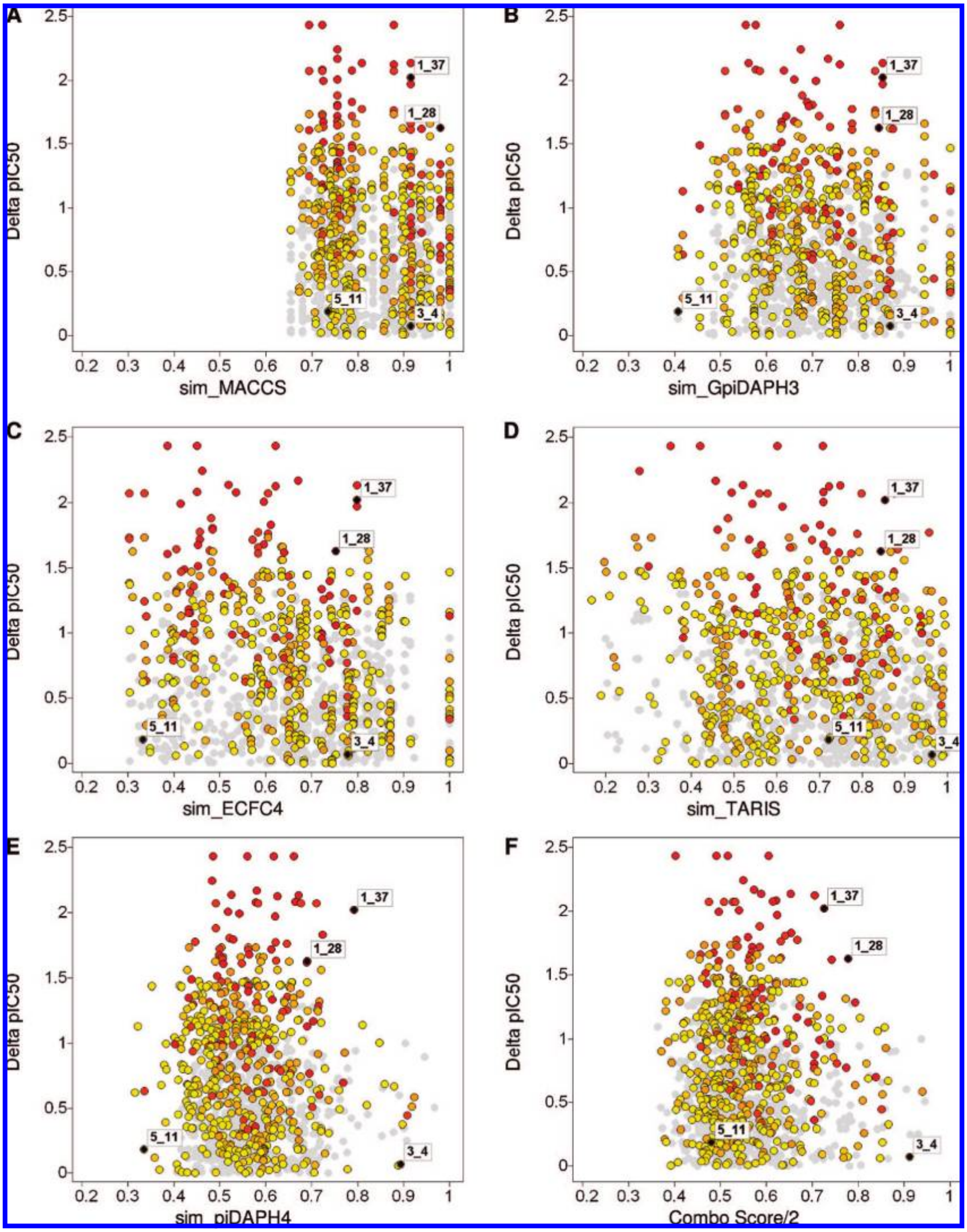


**Figure 5.** Absolute potency difference vs structural similarity with different structural representations. 533 points with at least one active compound ($IC_{50} \leq 500$ nM) in the pair are color-coded by the activity of the most active compound in the pair using a continuous scale from orange (less active) to red (most active). The remaining pairs are displayed in light gray. Four selected pairs are marked in black and labeled with the compound numbers. See also Figure 4.

analysis but for speeding up substructure searching in databases, which due to the subgraph isomorphism problem can be computationally very demanding. By assigning each topological arrangement of atoms a set of key bits, a search for a particular substructure can then be sped up by screening out molecules which do *not* set this particular set of key bits. Hence, structural keys such as MACCS keys are often more in line with chemical intuition; on the other hand they sometimes lack the versatility encountered by fingerprints, which possess much larger descriptor sets.[37]

Other 2D fingerprints employed in this study were the graph based three point pharmacophore GpiDAPH3 and circular fingerprints ECFC4 and ECFP4 which all showed lower median values of 0.69, 0.66, and 0.45, respectively. Circular fingerprints ECFC4 and ECFP4 showed a wide range of similarities from a maximum of 1.0 to a minimum similarity of 0.3 and 0.2, respectively, and the highest standard deviation (>0.15) for any of the 2D fingerprints considered in the study. This is in agreement with the large number of possible features that can be constructed from

**Table 2.** Distribution of Data Points (Pairs) in Different Regions in the Potency Difference vs Structural Similarity Plots[a]

| representation | median similarity of actives[b] | I | | II | | III | | IV | |
|---|---|---|---|---|---|---|---|---|---|
| | | total[c] | active pairs[d] | total | active pairs | total | active pairs | total | active pairs |
| MACCS | 0.92 | 665 | 224 | 191 | 85 | 48 | 38 | 224 | 186 |
| GpiDAPH3 | 0.75 | 541 | 194 | 315 | 115 | 95 | 77 | 177 | 147 |
| ECFC4 | 0.72 | 511 | 179 | 345 | 130 | 80 | 66 | 192 | 158 |
| ECFP4 | 0.58 | 579 | 195 | 277 | 114 | 74 | 59 | 198 | 165 |
| piDAPH3 | 0.75 | 449 | 183 | 407 | 126 | 113 | 89 | 159 | 135 |
| piDAPH4 | 0.58 | 515 | 187 | 341 | 122 | 96 | 80 | 176 | 144 |
| Shape Tanimoto | 0.57 | 538 | 186 | 318 | 123 | 98 | 85 | 174 | 139 |
| Combo Score/2 | 0.56 | 487 | 180 | 369 | 129 | 107 | 95 | 165 | 129 |
| TARIS | 0.67 | 357 | 156 | 499 | 153 | 128 | 102 | 144 | 122 |

[a] Regions I−IV are defined by the median similarity of compounds **1-13** and 1 log unit difference in the activity (see also Figure 1 and text for details). Pie charts showing distributions for selected representations are depicted in Figure 6. [b] Median similarity of compounds **1-13** (compounds with $IC_{50} \leq 500$ nM). [c] Total number of data points (pair of compounds) in the region. [d] Number of data points with at least one compound in the pair with $IC_{50} \leq 500$ nM.

circular fingerprints which give different features if only a single atom within the fingerprint radius is of a different type—which happens rather frequently.

The 3D methods, Shape Tanimoto, Combo Score, and the four-point pharmacophore piDAPH4, all have a nearly identical distribution with a median of approximately 0.5 and standard deviation of 0.1 (Figure 2). These three methods showed approximately normal distributions (as can be deduced from the sigmoidal shape of the corresponding CDF). The spatial three-point pharmacophore piDAPH3, another 3D fingerprint, has a nearly normal distribution and a lower resolution than Shape Tanimoto, Combo Score, and piDAPH4 (median of 0.74 as compared to 0.55). Noteworthy, similarity values obtained with TARIS showed a different distribution than the other 3D methods. Furthermore, TARIS showed the largest standard deviation of all the 2D and 3D methods considered in the study (Figure 2). This observation further supports the use of TARIS to identify consensus data pairs, in particular consensus activity cliffs (*vide infra*), at least for the current data set. This is because ideally consensus activity cliffs will be identified using as many orthogonal descriptions as possible.

**Correlation between 2D and 3D Similarities.** The correlation between 2D and 3D similarity methods for the 1128 pairwise similarities is shown in Figure 3. The correlation matrix shows the relationships between the 11 methods. The two-dimensional plots depict selected 2D vs 3D relationships. From the matrix the high correlation between 2D methods such as TGD and TGT (correlation of 0.94), MACCS keys and TGD/TGT; ECFC4 and ECFP4 (correlation ≥0.84) is readily observed. High correlations between 3D methods occur for Shape Tanimoto and Combo Score (0.82). This is not surprising since Combo Score is the average of Shape Tanimoto and Color Score as described in the Methods section. When comparing the correlation between 2D vs 3D methods the highest correlation observed is between GpiDAPH3 and piDAPH3 (0.79). The largest correlation between unrelated 3D fingerprints is between ECFP4 and piDAPH4 (with a value of 0.61, also depicted in Figure 3D). Other 2D vs 3D correlations are relatively low, for example between MACCS keys and Combo Score (0.38; Figure 3A); between MACCS keys and piDAPH4 (0.53; Figure 3B); and between ECFP4 and Combo Score (correlation of 0.34, Figure 3C). Note that data points in Figure 3A,B are shifted to high similarity values for MACCS keys as expected from the CDF for MACCS keys in Figure 2.

In Figure 3A−D a significant number of data points (again each data point represents a pair of compounds) have a similarity value of 1 along the X-axis (2D similarity). These pairs of compounds mainly correspond to the pairing of 36 stereoisomers present in the data set (Table 1) which cannot be distinguished using these 2D fingerprints. However, as illustrated in Figure 3A−D, Figure 3D similarity provides additional information separating the pairs of stereoisomers along the Y-axis. Interestingly, Figure 3A−D shows several additional pairs of compounds with the same 2D similarity value, less than the unity, but with different 3D similarity values. This is significant in Figure 3A,B and can be explained, at least in part, by the low resolution of MACCS keys. MACCS keys, as compared with ECFP4 fingerprints in Figure 3C,D, have a significantly higher number of points with similarity of 1.0 (72 points total) (*vide infra*).

Despite the several low inter- and intracorrelations for the 2D and 3D methods considered in this study it is possible to identify data pairs with either high or low similarity for most of the molecular representations (*vide infra*).

**Activity Landscape.** *Potency Difference vs Structural Similarity Plots.* Figure 4 depicts scatter plots showing the relationship between potency difference and a number of selected 2D and 3D similarities (see the general form of the plot in Figure 1). Each plot contains 1128 data points that represent a pairwise comparison. Data points are further distinguished by the activity of the most active compound in the pair in a continuous scale from green (less active; $pIC_{50} = 5$) to red (most active, $pIC_{50} = 7.43$). Since the most interesting regions in the landscape are those involving active compounds, it is possible to generate a visual representation of the plots by coloring data points where at least one compound is active (e.g., orange-to-red points). Thus, Figure 5 depicts in color 533 active pairs where at least one compound in the pair is active (in this case the threshold to define an active compound was $IC_{50} \leq 500$ nM but other thresholds for activity could be applied). Inactive pairs are displayed in light gray for reference. Noteworthy, red points are distributed along the entire range of potency difference (~2.5 log units) (Figure 5). Red points at the top of the plot (high potency difference values) contain one active compound and one inactive. In contrast, the red points at the

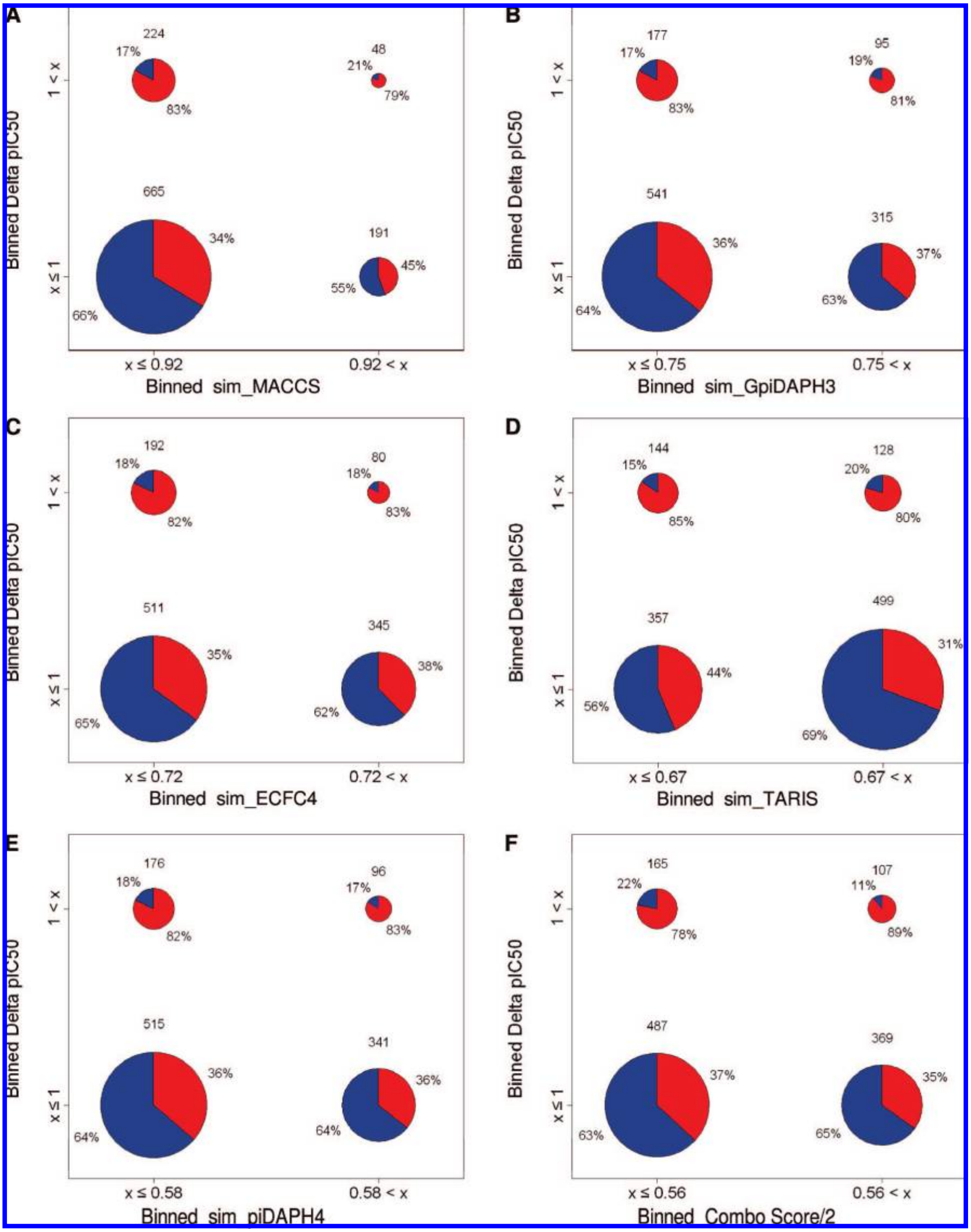


**Figure 6.** Distribution of data points in binned regions of potency difference vs structural similarity plots obtained with different molecular representations: (A) MACCS keys; (B) GpiDAPH3; (C) ECFC4; (D) TARIS; (E) piDAPH4; and (F) Combo Score (scaled). Pie charts indicate the number of data points (pair of compounds) in the four different regions as defined in Figure 1. Red slices represent the number of pairs with at least one active in the pair ($IC_{50} \leq 500$ nM).

bottom of the plot (low potency difference) denote a pair of compounds where both are active.

As expected from the CDF in Figure 2, similarity values obtained with MACCS keys are shifted to towards higher similarity values (>0.65) (Figures 4A/5A), while the similarity values for GpiDAPH3, ECFC4, TARIS, piDAPH4, and Combo Score are more evenly spread (Figures 4B/5B−4F/5F), particularly for the ECFC4 and TARIS plots (cf. Figure 1). Similar observations are made for the plots generated with the other 2D and 3D molecular representations (not shown). In Figure 4A/5A roughly two clusters can be distinguished at MACCS keys similarity of ~0.83. The same behavior is observed in the corresponding CDF in Figure 2, and this trend is attributed to the presence or absence of the methoxybenzyl group at $R_2$ (vide supra). Noteworthy in Figures 4A/5A−4C/5C is the number of pairs with 2D similarity of 1.0 (72 pairs for MACCS keys and 36 pairs for GpiDAPH3 and ECFC4). A number of these points are pairs of compounds that cannot be distinguished by the low resolution of MACCS keys.

**Table 3.** Examples of Pairs of Compounds in Different Regions of the Potency Difference vs Structural Similarity Plots[a]

| region | pair | Delta $pIC_{50}$ | MACCS | GpiDAPH3 | ECFC4 | ECFP4 | piDAPH4 | Combo Score/2 | TARIS |
|---|---|---|---|---|---|---|---|---|---|
| I | **3_9** | 0.29 | 0.76 | 0.69 | 0.56 | 0.49 | 0.50 | 0.41 | 0.47 |
| | **5_11** | 0.19 | 0.74 | 0.41 | 0.33 | 0.22 | 0.34 | 0.48 | 0.72 |
| | **7_9** | 0.03 | 0.78 | 0.61 | 0.68 | 0.37 | 0.41 | 0.49 | 0.49 |
| | **8_9** | 0.03 | 0.72 | 0.74 | 0.65 | 0.43 | 0.56 | 0.58 | 0.59 |
| | **8_11** | 0.03 | 0.75 | 0.53 | 0.61 | 0.38 | 0.37 | 0.41 | 0.59 |
| | **9_10** | 0.004 | 0.78 | 0.61 | 0.68 | 0.37 | 0.45 | 0.49 | 0.44 |
| | **9_13** | 0.07 | 0.74 | 0.57 | 0.45 | 0.23 | 0.41 | 0.55 | 0.57 |
| II | **3_4** | 0.07 | 0.92 | 0.87 | 0.78 | 0.78 | 0.89 | 0.91 | 0.96 |
| | **6_8** | 0.09 | 0.96 | 0.94 | 0.82 | 0.69 | 0.69 | 0.72 | 0.70 |
| | **6_10** | 0.12 | 0.94 | 0.75 | 0.87 | 0.63 | 0.64 | 0.77 | 0.51 |
| | **7_10** | 0.03 | 1.0 | 1.0 | 1.0 | 1.0 | 0.78 | 0.66 | 0.93 |
| III | **1_14** | 1.13 | 1.0 | 1.0 | 1.0 | 1.0 | 0.69 | 0.69 | 0.94 |
| | **1_27** | 1.62 | 0.94 | 0.88 | 0.64 | 0.62 | 0.69 | 0.74 | 0.75 |
| | **1_28** | 1.63 | 0.98 | 0.84 | 0.75 | 0.66 | 0.69 | 0.78 | 0.84 |
| | **1_37** | 2.02 | 0.92 | 0.85 | 0.80 | 0.79 | 0.79 | 0.73 | 0.85 |
| | **1_41** | 2.13 | 0.92 | 0.85 | 0.80 | 0.79 | 0.67 | 0.65 | 0.75 |
| | **4_37** | 1.25 | 1.0 | 0.82 | 0.87 | 0.71 | 0.71 | 0.58 | 0.99 |
| | **5_40** | 1.32 | 0.90 | 0.87 | 0.82 | 0.74 | 0.64 | 0.77 | 0.80 |
| | **7_37** | 1.06 | 0.94 | 0.81 | 0.85 | 0.71 | 0.64 | 0.84 | 0.99 |
| | **8_46** | 1.47 | 1.0 | 1.0 | 1.0 | 1.0 | 0.69 | 0.61 | 0.64 |
| | **11_42** | 1.14 | 0.97 | 0.73 | 0.85 | 0.83 | 0.81 | 0.58 | 0.99 |

[a] See Figure 1 for the definition of the regions.

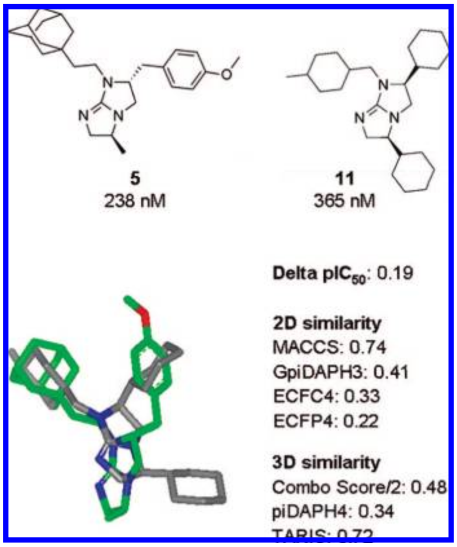


**Figure 7.** Example of a pair of compounds (**5** and **11**) with low 2D and 3D similarity and low potency difference (see also Figure 5). Note the different types of substituents at $R_1$, $R_2$, and $R_3$ around the core template. Particularly, note the absence of heteroatoms from all three substitutents of **11** in contrast to the methoxybenzyl group in **5.** This contributes to the relative low similarity of the molecules. The figure displays the 3D superposition obtained with ROCS.

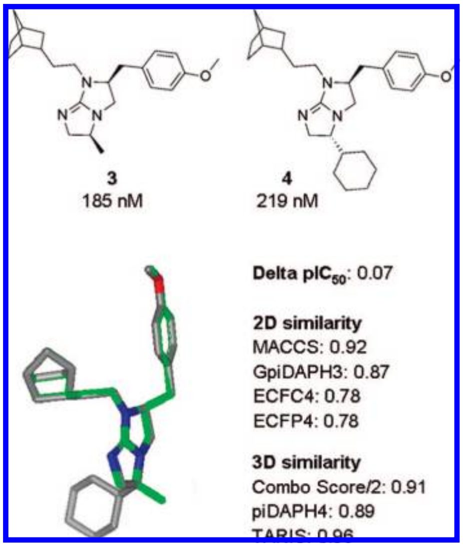


**Figure 8.** Example of a pair of compounds (**3** and **4**) with high 2D and 3D structural similarity and low potency difference (continuous SAR). See also Figure 5. Noteworthy, both molecules have an *S*-4-methoxybenzyl group at $R_2$ and 2-norbornylethyl at $R_3$. The figure displays the 3D superposition obtained with ROCS.

As described in the Methods section, the four regions of potency difference vs structural similarity (I−IV in Figure 1) can also be identified in Figures 4 and 5. Data pairs in regions I and II (cf. Figure 1) are located in a continuous SAR, while pairs of molecules in region III represent activity cliffs. From the discussion above for the different distributions of structural similarities, it follows that the boundary between regions I/IV and II/III, defined by a threshold on the structural similarity (Figure 1), will depend on the molecular representation used. However, despite the different distributions of pairwise similarities for the 2D and 3D methods employed in this work (Figures 2, 4, and 5) and the low correlations between several fingerprint and other representations (Figure 3), it is possible to detect pairs of compounds that are located in the same relative region of each plot. Figure 5 shows some examples of pairs in region I (compounds **5** and **11**) for the six representations (2D and 3D)—region II (**3** and **4**) and region III (activity cliffs **1** and **28**, **1** and **37**). In the next section further details of the quantitative analysis of the potency difference vs structural similarity plots and degree of consensus among different methods is presented.

*Quantitative Characterization of Potency Difference vs Structural Similarity Plots.* In order to carry out a systematic and quantitative analysis of the data obtained in this study, the potency difference vs structural similarity plots were divided into four quadrants (i.e., regions I−IV in Figure 1) by defining thresholds for potency difference (1 log unit) and similarity. The threshold for similarity was set as the median similarity of the actives (e.g., compounds **1-13**) as

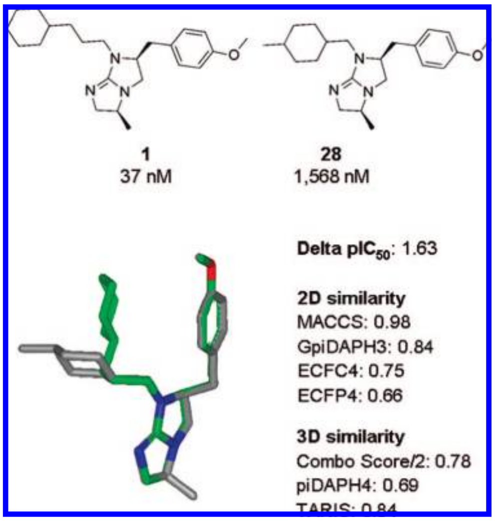


**Figure 9.** Example of a pair of compounds (**1** and **28**) with high potency difference and high structural similarity for a number of molecular representations (consensus activity cliff). See also Figure 5. Notice that both molecules have an *S*-methyl group at $R_1$ and an *S*-4-methoxybenzyl group at $R_2$. The figure displays the 3D superposition obtained with ROCS.

described in the Methods section. Table 2 indicates the median similarity of the active compounds and the number of data pairs that can be found in regions I−IV for 9 out of the 11 structural representations considered in this study. For this analysis the plots generated with TGD and TGT were not considered due to the very low resolution of these fingerprints (Figure 2). Table 2 also includes the number of pairs with at least one active molecule (from now on referred to as *active pairs*; e.g., orange-red points in Figures 4 and 5). Note the variation of the median similarity of the actives with a very high value of 0.92 for MACCS keys and low values, between 0.56 and 0.58, for ECFP4, piDAPH4, Shape Tanimoto, and Combo Score. In fact, a good correlation is found between the median similarities of the actives (**1-13**) (Table 2) with the median similarity of the 48 compounds (Figure 1). This suggests that the diversity of the active compounds is similar to the diversity of the complete data set.

Pie charts illustrating potency difference vs structural similarity are shown in Figure 6. These charts can be regarded as visual representations of the data in Table 2. The size of each pie chart indicates the number of pairs in that particular region. The red slice indicates the fraction of active pairs (i.e., pairs of compounds with at least one molecule with an $IC_{50} \leq 500$ nM) in the corresponding region. For example, Figure 6A indicates that there are 48 activity cliffs (region III in Figure 1), and 79% of the pairs (38 pairs) have at least one molecule with an $IC_{50} \leq 500$ nM.

From Table 2 and Figure 6 it can be concluded that for eight molecular representations most of the pairs of compounds are in region I. Following the criteria to define the thresholds for activity similarity and potency difference (*vide supra*) this is interpreted as follows. For most of the molecular representations the majority of the pairs of molecules have a median structural similarity equal to or less than the median structural similarity of the actives in the data set and a potency difference equal to or less than one log unit. TARIS shows a slightly different behavior with the largest number of pairs residing in region II instead of region I. In contrast, region III (activity cliffs or discontinuous SAR) shows the smallest number of pairs of compounds for all representations. This observation applies not only for the total number of compounds but also for the active pairs (Table 2).

Interestingly, MACCS keys assign the lowest number of pairs to region III. However, this could be a consequence of the low resolution of the molecular representation and the criteria used here to define activity cliffs. In fact, the median similarity of actives using MACCS keys is very high (0.92; Table 2). Since the threshold for structural similarity is set to the median of actives, there is the possibility that in this case MACCS keys hide the activity cliffs. If there are real cliffs we want to identify them. We will further investigate MACCS keys behavior in other data sets with the current approach. These observations further support the importance of considering several representations.

The categorical potency difference vs structural similarity plots analyzed in Table 2 and visualized in Figure 6 are the basis to address a key question. Are there pairs of compounds that are put in the same region by different methods? In other words, are there *consensus pairs*?

*Consensus Activity Cliffs.* Despite the mid-to-low correlations among the results of 2D and 3D molecular representations (Figure 3) and the different distributions of pairwise similarity values (Figure 2) that lead to variations in the appearance of potency difference vs structural similarity plots (Figures 4 and 5), it is possible to find a number of consensus pairs in the plots characterized and visualized in Table 2 and Figure 6, respectively. Table 3 lists several examples of consensus pairs in the three most informative regions (I−III) of the plot. Table 3 also lists the potency difference and the corresponding similarity values for a number of 2D and 3D methods. Note that the 7 selected methods in the table show, in general, a low degree of correlation (<0.8; Figure 3) with the exception of ECFC4 and ECFP4.

A side-by-side comparison of the chemical structures for selected pairs along with the potency difference and 2D and 3D similarity measures is illustrated in Figures 7−9. Figure 7 depicts the consensus pair **5_11** (compound **5** and **11**) from region I. The position of this pair in the corresponding potency difference vs similarity plots for a number of methods is indicated in Figure 5. Noteworthy, substituents at the $R_1$, $R_2$, and $R_3$ positions of **5** and **11** are different leading to relatively low 2D and 3D similarities. However, both compounds have very similar activity (238 and 365 nM, respectively). As discussed above, this is an example associated with scaffold hopping. Although both molecules were obtained from a combinatorial library and the term "scaffold hopping" may not apply fully in this case, the *consensus* low structural similarity values (specially the circular fingerprints) do suggest a "jump" in the activity landscape. This is in agreement with previous studies conducted in our group where either the presence or absence of a methoxybenzyl group at $R_2$ may lead to different modes of binding for active compounds.[27]

Figure 8 illustrates the pair **3_4** (compounds **3** and **4**) as an example of continuous SAR in region II. The position of the pair in the corresponding potency difference vs similarity plots for a number of methods is indicated in Figure 5. Both molecules are active and have similar $IC_{50}$ (185 and 219 nM,

**Region IV**

| | MACCS | GpiDAPH3 | ECFC4 | ECFP4 | piDAPH4 | Combo Score/2 | TARIS |
|---|---|---|---|---|---|---|---|
| MACCS | 224 | | | | | | |
| GpiDAPH3 | 169 | 177 | | | | | |
| ECFC4 | 191 | 160 | 192 | | | | |
| ECFP4 | 195 | 167 | 186 | 198 | | | |
| piDAPH4 | 159 | 139 | 147 | 149 | 176 | | |
| ComboScore/2 | 144 | 117 | 128 | 132 | 132 | 165 | |
| TARIS | 126 | 103 | 111 | 111 | 105 | 105 | 144 |

**Region III**

| | MACCS | GpiDAPH3 | ECFC4 | ECFP4 | piDAPH4 | Combo Score/2 | TARIS |
|---|---|---|---|---|---|---|---|
| MACCS | 48 | | | | | | |
| GpiDAPH3 | 40 | 95 | | | | | |
| ECFC4 | 47 | 63 | 80 | | | | |
| ECFP4 | 45 | 64 | 68 | 74 | | | |
| piDAPH4 | 31 | 58 | 51 | 47 | 96 | | |
| ComboScore/2 | 27 | 47 | 43 | 41 | 63 | 107 | |
| TARIS | 30 | 54 | 47 | 41 | 57 | 68 | 128 |

**Region I**

| | MACCS | GpiDAPH3 | ECFC4 | ECFP4 | piDAPH4 | Combo Score/2 | TARIS |
|---|---|---|---|---|---|---|---|
| MACCS | 665 | | | | | | |
| GpiDAPH3 | 502 | 541 | | | | | |
| ECFC4 | 489 | 422 | 511 | | | | |
| ECFP4 | 541 | 485 | 493 | 579 | | | |
| piDAPH4 | 471 | 425 | 394 | 436 | 515 | | |
| ComboScore/2 | 407 | 360 | 338 | 367 | 374 | 487 | |
| TARIS | 318 | 280 | 257 | 267 | 263 | 239 | 357 |

**Region II**

| | MACCS | GpiDAPH3 | ECFC4 | ECFP4 | piDAPH4 | Combo Score/2 | TARIS |
|---|---|---|---|---|---|---|---|
| MACCS | 191 | | | | | | |
| GpiDAPH3 | 152 | 315 | | | | | |
| ECFC4 | 169 | 226 | 345 | | | | |
| ECFP4 | 153 | 221 | 259 | 277 | | | |
| piDAPH4 | 147 | 225 | 224 | 198 | 341 | | |
| ComboScore/2 | 111 | 188 | 196 | 157 | 228 | 369 | |
| TARIS | 152 | 238 | 245 | 187 | 247 | 251 | 499 |

**Figure 10.** Number of consensus pairs between 2D and 3D molecular representations in the four regions of the potency difference vs structural similarity plots (Figure 1). Numbers along the diagonal indicate the number of pairs that a given method places in a particular region.

**Region IV**

| | MACCS | GpiDAPH3 | ECFC4 | ECFP4 | piDAPH4 | Combo Score/2 | TARIS |
|---|---|---|---|---|---|---|---|
| MACCS | 1.00 | | | | | | |
| GpiDAPH3 | 0.73 | 1.00 | | | | | |
| ECFC4 | 0.85 | 0.77 | 1.00 | | | | |
| ECFP4 | 0.86 | 0.80 | 0.91 | 1.00 | | | |
| piDAPH4 | 0.66 | 0.65 | 0.67 | 0.66 | 1.00 | | |
| ComboScore/2 | 0.59 | 0.52 | 0.56 | 0.57 | 0.63 | 1.00 | |
| TARIS | 0.52 | 0.47 | 0.49 | 0.48 | 0.49 | 0.51 | 1.00 |

Median = 0.63/ Mean = 0.64 / Stdev = 0.14

**Region III**

| | MACCS | GpiDAPH3 | ECFC4 | ECFP4 | piDAPH4 | Combo Score/2 | TARIS |
|---|---|---|---|---|---|---|---|
| MACCS | 1.00 | | | | | | |
| GpiDAPH3 | 0.39 | 1.00 | | | | | |
| ECFC4 | 0.58 | 0.56 | 1.00 | | | | |
| ECFP4 | 0.58 | 0.61 | 0.79 | 1.00 | | | |
| piDAPH4 | 0.27 | 0.44 | 0.41 | 0.38 | 1.00 | | |
| ComboScore/2 | 0.21 | 0.30 | 0.30 | 0.29 | 0.45 | 1.00 | |
| TARIS | 0.21 | 0.32 | 0.29 | 0.25 | 0.34 | 0.41 | 1.00 |

Median = 0.38 / Mean = 0.40 / Stdev = 0.15

**Region I**

| | MACCS | GpiDAPH3 | ECFC4 | ECFP4 | piDAPH4 | Combo Score/2 | TARIS |
|---|---|---|---|---|---|---|---|
| MACCS | 1.00 | | | | | | |
| GpiDAPH3 | 0.71 | 1.00 | | | | | |
| ECFC4 | 0.71 | 0.67 | 1.00 | | | | |
| ECFP4 | 0.77 | 0.76 | 0.83 | 1.00 | | | |
| piDAPH4 | 0.66 | 0.67 | 0.62 | 0.66 | 1.00 | | |
| ComboScore/2 | 0.55 | 0.54 | 0.51 | 0.53 | 0.60 | 1.00 | |
| TARIS | 0.45 | 0.45 | 0.42 | 0.40 | 0.43 | 0.40 | 1.00 |

Median = 0.60 / Mean = 0.59 / Stdev = 0.13

**Region II**

| | MACCS | GpiDAPH3 | ECFC4 | ECFP4 | piDAPH4 | Combo Score/2 | TARIS |
|---|---|---|---|---|---|---|---|
| MACCS | 1.00 | | | | | | |
| GpiDAPH3 | 0.43 | 1.00 | | | | | |
| ECFC4 | 0.46 | 0.52 | 1.00 | | | | |
| ECFP4 | 0.49 | 0.60 | 0.71 | 1.00 | | | |
| piDAPH4 | 0.38 | 0.52 | 0.48 | 0.47 | 1.00 | | |
| ComboScore/2 | 0.25 | 0.38 | 0.38 | 0.32 | 0.47 | 1.00 | |
| TARIS | 0.28 | 0.41 | 0.41 | 0.32 | 0.42 | 0.41 | 1.00 |

Median = 0.42 / Mean = 0.43 / Stdev = 0.11

**Figure 11.** Degree of consensus (DoC) matrix for each region. Each entry in the corresponding matrix represents the agreement between two methods to place a pair of compounds into the same region. DoC is computed with eq 2 using data in Figure 10. The median, mean, and standard deviation of each DoC matrix is also included and represents the overall degree of consensus of the methods for a given region.

respectively) values. The only structural difference between **3** and **4** is at $R_1$ (*S*-methyl versus *R*-cyclohexyl, respectively). This results in a high 2D and 3D similarity by a number of methods. Figure 9 depicts an example of a consensus cliff (**1_28**) that is located in region III for different 2D and 3D molecular representations (Table 3 and Figure 5). As can be seen from Figure 9 and Table 1 both molecules have the same substituents and stereochemistry at $R_1$ and $R_2$. Although **1** has a 3-cyclohexylpropyl group at $R_3$ and **28** a 4-(Me)-cyclohexylmethyl group at the same position, both molecules have a high 2D and 3D similarity (higher than the corresponding median similarity of actives in this data set). However, this change in structure produces a decrease in activity from 37 nM to 1568 nM. Therefore, this activity cliff provides information for the steric requirements for activity at $R_3$.

Despite the inherent conformational issues, the use of 3D structural representations is valuable when dealing with pairs of stereoisomers. Table 3 lists two examples of consensus cliffs that involve stereoisomers: Pairs **1_14** and **8_46**. The similarity for 2D representations is 1.0. However, all 3D methods in Table 3 provide additional information pointing to particular cases where changes in the stereochemistry have a large impact on activity. For example, the change of *S*- to *R*-configuration of the methoxybenzyl group of **1**.

As a side note, **4_37** are different molecules ($R_3$ = 2-norbornylethyl and 3-cyclohexylpropyl, respectively). However, according to MACCS keys the similarity is 1.0 (Table 3). Other

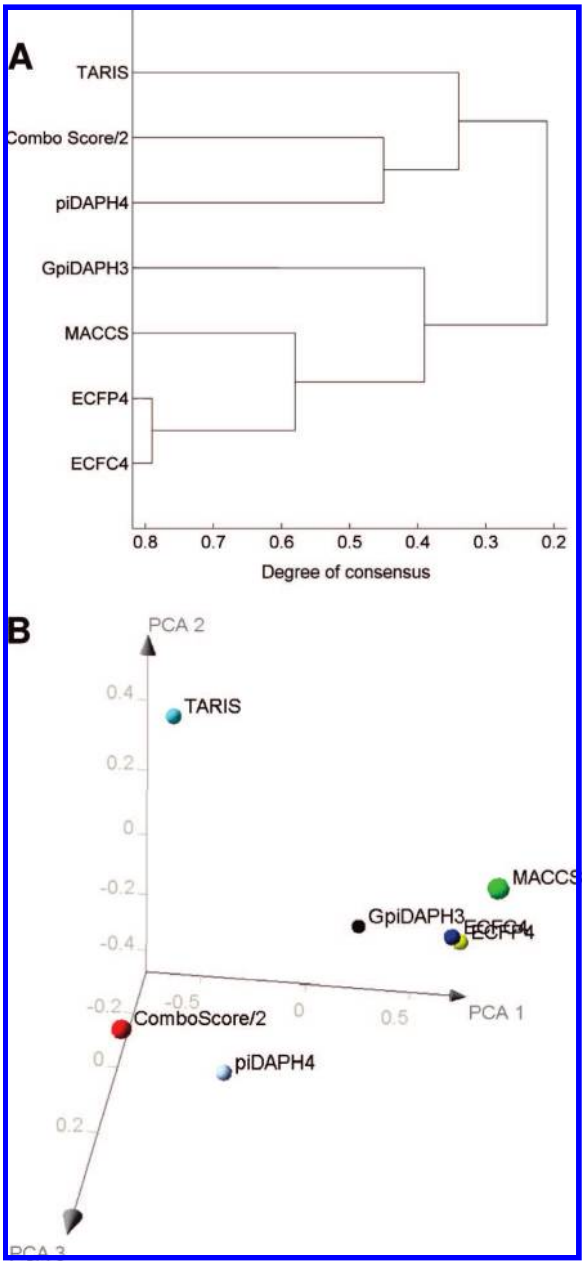


**Figure 12.** Clustering and visualization of DoC matrix in region III (Figure 11): (A) hierarchical clustering (complete linkage) and (B) PCA. The total variance of the first tree PC is 83.5%. Note the overall higher degree of consensus between 2D methods.

2D fingerprints such as GpiDAPH3, ECFC4, and ECFP4 and 3D methods are able to differentiate the structures. This is an example of the low resolution of MACCS keys-166 bits discussed above and again underscores the value in using several molecular representations to derive conclusions regarding structure−activity analyses.[16,23]

The importance of activity cliffs in activity landscapes has been discussed in the literature.[5,7] Activity cliffs are valuable for detecting specific structural changes important for activity. Noteworthy, compound **1**, the most active in the data set, is involved in several consensus activity cliffs detected in this analysis: **1_14**; **1_27**; **1_28**; **1_37**; and **1_41** (Table 3). The cliff **1_14** provides information about the specific stereochemical requirements of the 4-methoxybenzyl group at $R_2$. Although the change in activity for this pair (37 nM vs 502 nM) is not as dramatic as the cliff from **1** and **37** (37 nM vs 3872 nM) it reveals the importance of a small substituent at $R_1$ (*S*-methyl vs *S*-cyclohexyl). The consensus cliff **1_41** confirms further the detrimental effects in the activity if both changes occur. Thus, **41** has an *S*-cyclohexyl at $R_1$ (same as **37**) and an *R*-4-methoxybenzyl at $R_2$ (same as **14**) and is less active (5026 nM) than **14** and **37** (502 and 3872 nM, respectively). Note that **1**, **14**, **37**, and **41** have the same substituent (3-cyclohexylpropyl) at $R_3$.

**Table 4.** Distribution of SALI (and Normalized Values) for Different 2D and 3D Representations

| SALI/ Representation | max | Q3[a] | median | Q1[b] | min | mean | STD |
|---|---|---|---|---|---|---|---|
| MACCS | 77.48 | 7.27 | 3.57 | 1.72 | 0.00 | 10.01 | 18.99 |
| GpiDAPH3 | 34.89 | 3.57 | 2.08 | 1.00 | 0.00 | 3.78 | 6.50 |
| ECFC4 | 14.85 | 3.17 | 1.79 | 0.87 | 0.00 | 2.64 | 2.98 |
| ECFP4 | 10.31 | 1.99 | 1.15 | 0.58 | 0.00 | 1.68 | 1.96 |
| piDAPH4 | 16.62 | 2.27 | 1.34 | 0.64 | 0.00 | 1.64 | 1.43 |
| Combo Score/2 | 16.01 | 2.26 | 1.32 | 0.63 | 0.00 | 1.63 | 1.39 |
| TARIS | 182.85 | 4.11 | 2.04 | 0.94 | 0.00 | 4.66 | 11.07 |
| **normalized SALI** | **max** | **Q3[a]** | **median** | **Q1[b]** | **min** | **mean** | **STD** |
| MACCS | 3.55 | −0.14 | −0.34 | −0.44 | −0.53 | 0.00 | 1.00 |
| GpiDAPH3 | 4.78 | −0.03 | −0.26 | −0.43 | −0.58 | 0.00 | 1.00 |
| ECFC4 | 4.09 | 0.18 | −0.28 | −0.59 | −0.88 | 0.00 | 1.00 |
| ECFP4 | 4.40 | 0.16 | −0.27 | −0.56 | −0.86 | 0.00 | 1.00 |
| piDAPH4 | 10.45 | 0.44 | −0.21 | −0.70 | −1.15 | 0.00 | 1.00 |
| Combo Score/2 | 10.32 | 0.46 | −0.22 | −0.71 | −1.17 | 0.00 | 1.00 |
| TARIS | 16.10 | −0.05 | −0.24 | −0.34 | −0.42 | 0.00 | 1.00 |

[a] Third quartile. [b] First quartile.

The consensus cliff associated with **1_27** and **1_28** also provides information on the steric requirements at $R_3$ where the substitution of the 3-cyclohexylgroup in **1** by a 1-adamantylethyl in **27** or by a 4-(Me)-cyclohexylmethyl in **28** decreases the activity from 37 nM in **1** to 1532 and 1568 nM, respectively. These observations suggest that molecule **1** is not a good candidate to be used as a reference (query) in similarity-based virtual screening studies since it is likely that compounds similar to **1** would be much less active.[7] Similarly, these observations anticipate potential pitfalls if common computational methods like QSAR are used to optimize the activity of **1**. Instead, experimental work is currently ongoing in our group to further understand the role of the 4-methoxybenzylgroup of **1**. Previous studies suggest that replacement of the methoxy group with a hydroxyl group may increase the activity.[27]

*Degree of Consensus.* Figure 10 summarizes the number of pairs of compounds that two methods put into the same region i.e., number of consensus pairs. Note that the terms on the diagonal indicate the number of pairs that a given method places into that region. The *degree of consensus* (DoC) between two methods is presented in Figure 11. DoC, which is computed with eq 2 detailed in the Methods section, measures the number of consensus pairs between two methods scaled by the total number of pairs that the two methods put into the same region. Figure 11 also indicates the overall degree of consensus for several 2D and 3D methods in each region. This overall measure is obtained by the median, mean, and standard deviation of each DoC matrix. From Figure 11 it is readily concluded that the overall degree of consensus decreases in the following order: region IV > I > II > III. In other words, there is a better agreement between the methods to assign molecules to region IV

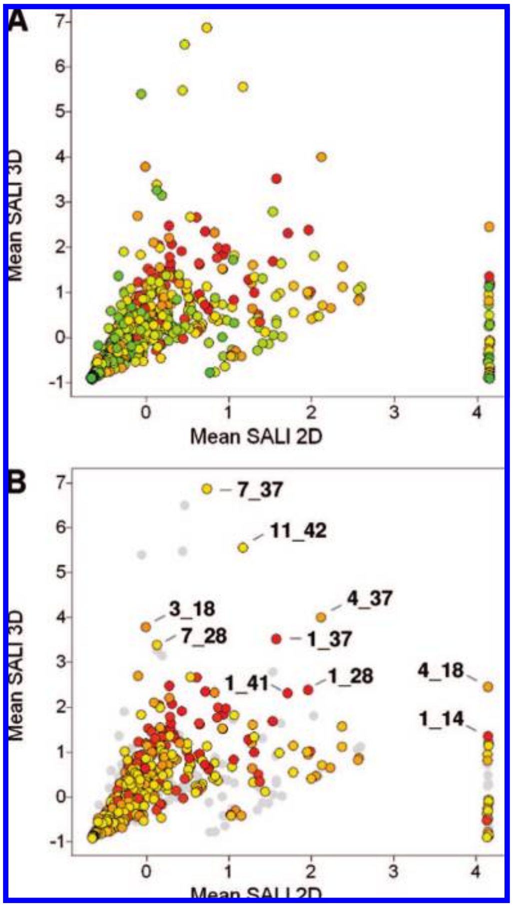


**Figure 13.** Comparison of SALI values computed for 2D and 3D methods. The Y-axis indicates the mean of normalized SALI values computed with piDAPH4, Combo Score, and TARIS (three low-correlated 3D methods). The X-axis indicates the mean of normalized SALI values computed with MACCS, GpiDAPH3, and ECFC4 (three low-correlated 2D methods). (A) All 1128 pairwise comparisons are color coded by the activity of the most active compound in the pair using a continuous scale from green (less active) to red (most active). (B) 533 points with at least one active compound ($IC_{50} \leq 500$ nM) in the pair are color-coded by the activity of the most active compound in the pair using a continuous scale from orange (less active) to red (most active). The remaining pairs are displayed in gray. Ten selected pairs with high SALI values by either 2D or 3D or both are labeled. Specific SALI values for the selected pairs are detailed in Table 5. Data points are jittered.

(overall DoC of 0.63 considering the median) than in any other region. In contrast, the least agreement between methods occurs for region III (overall DoC of 0.38). This means that it is more difficult to identify consensus activity cliffs than, for example, identify compounds in a continuous region of SAR (regions I and II). It should be noted that DoC is dependent on the criteria used to define the four regions. The number of consensus activity cliffs between two methods (Figure 10) and the number of consensus cliffs for several methods (Table 3) reveal the heterogeneous nature of the SAR of the BCGs considered in this study. Heterogeneous SAR have been reported for other compounds targeting different enzymes.[38]

Figure 12 depicts clustering and visualization of the DoC matrix for region III. A similar analysis can be derived for the other regions. Here we focus on region III because of the importance of activity cliffs in the activity landscape.[5] Figure 12A shows the hierarchical clustering using complete linkage, and Figure 12B portrays the PCA (the first three PC accounts for 83.5% of the variance). From these figures the high degree of consensus between ECFC4 and ECFP4 (DoC = 0.79; Figure 11) is readily observed. This is not surprising due to the high correlation observed for this data set between these fingerprints (Figure 3). Similar conclusions for the high degree of consensus between ECFC4 and ECFP4 can be derived for DoC matrices in regions I, II, and IV (Figure 11). In contrast, TARIS shows the least degree of correlation with the other methods not only for region III as observed in Figure 12 but also in other regions (Figure 11). This can be attributed to the very different nature of this method which computes similarity based on the MEP.[28] As the method only considers the negative electrostatic potential, the regions of the molecules that present negative atoms like oxygen and nitrogen are more relevant, and the positive regions can even be ignored. This method was conceived in this way because of the strong correlation between the presence of those atoms and the reactivity of molecules. In general, 2D methods show a higher degree of consensus in region III than 3D methods. For example, MACCS keys, ECFC4, and ECFP4 are closer together in the PCA in Figure 12B. This is in agreement with the clustering observed in Figure 12A.

**Activity Cliffs with SALI.** The SALI parameter (eq 3) has been recently proposed as a metric to easily detect activity cliffs. This index has been used to represent SAR as a graph (SALI networks).[6] The parameter is the basis of the so-called SALI curves that have been developed as an approach for measuring the capability of a given model for detecting activity cliffs.[18] Since the SALI value is dependent on the molecular representation used to compute the similarity in eq 3, the value for a number of 2D and 3D molecular representations was computed and then "fused" by obtaining the corresponding mean of normalized SALI values. The goal was to identify consensus cliffs detected by 2D and 3D methods as presented ahead.

We focused on seven 2D and 3D methods. Five of the seven methods show a low correlation for the current data set (Figure 3), the exceptions being the fingerprints ECFC4 and ECFP4 which show a high correlation to each other (0.84 correlation). For the cases of pairs of molecules with a similarity value of 1.0 (e.g., stereoisomers or cases of low fingerprint resolution like the pair **4_37** in Table 3) the corresponding SALI value was replaced with the largest SALI value in the set. Results are presented in Table 4 showing a summary of the distribution of SALI values and normalized SALI values. Note the negative values of the median, first quartile, etc. for normalized SALI values due to the mean centering. Note also the overall higher normalized SALI values for 3D methods. For example the maximum normalized SALI value for piDAPH4, Combo Score, and TARIS is 10.45, 10.32, and 16.10, respectively, meanwhile the corresponding normalized value for any 2D method is less than 5. This is in agreement with the number of pairs of compounds that the 3D methods put in region III (Figure 10). Similar conclusions can be obtained by comparing the corresponding third quartile (omitting TARIS). As discussed above, the lower number of pairs of compounds placed by 2D methods in region III could be a consequence of the low resolution of the molecular representation.

Figure 13 depicts the relationship between the mean of normalized SALI values computed from 3D and 2D methods.

**Table 5.** Normalized SALI Values for Selected Pairs of Compounds[a]

| | | 2D | | | | 3D | | | |
|---|---|---|---|---|---|---|---|---|---|
| pair | Delta $pIC_{50}$ | MACCS | GpiDAPH3 | ECFC4 | mean | piDAPH4 | Combo Score/2 | TARIS | mean |
| **1_14** | 1.13 | 3.6 (77.5) | 4.8 (34.9) | 4.1 (4.9) | 4.1 | 1.4 (3.7) | 1.5 (3.7) | 1.2 (18.0) | 1.4 |
| **1_28** | 1.63 | 3.6 (77.5) | 1.0 (10.4) | 1.3 (6.6) | 2.0 | 2.5 (5.3) | 4.1 (7.3) | 0.5 (10.4) | 2.4 |
| **1_37** | 2.02 | 0.7 (23.8) | 1.5 (13.7) | 2.5 (10.0) | 1.6 | 5.6 (9.7) | 4.1 (7.4) | 0.8 (13.7) | 3.5 |
| **1_41** | 2.13 | 0.8 (25.1) | 1.7 (14.5) | 2.7 (10.6) | 1.7 | 3.4 (6.5) | 3.3 (6.2) | 0.4 (8.5) | 2.3 |
| **4_18** | 0.51 | 3.6 (77.5) | 4.8 (34.9) | 4.1 (14.8) | 4.1 | 3.2 (6.3) | 0.8 (2.7) | 3.4 (41.7) | 2.5 |
| **4_37** | 1.25 | 3.6 (77.5) | 0.5 (7.0) | 2.3 (9.4) | 2.1 | 1.9 (4.4) | 1.0 (3.0) | 9.2 (106.1) | 4.0 |
| **3_18** | 0.59 | −0.2 (6.9) | 0.1 (4.5) | 0.0 (2.6) | 0.0 | 4.2 (7.6) | 5.7 (9.6) | 1.5 (20.9) | 3.8 |
| **7_28** | 0.67 | 0.3 (15.9) | 0.0 (3.5) | 0.1 (3.0) | 0.1 | 2.7 (5.5) | 2.4 (4.9) | 5.1 (61.0) | 3.4 |
| **7_37** | 1.06 | 0.4 (16.6) | 0.3 (5.7) | 1.5 (7.2) | 0.7 | 0.9 (2.9) | 3.7 (6.7) | 16.1 (182.9) | 6.9 |
| **11_42** | 1.14 | 1.8 (43.9) | 0.1 (4.2) | 1.6 (7.5) | 1.2 | 3.1 (6.0) | 0.8 (2.7) | 12.8 (146.7) | 5.6 |

[a] See Figure 10. Non-normalized values are in parentheses.

The mean was computed considering only three 2D and 3D weakly correlated representations (Figure 3). Thus, the mean of normalized SALI values using MACCS keys, GpiDAPH4, and ECFC4 (maximum correlation of 0.69) was plotted vs the mean of normalized SALI values using piDAPH4, Combo Score, and TARIS (maximum correlation of 0.54). Figure 13A displays all data pairs, and Figure 13B display only the pairs with at least one compound in the active regions (*vide supra*). *Consensus cliffs are data pairs with both high mean SALI value from 2D and 3D representations.*

In Figure 13B representative consensus cliffs identified in this analysis are marked. The corresponding SALI values and normalized values along with the potency difference are detailed in Table 5. Note that a number of cliffs such as the pairs **1_41**, **1_28**, **1_37**, **4_37**, and **11_42** have high mean SALI values computed from 2D and 3D representations. Other cliffs like **3_18**, **7_28**, and **7_37** have high mean SALI values computed from 3D, and the pair **1_14** (stereoisomers) has a high mean SALI value from 2D.

The pairs **1_14**, **1_28**, **1_37**, **1_41**, **4_37**, **7_37**, and **11_42** were also detected as cliffs from the potency difference vs similarity plots (Table 3). As noted above, other consensus cliffs detected easily with mean SALI values were **4_18**, **3_18**, and **7_28** (Table 5). Note, however, that all three pairs have a potency difference lower than 1.0 and therefore cannot be in region III following the threshold used here to divide regions I/II from III/IV (*vide supra*).

A similar fusion strategy using multiple 2D and 3D representations can be implemented in the SAR Index approach proposed recently.[31] The SAR Index is a quantitative measure that captures the 2D structural diversity and potency distribution for a given set of active compounds. The SAR Index was recently applied to characterize and quantify 16 different sets of enzyme inhibitors.[31] The method was reported using only one 2D structural representation (MACCS keys) but could be extended using multiple representations to derive a *consensus SAR Index*.

## CONCLUSIONS

A systematic analysis that characterizes activity landscapes using multiple 2D and 3D molecular representations is presented. The analysis is based on pairwise comparisons of the potency difference and molecular similarity using 11 2D and 3D molecular representations. To simplify the analysis, however, a single low-energy conformation of each molecule was used. The structure−activity relationships are portrayed in potency difference vs structural similarity plots that can be categorized into four regions for further quantitative comparisons. The Degree of Consensus (DoC) is a measure proposed to compare the ability of any two methods to put a pair of compounds in the same region of the activity landscape. The distribution of pairwise similarity values was assessed revealing the low resolution of several representations for this data set such as MACCS keys, TGT and TGD fingerprints. The correlation between 2D and 3D molecular representations was also evaluated. Several representations lead to high correlations, especially between 2D methods or between 3D methods (correlation coefficient greater than 0.8). However the correlation between 2D and 3D methods is rather low (correlations lower than 0.6 in most cases). Despite the weak correlation between several structural representations a number of consensus activity cliffs and other consensus data pairs in different regions of the activity landscape were identified. Overall, 2D methods showed a better degree of consensus than 3D methods. Although the pairwise similarity distributions and mutual correlations were evaluated, the main purpose in this work was not to identify the "best" representation. Instead, the main goal was to identify the set of representations that helps to find *consensus data pairs* and, in particular, *consensus activity cliffs*. Consensus cliffs were also identified by extending the SALI approach by merging the information from six weakly correlated 2D and 3D representations, namely MACCS keys, GpiDAPH3 and ECFC4 (2D methods), and piDAPH4, Combo Score, and TARIS (3D methods).

Overall a heterogeneous SAR was found for the current test case. The presence of several consensus activity cliffs suggests that the set of 48 BCGs studied in this work is not suitable to derive predictive models using computational methods such as traditional QSAR. Furthermore, several cliffs around the most active compound in the data (**1**) indicate that this molecule alone is not an attractive query for similarity-based virtual screening. Consensus data pairs in scaffold-hopping regions of the landscape suggest that the presence or absence of a methoxybenzyl group at $R_2$ may lead to different binding modes of active compounds. The heterogeneous SAR of the set of molecules derived from a combinatorial library with relatively low molecular diversity suggests the importance of evaluating high-density combinatorial libraries that may contain significant activity cliffs. Despite the limited scope of the 3D similarities as employed in this work, deriving a consensus model of the activity

landscape using multiple molecular representations can be extended to other data sets with other biological end points. Application of this approach to in-house and external data sets is currently in progress in our laboratory and will be published in due course.

## ACKNOWLEDGMENT

Helpful discussions and comments of Dr. Gerald M. Maggiora are gratefully acknowledged. This work was supported by the State of Florida, Executive Office of the Governor's Office of Tourism, Trade, and Economic Development, the Top Institute Pharma project: number D1-105 (A.B.) and COLCIENCIAS, project 201010010456 (R.M.). Authors are also grateful to the National Institute on Drug Abuse (DA019620) and to the Multiple Sclerosis National Research Institute for partial funding. We thank OpenEye Scientific Software, Inc. for providing ROCS, OMEGA, and VIDA programs.

## REFERENCES AND NOTES

(1) Ooms, F. Molecular Modeling and Computer Aided Drug Design. Examples of Their Applications in Medicinal Chemistry. *Curr. Med. Chem.* **2000**, *7*, 141–158.
(2) Cronin, M. T. D.; Schultz, T. W. Pitfalls in QSAR. *J. Mol. Struct.: THEOCHEM* **2003**, *622*, 39–51.
(3) Doweyko, A. M. QSAR: Dead or Alive. *J. Comput.-Aided Mol. Des.* **2008**, *22*, 81–89.
(4) Johnson, S. R. The Trouble with QSAR (or How I Learned to Stop Worrying and Embrace Fallacy). *J. Chem. Inf. Model.* **2008**, *48*, 25–26.
(5) Maggiora, G. M. On Outliers and Activity Cliffs-Why QSAR Often Disappoints. *J. Chem. Inf. Model.* **2006**, *46*, 1535–1535.
(6) Guha, R.; VanDrie, J. H. Structure-Activity Landscape Index: Identifying and Quantifying Activity Cliffs. *J. Chem. Inf. Model.* **2008**, *48*, 646–658.
(7) Eckert, H.; Bajorath, J. Molecular Similarity Analysis in Virtual Screening: Foundations, Limitations and Novel Approaches. *Drug Discovery Today* **2007**, *12*, 225–233.
(8) *Concepts and Applications of Molecular Similarity*; Johnson, M. A., Maggiora, G. M., Eds.; John Wiley & Sons: New York, 1990.
(9) Bender, A.; Glen, R. C. Molecular Similarity: A Key Technique in Molecular Informatics. *Org. Biomol. Chem.* **2004**, *2*, 3204–3218.
(10) Schneider, G.; Neidhart, W.; Giller, T.; Schmid, G. Scaffold-Hopping by Topological Pharmacophore Search: A Contribution to Virtual Screening. *Angew. Chem., Int. Ed.* **1999**, *38*, 2894–2896.
(11) Jenkins, J. L.; Glick, M.; Davies, J. W. A 3D Similarity Method for Scaffold Hopping from Known Drugs or Natural Ligands to New Chemotypes. *J. Med. Chem.* **2004**, *47*, 6144–6159.
(12) Renner, S.; Schneider, G. Scaffold-Hopping Potential of Ligand-Based Similarity Concepts. *ChemMedChem* **2006**, *1*, 181–185.
(13) Zhao, H. Scaffold Selection and Scaffold Hopping in Lead Generation: A Medicinal Chemistry Perspective. *Drug Discovery Today* **2007**, *12*, 149–155.
(14) Brown, N.; Jacoby, E. On Scaffolds and Hopping in Medicinal Chemistry. *Mini-Rev. Med. Chem.* **2006**, *6*, 1217–1229.
(15) Medina-Franco, J. L.; Martinez-Mayorga, K.; Giulianotti, M. A.; Houghten, R. A.; Pinilla, C. Visualization of the Chemical Space in Drug Discovery. *Curr. Comput.-Aided Drug Des.* **2008**, *4*, 323–333.
(16) Sheridan, R. P.; Kearsley, S. K. Why Do We Need So Many Chemical Similarity Search Methods. *Drug Discovery Today* **2002**, *7*, 903–911.
(17) Houghten, R. A.; Pinilla, C.; Giulianotti, M. A.; Appel, J. R.; Dooley, C. T.; Nefzi, A.; Ostresh, J. M.; Yu, Y. P.; Maggiora, G. M.; Medina-Franco, J. L.; Brunner, D.; Schneider, J. Strategies for the Use of Mixture-Based Synthetic Combinatorial Libraries: Scaffold Ranking, Direct Testing, in Vivo, and Enhanced Deconvolution by Computational Methods. *J. Comb. Chem.* **2008**, *10*, 3–19.
(18) Guha, R.; Van Drie, J. H. Assessing How Well a Modeling Protocol Captures a Structure-Activity Landscape. *J. Chem. Inf. Model.* **2008**, *48*, 1716–1728.
(19) Houghten, R. A.; Pinilla, C.; Appel, J. R.; Blondelle, S. E.; Dooley, C. T.; Eichler, J.; Nefzi, A.; Ostresh, J. M. Mixture-Based Synthetic Combinatorial Libraries. *J. Med. Chem.* **1999**, *42*, 3743–3778.
(20) Pinilla, C.; Appel, J. R.; Borras, E.; Houghten, R. A. Advances in the Use of Synthetic Combinatorial Chemistry: Mixture-Based Libraries. *Nat. Med.* **2003**, *9*, 118–122.
(21) Or Tanimoto-like coefficients.
(22) *Molecular Operating Environment (MOE), version 2007*; Chemical Computing Group Inc.: Montreal, Quebec, Canada. Available at http://www.chemcomp.com (accessed Dec 1, 2008).
(23) Bender, A.; Jenkins, J. L.; Scheiber, J.; Sukuru, S. C. K.; Glick, M.; Davies, J. W. How Similar Are Similarity Searching Methods? A Principal Component Analysis of Molecular Descriptor Space. *J. Chem. Inf. Model.* **2009**, *49*, 108–119.
(24) Glen, R. C.; Bender, A.; Arnby, C. H.; Carlsson, L.; Boyer, S.; Smith, J. Circular Fingerprints: Flexible Molecular Descriptors with Applications from Physical Chemistry to ADME. *IDrugs* **2006**, *9*, 199–204.
(25) *Rapid Overlay of Chemical Structures (ROCS), version 2.3.1*; OpenEye Scientific Software Inc.: Santa Fe, NM. Available at http://www.eyesopen.com (accessed Dec 1, 2008).
(26) Medina-Franco, J. L.; Maggiora, G. M.; Giulianotti, M. A.; Pinilla, C.; Houghten, R. A. A Similarity-Based Data-Fusion Approach to the Visual Characterization and Comparison of Compound Databases. *Chem. Biol. Drug Des.* **2007**, *70*, 393–412.
(27) Martínez-Mayorga, K.; Medina-Franco, J. L.; Giulianotti, M. A.; Pinilla, C.; Dooley, C. T.; Appel, J. R.; Houghten, R. A. Conformation-Opioid Activity Relationships of Bicyclic Guanidines from 3D Similarity Analysis. *Bioorg. Med. Chem.* **2008**, *16*, 5932–5938.
(28) Marín, R. M.; Aguirre, N. F.; Daza, E. E. Graph Theoretical Similarity Approach to Compare Molecular Electrostatic Potentials. *J. Chem. Inf. Model.* **2008**, *48*, 109–118.
(29) Frisch, M. J.; Trucks, G. W.; Schlegel, H. B.; Scuseria, G. E.; Robb, M. A.; Cheeseman, J. R.; Zakrzewski, V. G.; Montgomery, J. A., Jr.; Stratmann, R. E.; Burant, J. C.; Dapprich, S.; Millam, J. M.; Daniels, A. D.; Kudin, K. N.; Strain, M. C.; Farkas, O.; Tomasi, J.; Barone, V.; Cossi, M.; Cammi, R.; Mennucci, B.; Pomelli, C.; Adamo, C.; Clifford, S.; Ochterski, J.; Petersson, G. A.; Ayala, P. Y.; Cui, Q.; Morokuma, K.; Salvador, P.; Dannenberg, J. J.; Malick, D. K.; Rabuck, A. D.; Raghavachari, K.; Foresman, J. B.; Cioslowski, J.; Ortiz, J. V.; Baboul, A. G.; Stefanov, B. B.; Liu, G.; Liashenko, A.; Piskorz, P.; Komaromi, I.; Gomperts, R.; Martin, R. L.; Fox, D. J.; Keith, T.; Al-Laham, M. A.; Peng, C. Y.; Nanayakkara, A.; Challacombe, M.; Gill, P. M. W.; Johnson, B.; Chen, W.; Wong, M. W.; Andres, J. L.; Gonzalez, C.; Head-Gordon, M.; Replogle, E. S.; Pople, J. A. *Gaussian 98, Revision A.11*; Gaussian, Inc.: Pittsburgh, PA, 2001.
(30) Patterson, D. E.; Cramer, R. D.; Ferguson, A. M.; Clark, R. D.; Weinberger, L. E. Neighborhood Behavior: A Useful Concept for Validation Of "Molecular Diversity" Descriptors. *J. Med. Chem.* **1996**, *39*, 3049–3059.
(31) Peltason, L.; Bajorath, J. SAR Index: Quantifying the Nature of Structure-Activity Relationships. *J. Med. Chem.* **2007**, *50*, 5571–5578.
(32) Matter, H. Selecting Optimally Diverse Compounds from Structure Databases: A Validation Study of Two-Dimensional and Three-Dimensional Molecular Descriptors. *J. Med. Chem.* **1997**, *40*, 1219–1229.
(33) Muchmore, S. W.; Debe, D. A.; Metz, J. T.; Brown, S. P.; Martin, Y. C.; Hajduk, P. J. Application of Belief Theory to Similarity Data Fusion for Use in Analog Searching and Lead Hopping. *J. Chem. Inf. Model.* **2008**, *48*, 941–948.
(34) Shanmugasundaram, V.; Maggiora, G. M. *Characterizing Property and Activity Landscapes Using an Information-Theoretic Approach*. 222nd American Chemical Society National Meeting, Chicago, IL, United States, Cinf-032; American Chemical Society: Washington, DC, 2001.
(35) *Matlab, version 7.4.0.287*; The MathWorks, Inc.: Natick, MA. Available at http://www.mathworks.com (accessed Dec 1, 2008).
(36) *Spotfire, version 9.1.1*; TIBCO Software, Inc.: Somerville, MA. Available at http://spotfire.tibco.com (accessed Dec 1, 2008).
(37) Durant, J. L.; Leland, B. A.; Henry, D. R.; Nourse, J. G. Reoptimization of MDL Keys for Use in Drug Discovery. *J. Chem. Inf. Comput. Sci.* **2002**, *42*, 1273–1280.
(38) Peltason, L.; Bajorath, J. Molecular Similarity Analysis Uncovers Heterogeneous Structure-Activity Relationships and Variable Activity Landscapes. *Chem. Biol.* **2007**, *14*, 489–497.

CI800379Q
