# Supplementary material for: On the validity versus utility of activity landscapes: are all activity cliffs statistically significant?
Source: J Cheminform. 2014 Apr 2;6:11. doi: 10.1186/1758-2946-6-11 (PMC4021161; doi:10.1186/1758-2946-6-11)
Supplement: Additional file 1 — Code and data to reproduce figures. The ZIP file contains the R code and datasets required to perform the calculations and generate the figures for this article. It assumes you have R installed and are working on a Unix-like system. In addition you should have the ggplot2, grid, multicore and fingerprint R packages installed. To run the scripts execute run.sh from the command line. [file 1758-2946-6-11-S1.zip › si/bzd/2010 BMC-Antiprozoal Activity Landscape.pdf]

Bioorganic & Medicinal Chemistry 18 (2010) 7380–7391

Contents lists available at ScienceDirect

Bioorganic & Medicinal Chemistry

journal homepage: www.elsevier.com/locate/bmc

Towards a systematic characterization of the antiprotozoal activity landscape of benzimidazole derivatives

Jaime Pérez-Villanueva [a], Radleigh Santos [b], Alicia Hernández-Campos [a], Marc A. Giulianotti [b], Rafael Castillo [a], Jose L. Medina-Franco [b,*]

[a] Facultad de Química, Departamento de Farmacia, Universidad Nacional Autónoma de México, México DF 04510, Mexico
[b] Torrey Pines Institute for Molecular Studies, 11350 SW Village Parkway, Port St. Lucie, FL 34987, USA

ARTICLE INFO

Article history:
Received 9 August 2010
Revised 31 August 2010
Accepted 6 September 2010
Available online 15 September 2010

Keywords:
Activity cliffs
Giardia
Molecular similarity
SAS maps
Structure–activity relationships
Trichomonas

ABSTRACT

Parasitic infections caused by the protozoa *Trichomonas vaginalis* and *Giardia intestinalis* still represent a major problem in developing countries. Despite the fact that benzimidazoles are promising compounds with activity against both protozoa, systematic studies to characterize and compare their structure–activity relationships (SAR) are limited. Herein, we report a systematic characterization of the SAR of 32 benzimidazoles with activity against *T. vaginalis* and *G. intestinalis*. The analysis was based on pairwise comparisons of the activity similarity and molecular similarity using different molecular representations. Radial, MACCS keys, TGD and piDAPH3 fingerprints were used to develop consensus models of the landscape. The landscapes contained continuous regions and activity cliffs. Two 'deep consensus activity cliffs' and several pairs of compounds in smooth regions of the SAR were identified in the landscape of *T. vaginalis*. In contrast, a number of 'apparent and shallow cliffs' were found for *G. intestinalis*. Several compounds active for both parasites showed similar SAR suggesting a common mechanism of action. We also identified pairs of structurally similar molecules with dramatic changes in selectivity. Results suggested that while substitution at position 2 on the benzimidazole moiety plays an important role in increasing the potency against both parasites, substitutions at positions 4–7 could influence selectivity. This study represents a first step towards the systematic characterization of the antiprotozoal activity landscape of benzimidazoles, and has direct implications in the future development of other types of quantitative models. The landscape of larger data sets with other biological endpoints can be analyzed using the general approaches used in this work.

© 2010 Elsevier Ltd. All rights reserved.

1. Introduction

Parasitic diseases are still a major health problem in developing countries. Mucosal infections by protozoa infect more than a billon people every year. Among the most common protozoa infections are giardiosis, caused by *Giardia intestinalis*, and trichomonosis, a genitourinary infection caused by *Trichomonas vaginalis*.[1–3] As part of ongoing efforts to develop compounds as giardicidal and trichomonicidal agents, several benzimidazole derivatives have been synthesized and tested, leading to the identification of compounds in the low nanomolar range.[4–7] However, systematic and quantitative studies of the SAR of benzimidazoles as antiprotozoal agents are still limited.

Quantitative characterization of the SAR of small molecules plays a key role in lead optimization. To this end, a number of methods can be employed including quantitative structure–activity relationships (QSAR), rule-based methods, neural networks or pharmacophore modeling, to name a few examples.[8–11] However, several methodologies, like conventional QSAR, make assumptions that are not necessarily valid and, thus, may present misleading results including non-predictive models.[12] For example, one common assumption is that a lead series of compounds has a common binding mode or mechanism of action.[13,14] For this reason, understanding the activity landscape and early detection of activity cliffs[15] can be crucial to the success of computational models.[16]

The SAR of a data set can be conceptualized as an *activity landscape* where biological activity adds another dimension to the chemical space.[17] The activity landscape has been compared to rolling hills or continuous SAR where small changes in molecular structure are associated with small changes in activity.[15] A discontinuous SAR or rugged activity landscape, however, is populated with molecules with small changes in structure but large changes in activity (*activity cliffs*).[15] Such landscapes are common in lead optimization. It can also happen that structurally diverse compounds have similar activity, which is the basis of scaffold hopping.[18] Additionally, active regions with wide variations in chemical structure but small variations in biological activity may

* Corresponding author. Tel.: +1 772 345 4685; fax: +1 772 345 3649.
E-mail address: jmedina@tpims.org (J.L. Medina-Franco).

0968-0896/$ - see front matter © 2010 Elsevier Ltd. All rights reserved.
doi:10.1016/j.bmc.2010.09.019

suggest different binding modes or sites, or may reveal the effect of additional mechanisms such as the interaction with membranes that are not typically considered in several modeling approaches.[16] Understanding the activity landscape of a data set is, however, a difficult task because the landscape may be highly complex involving a combination of smooth and rugged regions.[14] Another major challenge is the dependence of chemical space on molecular representation.[19,20]

Different approaches are emerging to characterize systematically the activity landscape and are reviewed in Bajorath et al.[17] These include the structure–activity relationship index (SARI),[21] structure-landscape index (SALI),[16,22] structure–activity similarity (SAS) maps[23] and network-like similarity graphs (NSG).[24] SARI and NSG have been used to detect molecules with small changes in structure but large changes in selectivity (*selectivity cliffs*).[25] We proposed using multiple structural representations to derive a consensus model for the activity landscape and identify consensus activity cliffs.[26] Recently, 3D representations of the activity landscape were proposed, confirming the existence of consensus activity cliffs and representation-dependant cliffs.[27]

Herein we conducted a comprehensive analysis of the activity landscape of 32 benzimidazoles mostly synthesized and tested in our group against *T. vaginalis* and *G. intestinalis* (Table 1). Compounds are non 2-methylcarbamates and their mechanism of action remains unknown.[28,29] The analysis was based on pairwise comparisons of the activity similarity and molecular similarity. For each parasite, pairwise SAR was visually depicted using SAS maps. Quantitative analyses of the SAS maps were used to identify consensus activity cliffs and develop consensus models of the activity landscape. We also compared the SAR of the benzimidazoles between the two parasites.

2. Methods

2.1. Data set

The chemical structure of 32 previously reported benzimidazoles[4–7] is presented in Table 1 along with the biological activity against *T. vaginalis* and *G. intestinalis*. Table 1 lists the 50% inhibitory concentration (IC_{50}) in *in vitro* susceptibility assays for each parasite as pIC_{50} ($-\log IC_{50}$). For *T. vaginalis*, the activity ranges from 2 nM ($pIC_{50} = 8.7$) to 29,512 nM ($pIC_{50} = 4.53$); median $pIC_{50} = 6.7$. For *G. intestinalis*, the activity ranges from 22.9 nM ($pIC_{50} = 7.64$) to 10,471 nM ($pIC_{50} = 4.98$); median $pIC_{50} = 7.0$. The activity of the 32 compounds was obtained by the same group under similar conditions.

2.2. 2D and 3D structural similarity

For each pair of molecules m_i and m_j, pairwise *structural similarities* (SS_{ij}) were computed using the Tanimoto coefficient[30,31] with the following 2D molecular representations as implemented in molecular operating environment (MOE):[32] MACCS keys (166 bits), pharmacophore graph triangle (i.e., graph-based three point pharmacophores) (GpiDAPH3), typed graph distance (TGD), and typed graph triangle (TGT). We also used the following 2D (32-bit)

Table 1
Chemical structures of benzimidazoles and biological activity against *T. vaginalis* and *G. intestinalis*

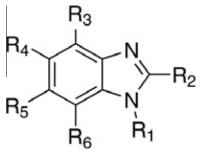

	R_1	R_2	R_3	R_4	R_5	R_6	pIC_{50} *T. vaginalis*	pIC_{50} *G. intestinalis*	ΔpIC_{50}	Ref.
**1**	H	CF_3	H	H	H	H	5.50	6.97	1.47	4
**2**	CH_3	CF_3	H	CF_3	H	H	5.39	5.94	0.55	4
**3**	CH_3	CF_3	H	H	CF_3	H	5.27	5.05	-0.22	4
**4**	CH_3	CF_3	H	Propylthio	H	H	6.70	4.98	-1.72	5
**5**	CH_3	CF_3	H	H	Propylthio	H	5.59	5.85	0.26	5
**6**	CH_3	CF_3	H	Benzoyl	H	H	4.53	5.96	1.43	5
**7**	CH_3	CF_3	H	H	Benzoyl	H	4.97	5.89	0.92	5
**8**	H	CF_3	H	Br	Br	H	6.66	6.92	0.26	6
**9**	H	CF_3	Br	Br	Br	Br	8.70	7.25	-1.45	6
**10**	H	C_2F_5	H	Cl	Cl	H	6.52	6.25	-0.27	6
**11**	H	CF_3	H	NO_2	NO_2	H	6.24	6.62	0.38	6
**12**	H	C_2F_5	Br	Br	Br	Br	5.00	7.64	2.64	6
**13**	CH_3	$CONH_2$	H	H	Cl	H	6.96	7.12	0.16	7
**14**	CH_3	$CONHCH_3$	H	H	Cl	H	6.98	7.15	0.17	7
**15**	CH_3	$CON(CH_3)_2$	H	H	Cl	H	6.63	7.40	0.77	7
**16**	CH_3	$COOCH_2CH_3$	H	H	Cl	H	7.72	7.32	-0.4	7
**17**	CH_3	$CONH_2$	H	Cl	H	H	6.73	6.63	-0.1	7
**18**	CH_3	$CONHCH_3$	H	Cl	H	H	6.45	6.45	0	7
**19**	CH_3	$CON(CH_3)_2$	H	Cl	H	H	6.68	6.61	-0.07	7
**20**	CH_3	$COOCH_2CH_3$	H	Cl	H	H	7.57	7.40	-0.17	7
**21**	CH_3	$CONH_2$	H	Cl	Cl	H	6.87	6.34	-0.53	7
**22**	CH_3	$CONHCH_3$	H	Cl	Cl	H	6.65	6.82	0.17	7
**23**	CH_3	$CON(CH_3)_2$	H	Cl	Cl	H	7.12	7.13	0.01	7
**24**	CH_3	$COOCH_2CH_3$	H	Cl	Cl	H	7.53	7.56	0.03	7
**25**	CH_3	$CONH_2$	H	H	H	H	6.78	7.03	0.25	7
**26**	CH_3	$CONHCH_3$	H	H	H	H	6.98	7.22	0.24	7
**27**	CH_3	$CON(CH_3)_2$	H	H	H	H	6.37	6.29	-0.08	7
**28**	CH_3	$COOCH_2CH_3$	H	H	H	H	7.07	7.16	0.09	7
**29**	CH_3	$COCH_3$	H	H	H	H	6.68	7.06	0.38	7
**30**	CH_3	$COCH_3$	H	Cl	H	H	6.88	7.30	0.42	7
**31**	CH_3	$COCH_3$	H	H	Cl	H	6.64	7.17	0.53	7
**32**	CH_3	$COCH_3$	H	Cl	Cl	H	7.20	7.46	0.26	7

fingerprints as implemented in Canvas:[33,34] radial (also known as extended connectivity fingerprints), dendritic, atom pairs and MOLPRINT 2D. To compute 3D similarities, a single low-energy conformation was considered for each molecule obtained with geometry optimization using the PM3 semiempirical method as implemented in Spartan'02.[35,36] 3D similarity values were calculated with the MOE pharmacophore atom triangle (piDAPH3) and pharmacophore atom quadruplet (piDAPH4) fingerprints, and Canvas 3- and 4-point pharmacophores. Despite the inherent conformational issues, the use of 3D structural representations were valuable to characterize the activity landscapes (see below).

2.3. Property similarity

The following properties were computed with Canvas: molecular weight (MW), number of rotatable bonds (RB), hydrogen bond acceptors (HBA), hydrogen bond donors (HBD), polar surface area (PSA), and the octanol/water partition coefficient ($A\log P$). Properties were first auto-scaled with mean centering using the equation:

$$p_{ki} = \frac{P_{ki} - \overline{P_k}}{\sigma_{P_k}} \quad (1)$$

where p_{ki} denotes the scaled version of the kth property for the ith molecule, P_{ki} denotes the unscaled value, and $\overline{P_k}$ and σ_{P_k} denote, respectively, the mean and standard deviation of the kth property over all molecules in the study.

The Euclidean distance between a pair of molecules was then computed with the expression:[31]

$$d_{ij} = \left[\sum_{k=1}^{K}(p_{ki} - p_{kj})^2\right]^{1/2} \quad (2)$$

where d_{ij} denotes the Euclidean distance between the ith and jth molecules, and p_{ki}, and p_{kj} denote the value of the scaled property k of the ith and jth molecules, respectively. In this study, $K = 6$.

Euclidean distances were scaled from 0 to 1 as follows:

$$sd_{ij} = \frac{d_{ij} - \min d_{ij}}{\max d_{ij} - \min d_{ij}} \quad (3)$$

where sd_{ij} is the scaled distance, and $\max d_{ij}$ and $\min d_{ij}$ indicate the range of distances in the data set. Pairwise property similarities were measured with the expression:

$$PS_{ij} = 1 - sd_{ij} \quad (4)$$

where PS_{ij} is the *property similarity* of the ith and jth molecules, and sd_{ij} is the scaled distance.

2.4. Activity similarity

For each pair of molecules the activity similarity for *T. vaginalis* and *G. intestinalis* was measured as follows:

$$AS_{i,j} = 1 - \frac{|A_i - A_j|}{\max - \min} \quad (5)$$

where A_i and A_j are the activities of the ith and jth molecules (pIC_{50} values) and max-min indicate the range of activities in the data set.

2.5. Activity landscape with SAS maps

For each target parasite, SAS maps[23] were generated by plotting the activity similarity against the structural similarity for each pair of compounds. A general form of the SAS map is presented in Figure 1. In this map the activity similarity is represented in the Y-axis and molecular similarity is plotted in the X-axis. A variant of the SAS maps represents potency difference in the Y-axis.[26,27]

SAS maps provide a visual and quantitative characterization of the activity landscape.[17] Four zones can be distinguished in Figure 1, labeled as regions I–IV. Data points that fall into region I correspond to pairs of molecules with high activity similarity and low molecular similarity and therefore are associated with regions of scaffold hopping. If the compounds in the data set share the same core scaffold and the differences are only in the attachment points, then region I is associated with *side chain hopping*.[37] Points plotted in region II denote pairs of molecules with high molecular similarity and high activity similarity. Thus, compounds in this region are in a smooth or continuous SAR landscape. Data points in region III denote pairs of molecules with low molecular similarity and low activity similarity. Region IV identifies pairs of molecules that have high molecular similarity and low activity similarity and therefore correspond to activity cliffs or discontinuous SAR. Data points that are consistently put in the same region by a number of molecular representations contribute to defining a *consensus model* of the activity landscape.

In order to characterize the SAS maps obtained with different similarity measures, each map was partitioned by imposing activity and molecular similarity thresholds along the Y- and X-axis, respectively, and then counting the number of data pairs in the resultant regions I–IV. A similar strategy was recently employed to successfully characterize potency difference vs. structure similarity plots.[26] In this study, two activity similarity thresholds were investigated, namely, 0.5 and 0.75, corresponding approximately to 1 and 2 log units in potency difference for *T. vaginalis,* and 0.7 and 1.4 log units for *G. intestinalis*. For similarity, the median similarity of the most active compounds in the data set was considered. For *T. vaginalis*, seven compounds with $pIC_{50} \geqslant 7.00$ ($IC_{50} \leqslant 100$ nM) were regarded as active (**9**, **16**, **20**, **23**, **24**, **28** and **32**). For *G. intestinalis*, also seven compounds with $pIC_{50} \geqslant 7.30$ ($IC_{50} \leqslant 50$ nM) were regarded as active (**12**, **15**, **16**, **20**, **24**, **30** and **32**). The reason to use slightly different thresholds of pIC_{50} was twofold; in order to select the same number of actives for each parasite and because the median of the pIC_{50} values for *G. intestinalis* is greater than the median for *T. vaginalis* (see above). Note, however, that other thresholds for activity could be applied. Since different molecular representations lead to different ranges of similarity values for the same set of compounds, the threshold for the structural similarity depends on the representation used. Therefore, normalizing to the median similarity of a descriptor allowed us the ability to compare between different descriptor sets.[26]

To further compare the SAS maps obtained from different structural similarity methods, we used the *Degree of Consensus* (DoC) introduced in an earlier study.[26] DoC measures the number of data

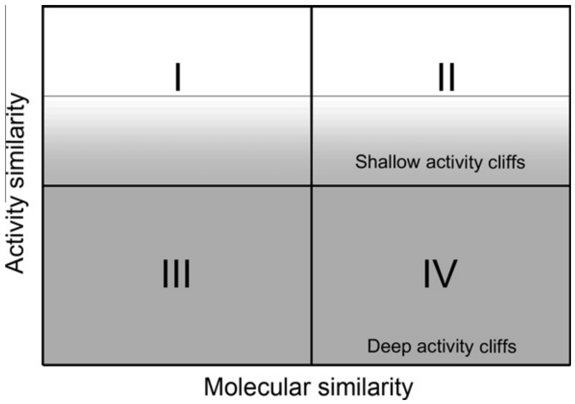

Figure 1. General form of the structure–activity similarity (SAS) map showing four major regions. Regions I and II contain data pairs for scaffold hopping and smooth SAR, respectively. Region IV indicates discontinuous SAR and activity cliffs. Regions of deep and shallow activity cliffs are also shown. See text for details.

points consistently put into the same region and was computed with the following expression:

$$\mathrm{DoC}^{R}_{m,n} = \frac{Cp_{m,n}}{p_m + p_n - Cp_{m,n}} \quad (6)$$

where $Cp_{m,n}$ is the number of *Consensus Pairs* in region R (R = I–IV) between methods m and n; p_m is the number of pairs of molecules assigned by method m in region R, and p_n is the number of pairs of molecules assigned by method n in the same region. To note, DoC depends on the thresholds used to define each region. Results were summarized as DoC matrices.

2.6. Consensus SAS maps

To develop consensus models of the activity landscapes, we combined structural similarities obtained with uncorrelated representations into a single similarity measure. There are a number of ways to combine similarity values.[38] In this work we computed the mean similarity of four orthogonal fingerprints for this data set (radial, MACCS keys, TGD and piDAPH3), but other measures can be explored. Property similarity was not considered in order to obtain the mean. Consensus SAS maps for each parasite were generated by plotting the activity similarity against the mean fingerprint similarity.

3. Results and discussion

3.1. Distribution of fingerprint similarity measures

The 496 pairwise similarities of the 32 benzimidazoles calculated with the 12 fingerprint-based structural representations are summarized in Figure 2 as cumulative distribution functions (CDF). The table at the bottom of the figure summarizes the statistics of each curve indicating the maximum, third and first quartile, median, mean, and standard deviation.

2D and 3D fingerprints showed a wide variation of distributions. 2D fingerprints with the highest similarity values were TGD, MACCS keys and GpiDAPH3 which had median values of 0.80, 0.68 and 0.52, respectively, and comparable standard deviation (0.16–0.18). 2D fingerprints with the lowest similarity values were dendritic, atom pairs, MOLPRINT 2D and radial. Concerning the 3D fingerprints, the spatial three-point pharmacophore piDAPH3 had a nearly normal distribution. In contrast, similarity values obtained with piDAPH4, 3- and 4-point pharmacophores showed non-normal distributions (as can be deduced from the non-sigmoidal shape of the corresponding CDF). Despite the fact that the 32 molecules share the benzimidazole scaffold, it was noteworthy that several fingerprints were able to differentiate the compounds, thus identifying activity cliffs (see below).

3.2. Correlation between molecular similarities

The correlation between 2D and 3D fingerprint representations for the 496 pairwise similarities is shown in Table 2. The correlation matrix shows the relationships between the 12 fingerprints. Additionally, the matrix shows the relationship between the fingerprint, property similarity, and activity similarity. Very high correlations between 2D methods occur for radial and dendritic; MOLPRINT 2D and atom pairs; radial and atom pairs; dendritic and atom pairs; TGD and TGT (correlation $\geqslant$ 0.92). Other high correlations between 2D methods are atom pairs and MACCS (0.89); MOLPRINT 2D and MACCS (0.84); atom pairs and GpiDAPH3 (0.83). High correlations between 3D fingerprints occur for Canvas 3- and 4-point pharmacophores (0.97), and between piDAPH3 and 3-point pharmacophore (0.82). Comparing the correlation between 2D and 3D fingerprints,

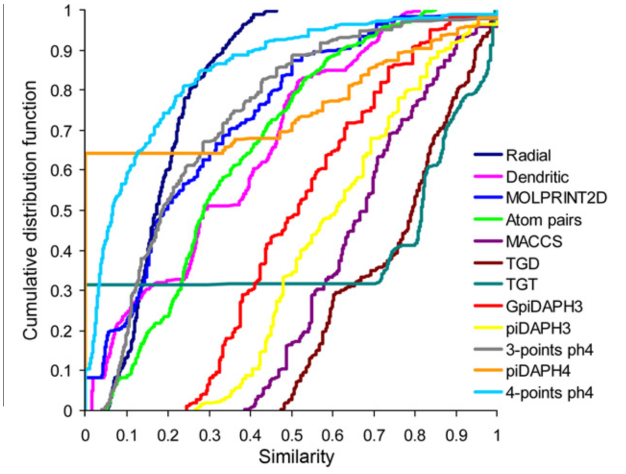

	Max	Q3	Median	Q1	Min	Mean	STD
Radial	0.46	0.24	0.17	0.13	0.05	0.19	0.09
Dendritic	0.81	0.48	0.29	0.11	0.02	0.33	0.23
MOLPRINT 2D	1.00	0.42	0.20	0.12	0.00	0.27	0.22
Atom pairs	0.85	0.49	0.29	0.21	0.05	0.34	0.20
MACCS	1.00	0.80	0.68	0.55	0.39	0.68	0.16
TGD	1.00	0.88	0.80	0.59	0.47	0.76	0.16
TGT	1.00	0.90	0.82	0.00	0.00	0.60	0.41
GpiDAPH3	1.00	0.69	0.52	0.38	0.25	0.54	0.18
piDAPH3	1.00	0.76	0.61	0.46	0.27	0.62	0.18
3-points ph4	1.00	0.37	0.19	0.11	0.04	0.27	0.21
piDAPH4	1.00	0.58	0.00	0.00	0.00	0.24	0.34
4-points ph4	1.00	0.20	0.07	0.03	0.00	0.15	0.20

Figure 2. Cumulative distribution functions of 496 pairwise structural similarities using different 2D and 3D fingerprint representations. The table summarizes the information of the distributions. Q3 and Q1 indicate the third and first quartile, respectively.

the highest correlation was for GpiDAPH3 and piDAPH3 (0.94). The correlation between property similarity and fingerprint similarity ranges between 0.44 (piDAPH4) and 0.78 (atom pairs and MACCS). The matrix also shows a low correlation between any of the molecular representations with activity similarity for *T. vaginalis* (correlation $\leqslant$ 0.48) or *G. intestinalis* ($\leqslant$ 0.30). The correlation between activity similarities for *T. vaginalis* and *G. intestinalis* was low (0.31) indicating the presence of pairs of compounds with different effects against the two parasites (see below).

Despite the high correlations between several 2D and 3D fingerprints, we selected as many orthogonal fingerprint representations as possible to characterize the landscapes. Thus, we selected radial, MACCS, TGD (2D), and piDAPH3 (3D). The maximum correlation between any of these five selected fingerprints was 0.78 (radial and MACCS), and the minimum correlation was 0.69 (TGD and piDAPH3). In addition, we employed property similarities as described below.

3.3. Activity landscape

3.3.1. SAS maps

Figures 3 and 4 depict the SAS maps for *T. vaginalis* and *G. intestinalis*, respectively. The maps show the relationship between activity similarity and molecular similarity obtained with four selected fingerprints and property similarity. Each plot contains 496 data points that represent a pairwise comparison. Data points were further distinguished by the activity of the most active compound in the pair in a continuous scale from green (least active) to red (most active). It is also possible to generate a visual representation of the plots coloring only data points where at least one compound in the pair is active (Figs. S1 and S2 in Supplementary data).[26]

Table 2
Correlation matrix for the pairwise activity, property and structure similarities

	Radial	Dendritic	MOLPRINT 2D	Atom pairs	MACCS	TGD	TGT	GpiDAPH3	piDAPH3	3-point ph4	piDAPH4	4-point ph4	Properties	AS *T. vaginalis*	AS *G. intestinalis*
Radial	1.00														
Dendritic	0.94	1.00													
MOLPRINT 2D	0.88	0.86	1.00												
Atom pairs	0.92	0.92	0.93	1.00											
MACCS	0.78	0.81	0.84	0.89	1.00										
TGD	0.71	0.82	0.69	0.76	0.77	1.00									
TGT	0.69	0.82	0.63	0.71	0.73	0.93	1.00								
GpiDAPH3	0.79	0.76	0.77	0.83	0.76	0.69	0.56	1.00							
piDAPH3	0.76	0.73	0.74	0.79	0.73	0.69	0.57	0.94	1.00						
3-point ph4[a]	0.78	0.75	0.81	0.83	0.77	0.68	0.55	0.82	0.81	1.00					
piDAPH4	0.40	0.36	0.43	0.48	0.53	0.32	0.19	0.61	0.62	0.49	1.00				
4-point ph4[a]	0.73	0.69	0.78	0.79	0.72	0.63	0.49	0.78	0.77	0.97	0.50	1.00			
Properties	0.69	0.69	0.74	0.78	0.78	0.65	0.59	0.73	0.74	0.75	0.44	0.69	1.00		
AS[b] *T. vaginalis*	0.43	0.40	0.44	0.45	0.28	0.26	0.19	0.48	0.47	0.42	0.25	0.39	0.37	1.00	
AS[b] G. *intestinalis*	0.28	0.20	0.28	0.25	0.15	-0.01	-0.05	0.29	0.30	0.27	0.17	0.26	0.20	0.31	1.00

[a] ph4: pharmacophore
[b] AS: activity similarity

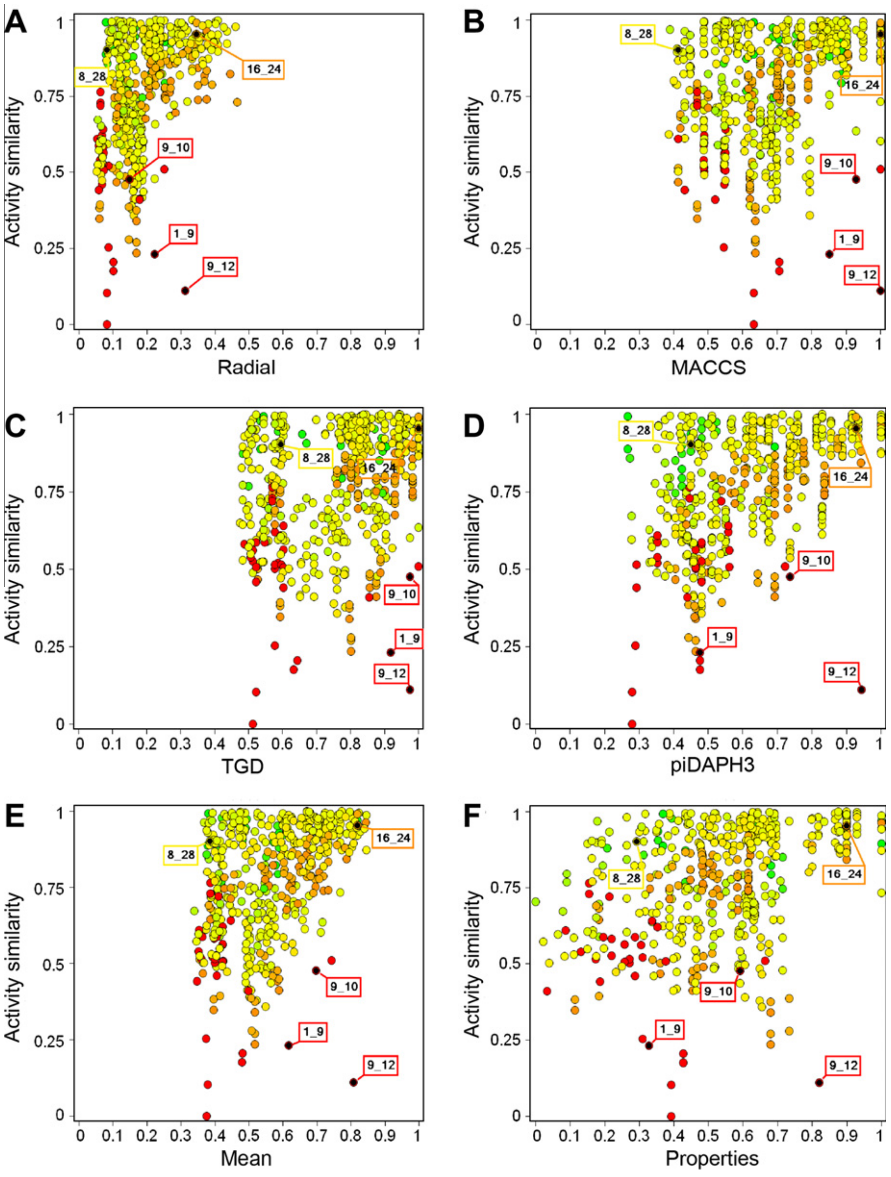

Figure 3. SAS maps for *T. vaginalis* with different structural representations. Each data point indicates a pairwise comparison of 32 benzimidazoles (496 data points). Data points are color-coded by the activity of the most active compound in the pair using a continuous scale from green (less active) to red (more active). Each panel corresponds to a different structural representation: (A) Radial; (B) MACCS keys; (C) TGD; (D) piDAPH3; (E) mean fingerprint similarity, and (F) properties. Selected pairs are marked in black and labeled with the compound numbers.

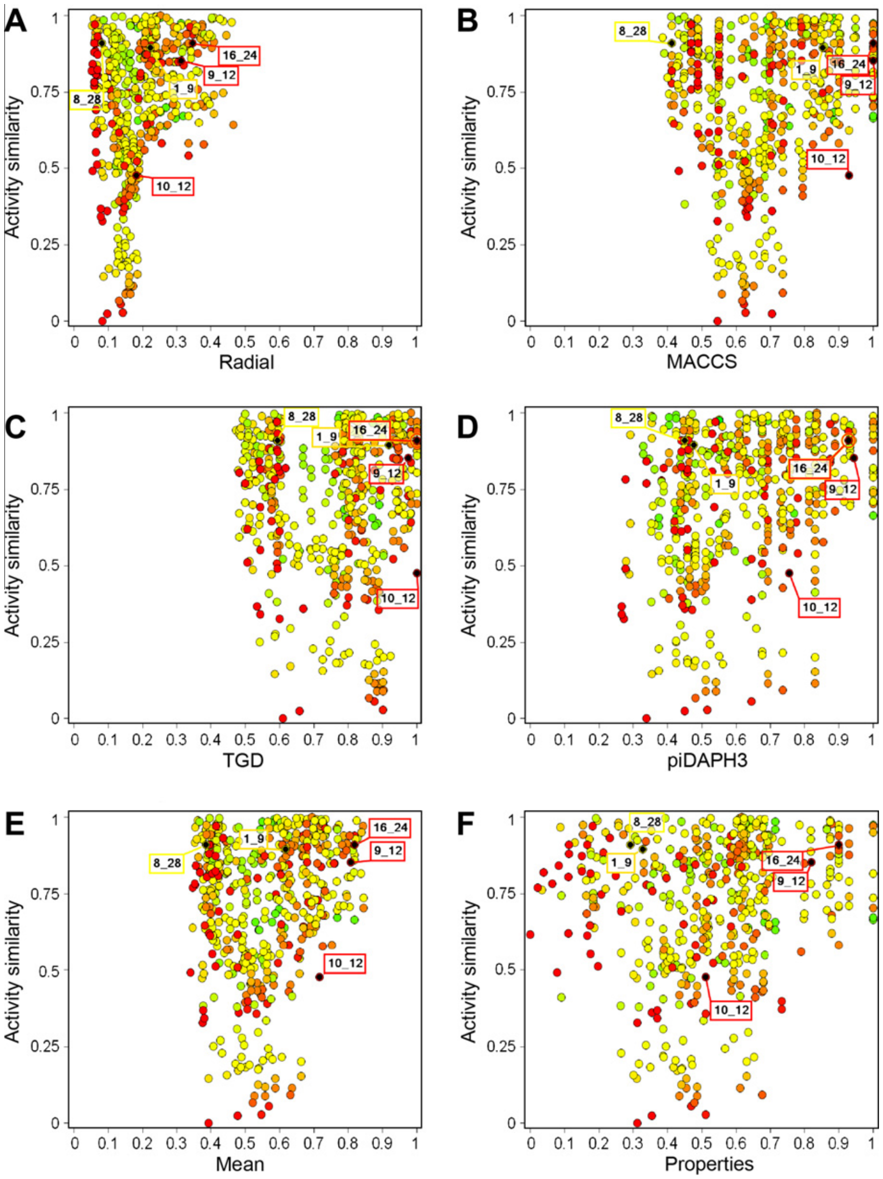

Figure 4. SAS maps for *G. intestinalis* with different structural representations. Each data point indicates a pairwise comparison of 32 benzimidazoles (496 data points). Data points are color-coded by the activity of the most active compound in the pair using a continuous scale from green (less active) to red (more active). Each panel corresponds to a different structural representation: (A) Radial; (B) MACCS keys; (C) TGD; (D) piDAPH3; (E) mean fingerprint similarity, and (F) properties. Selected pairs are marked in black and labeled with the compound numbers.

As expected from the CDF in Figure 2, similarity values obtained with radial fingerprints are shifted to the low similarity value range along the *X*-axis (<0.46) while the similarity values for MACCS, TGD and piDAPH3 are more spread out. Interestingly, there are several pairs in Figures 3 and 4 with similarities of 1.0 (21 pairs for MACCS keys, 15 for TGD, 18 for piDAPH3 and 8 for property similarity). A number of these points correspond to positional isomers or compounds with different halogen substitution pattern (discussed below). Notably, radial fingerprints can distinguish all 496 pairs due to its high resolution. This is an expected behavior since radial fingerprints were designed for structure activity studies in contrast with topological fingerprints that were developed for substructure and similarity searching.[34,39]

As described in the Section 2, the four regions of the SAS maps (I–IV in Fig. 1) can also be identified in Figures 3 and 4. Data pairs in regions I and II are located in a continuous SAR while pairs of molecules in region IV represent activity cliffs. As discussed above, the boundary between regions I/II and III/IV depends on the molecular representation used. However, it is possible to detect pairs of compounds that are located in the same *relative* region of each map, that is, *consensus pairs*. Figures 3 and 4 show examples of consensus pairs in regions I (compounds **8** and **28**) and II (**16** and **24**) for *T. vaginalis* and *G. intestinalis*. Figure 3 also shows a consensus pair in region IV (**9** and **12**) for *T. vaginalis*.

3.3.2. Quantitative characterization of the SAS maps

In order to conduct a systematic and quantitative analysis of the data obtained in this study, the SAS maps were divided into four quadrants (regions I–IV in Fig. 1) by defining thresholds for activity similarity and molecular similarity (see Section 2). Table 3 indicates the median similarity of the active compounds and the number of data pairs that can be found in regions I–IV for different molecular representations. Table 3 also summarizes the number of pairs with at least one active molecule or *active pair*. Interestingly, the median fingerprint similarity of the actives for *T. vaginalis* and *G. intestinalis* (Table 3) is slightly higher than the median similarity of the 32 compounds (Fig. 2). Data in Table 3 can be visualized

Table 3
Distribution of data points in different regions of the SAS maps

Representation (*AS* threshold) [a]	Median similarity of actives[b]	I		II		III		IV	
		Total[c]	Active pairs[d]	Total	Active pairs	Total	Active pairs	Total	Active pairs
T. vaginalis (0.5)									
Radial	0.22	286	94	160	66	47	34	3	2
MACCS	0.71	271	102	175	58	42	31	8	5
TGD	0.95	401	138	45	22	48	34	2	2
piDAPH3	0.78	346	129	100	31	49	35	1	1
Properties	0.62	285	120	161	40	38	25	12	11
T. vaginalis (0.75)									
Radial	0.22	145	50	149	59	188	78	14	9
MACCS	0.71	142	59	152	50	171	74	31	13
TGD	0.95	254	89	40	20	195	83	7	4
piDAPH3	0.78	204	80	90	29	191	84	11	3
Properties	0.62	160	74	134	35	163	71	39	16
G. intestinalis (0.5)									
Radial	0.24	301	83	114	50	81	38	0	0
MACCS	0.79	286	91	129	42	76	35	5	3
TGD	0.93	327	90	88	43	80	37	1	1
piDAPH3	0.84	356	113	59	20	81	38	0	0
Properties	0.61	241	87	174	46	63	26	18	12
G. intestinalis (0.75)									
Radial	0.24	184	54	76	35	198	67	38	15
MACCS	0.79	171	58	89	31	191	68	45	14
TGD	0.93	199	57	61	32	208	70	28	12
piDAPH3	0.84	216	72	44	17	221	79	15	3
Properties	0.61	138	54	122	35	166	59	70	23

[a] Regions I–IV are defined by the median similarity of active compounds and a threshold of activity similarity. Two thresholds of activity similarity (AS) were investigated 0.5 and 0.75 (see also Fig. 1 and text for details).
[b] Median similarity of compounds **9**, **16**, **20**, **23**, **24**, **28**, **32** for *T. vaginalis*, and **12**, **15**, **16**, **20**, **24**, **30**, **32** for *G. intestinalis*.
[c] Total number of data points (pair of compounds) in the region.
[d] Number of data points with at least one active compound in the pair.

using pie charts (Fig. S3 in Supplementary data illustrates examples of binned SAS maps[26]).

Considering an activity similarity threshold of 0.5 for *T. vaginalis* and *G. intestinalis*, most pairs of compounds were in region I, and the frequency decreased in the order I > II > III > IV (Table 3). This occurred for most fingerprint representations and property similarity.[40] A similar result was obtained for the number of active pairs.

Not surprisingly, considering an activity similarity threshold of 0.75, the total number of pairs and active pairs in regions I and II decreased, and the number of pairs in regions III and IV increased (as compared to the populations at a threshold of 0.5). This was observed for all representations and for both parasites (Table 3). However, for *G. intestinalis*, most of the pairs were in region III for all molecular representations (and most representations for *T. vaginalis*). Similar results were obtained for the number of active pairs. In general, at the activity similarity threshold of 0.75, the frequency of total pairs and active pairs decreased in the order III > I > II > IV (Table 3).

3.3.3. Deep and shallow activity cliffs

The number of activity cliffs in the data set depends on the criteria used to define a pair of compounds as similar. It follows that activity cliffs can be classified further using, for example, different thresholds of activity similarity. In this work we define a *deep activity cliff* if the pair of similar compounds have 'a large' difference in activity (activity similarity $\leqslant 0.5$), and define a *shallow activity cliff* if the difference in activity is smaller ($0.5 <$ activity similarity $\leqslant 0.75$).[27] This is schematically illustrated in Figure 1. Different activity similarity thresholds can be used to define deep and shallow cliffs. Moreover, the degree of molecular similarity can be used also to define deep or shallow activity cliffs. According to these definitions, there were between one and eight deep activity cliffs in the landscape of *T. vaginalis*, depending on the fingerprint representation. The number of deep activity cliffs for *G. intestinalis* was lower, between zero and five (i.e., the number of deep activity cliffs equals the number of pairs in region IV at activity threshold of 0.5, Table 3). Considering molecular properties as molecular representation, the number of deep cliffs for *T. vaginalis* and *G. intestinalis* was 12 and 18, respectively. Noteworthy, the numbers of deep and shallow cliffs, as defined in this work, are relative to the data set. This is because deep and shallow cliffs are defined based on activity similarity that depends on the activity range of the data set (Eq. 5).

The number of shallow activity cliffs can be calculated from Table 3 taking the difference between the number of total pairs in region IV at activity similarity thresholds of 0.75 and 0.5. For example, for *T. vaginalis*, there are $14 - 3 = 11$ shallow cliffs considering radial fingerprints and $31 - 8 = 23$ shallow cliffs considering MACCS keys. Table S1 in the Supplementary data summarizes the number of shallow activity cliffs for each parasite and each molecular representation. Noteworthy, for all molecular representations, the number of shallow cliffs for *G. intestinalis* was higher than the number of shallow cliffs for *T. vaginalis*. Examples of deep and shallow activity cliffs are discussed below.

Results above, and in previous studies, support the importance of considering several representations[26,27] and lead to the following questions: are there *consensus pairs*?[26] Is it possible to derive a consensus model of the activity landscape for a given data set?

3.3.4. Degree of consensus

Despite the mid-to-low correlations between the molecular representations (Table 2) and the different distributions of pairwise similarity values (Fig. 2), it was possible to find a number of consensus pairs in different regions of the landscape. The number of pairs of compounds that two methods put into the same region,

T. vaginalis (Activity similarity threshold 0.5)

Region I

	Radial	MACCS	TGD	piDAPH3	Properties
Radial	1.00				
MACCS	0.81	1.00			
TGD	0.67	0.62	1.00		
piDAPH3	0.77	0.73	0.80	1.00	
Properties	0.72	0.79	0.67	0.74	1.00

Region II

	Radial	MACCS	TGD	piDAPH3	Properties
Radial	1.00				
MACCS	0.71	1.00			
TGD	0.20	0.17	1.00		
piDAPH3	0.52	0.49	0.28	1.00	
Properties	0.55	0.68	0.20	0.47	1.00

Region III

	Radial	MACCS	TGD	piDAPH3	Properties
Radial	1.00				
MACCS	0.85	1.00			
TGD	0.94	0.88	1.00		
piDAPH3	0.96	0.86	0.98	1.00	
Properties	0.77	0.70	0.76	0.78	1.00

Region IV

	Radial	MACCS	TGD	piDAPH3	Properties
Radial	1.00				
MACCS	0.22	1.00			
TGD	0.25	0.25	1.00		
piDAPH3	0.33	0.13	0.50	1.00	
Properties	0.15	0.18	0.08	0.08	1.00

G. intestinalis (Activity similarity threshold 0.5)

Region I

	Radial	MACCS	TGD	piDAPH3	Properties
Radial	1.00				
MACCS	0.77	1.00			
TGD	0.75	0.76	1.00		
piDAPH3	0.77	0.80	0.87	1.00	
Properties	0.67	0.77	0.68	0.68	1.00

Region II

	Radial	MACCS	TGD	piDAPH3	Properties
Radial	1.00				
MACCS	0.52	1.00			
TGD	0.39	0.44	1.00		
piDAPH3	0.34	0.45	0.50	1.00	
Properties	0.45	0.63	0.42	0.34	1.00

Region III

	Radial	MACCS	TGD	piDAPH3	Properties
Radial	1.00				
MACCS	0.94	1.00			
TGD	0.99	0.95	1.00		
piDAPH3	1.00	0.94	0.99	1.00	
Properties	0.78	0.76	0.77	0.78	1.00

Region IV

	Radial	MACCS	TGD	piDAPH3	Properties
Radial	NA				
MACCS	0.00	1.00			
TGD	0.00	0.20	1.00		
piDAPH3	NA	0.00	0.00	NA	
Properties	0.00	0.10	0.00	0.00	1.00

T. vaginalis (Activity similarity threshold 0.75)

Region I

	Radial	MACCS	TGD	piDAPH3	Properties
Radial	1.00				
MACCS	0.74	1.00			
TGD	0.52	0.49	1.00		
piDAPH3	0.69	0.66	0.74	1.00	
Properties	0.65	0.79	0.58	0.70	1.00

Region II

	Radial	MACCS	TGD	piDAPH3	Properties
Radial	1.00				
MACCS	0.75	1.00			
TGD	0.20	0.17	1.00		
piDAPH3	0.57	0.54	0.31	1.00	
Properties	0.63	0.78	0.23	0.56	1.00

Region III

	Radial	MACCS	TGD	piDAPH3	Properties
Radial	1.00				
MACCS	0.89	1.00			
TGD	0.93	0.87	1.00		
piDAPH3	0.90	0.85	0.93	1.00	
Properties	0.80	0.78	0.80	0.80	1.00

Region IV

	Radial	MACCS	TGD	piDAPH3	Properties
Radial	1.00				
MACCS	0.36	1.00			
TGD	0.24	0.19	1.00		
piDAPH3	0.14	0.17	0.13	1.00	
Properties	0.15	0.25	0.07	0.11	1.00

G. intestinalis (Activity similarity threshold 0.75)

Region I

	Radial	MACCS	TGD	piDAPH3	Properties
Radial	1.00				
MACCS	0.76	1.00			
TGD	0.76	0.75	1.00		
piDAPH3	0.76	0.78	0.86	1.00	
Properties	0.62	0.75	0.65	0.64	1.00

Region II

	Radial	MACCS	TGD	piDAPH3	Properties
Radial	1.00				
MACCS	0.54	1.00			
TGD	0.44	0.49	1.00		
piDAPH3	0.38	0.48	0.54	1.00	
Properties	0.45	0.65	0.44	0.36	1.00

Region III

	Radial	MACCS	TGD	piDAPH3	Properties
Radial	1.00				
MACCS	0.84	1.00			
TGD	0.84	0.83	1.00		
piDAPH3	0.86	0.86	0.92	1.00	
Properties	0.76	0.79	0.74	0.75	1.00

Region IV

	Radial	MACCS	TGD	piDAPH3	Properties
Radial	1.00				
MACCS	0.43	1.00			
TGD	0.29	0.33	1.00		
piDAPH3	0.26	0.33	0.39	1.00	
Properties	0.37	0.46	0.27	0.21	1.00

Figure 5. Degree of consensus (DoC) matrices for each region. Each entry in the corresponding matrix represents the agreement between two methods to place a pair of compounds into the same region. DoC is computed with Eq. 6 using data in Figure S4.

that is, number of consensus pairs, were recorded for *T. vaginalis* and *G. intestinalis* at the two activity similarity thresholds. Results are summarized in Figure S4 in Supplementary data. For both parasites, we identified several consensus pairs in all regions of the landscape at the two thresholds. The only exception was region IV for *G. intestinalis* at a threshold of 0.5; we found only one consensus pair between MACCS and TGD and two consensus pairs between MACCS and property similarity (Fig. S4). Examples of consensus pairs are discussed later in this section. The DoC between two methods is presented in Figure 5. DoC measures the number of consensus pairs between two methods scaled by the total number of pairs that the two methods put into the same region (see Section 2). For both parasites, at the two activity similarity thresholds, DoC has high values in region III (0.70–1.00) followed by region I (0.49–0.98). In contrast, DoC has low values in region IV, in particular for *G. intestinalis* at activity similarity threshold of 0.5 (0–0.2). This means that there was better agreement between the methods used to assign molecules to region III than in any other

Table 4
Examples of consensus pairs of compounds in the SAS maps

Pair	Activity similarity		Fingerprint similarity					Property similarity
	T. vaginalis	*G. intestinalis*	Radial	MACCS	TGD	piDAPH3	Mean (STD)	
**4_28**[a]	0.911	0.180	0.138	0.612	0.848	0.561	0.540 (0.295)	0.443
**8_28**[a]	0.902	0.910	0.082	0.413	0.594	0.450	0.385 (0.216)	0.291
**10_28**[a]	0.868	0.658	0.080	0.413	0.618	0.487	0.400 (0.229)	0.348
**21_28**[a]	0.952	0.692	0.188	0.617	0.805	0.620	0.557 (0.262)	0.503
**28_32**[a,b]	0.969	0.887	0.190	0.698	0.945	0.693	0.631 (0.317)	0.603
**7_12**[b]	0.993	0.342	0.079	0.632	0.544	0.266	0.380 (0.254)	0.369
**16_20**[a,b]	0.964	0.970	0.375	1.000	1.000	1.000	0.844 (0.313)	1.000
**16_24**[a,b]	0.954	0.910	0.346	1.000	1.000	0.928	0.818 (0.317)	0.900
**16_28**[a,b]	0.844	0.940	0.351	0.905	0.949	0.941	0.786 (0.291)	0.900
**15_16**[a,b]	0.739	0.970	0.351	0.708	0.983	0.836	0.719 (0.270)	0.616
**16_19**[a,b]	0.751	0.733	0.253	0.708	0.983	0.836	0.695 (0.315)	0.616
**16_30**[a,b]	0.799	0.992	0.269	0.791	0.931	0.836	0.707 (0.297)	0.615
**16_31**[a,b]	0.741	0.944	0.375	0.791	0.931	0.836	0.733 (0.246)	0.615
**8_9**[a]	0.511	0.876	0.250	1.000	1.000	0.723	0.743 (0.354)	0.664
**1_9**[a]	0.233	0.895	0.222	0.852	0.918	0.476	0.617 (0.327)	0.327
**9_10**[a]	0.477	0.624	0.148	0.929	0.975	0.737	0.697 (0.380)	0.591
**9_12**[a]	0.113	0.853	0.313	1.000	0.975	0.944	0.808 (0.331)	0.819
**10_12**[b]	0.635	0.477	0.182	0.929	1.000	0.756	0.717 (0.371)	0.511

[a] Active pair for *T. vaginalis*.
[b] Active pair for *G. intestinalis*.

region. In contrast, it was more difficult to identify consensus activity cliffs than to identify consensus pairs in continuous regions of the SAR. Note that DoC is dependent on the criteria used to define the four regions.

Table 4 lists several examples of consensus pairs in the three most informative regions (I–II and IV) of the SAS map of *T. vaginalis* and *G. intestinalis*. Table 4 also lists the molecular similarity for selected fingerprint representations, property similarity and activity similarity.

For *T. vaginalis*, several examples of consensus pairs with low structural similarity and high activity similarity (region I) involve the active compound **28** (IC_{50} = 86 nM) such as **4_28**, **8_28**, **10_28**, **21_28** and **32_28** (Table 4). Figure 6A shows a comparison of the chemical structures for selected pairs in region I along with the activity and molecular similarity measures. In this figure, concentric ovals indicate different degrees of structural similarity to **28**. For example, compounds **8** and **10** are less similar to **28** as compared to **4**, **21** and **32**. Interestingly, **8** and **10** are also less active than **4**, **21** and **32**. Since all compounds in the set have the same scaffold, pairs in region I can be considered examples of side chain hopping (see above). Noteworthy, several whole-molecule fingerprints used in this study were able to detect low similarity due to the side chain substitutions. For example, the similarity for the above mentioned pairs with the known 'low resolution' 166-bit MACCS keys[39] ranges between 0.698 (**32_28**) and 0.413 (**8_28** and **10_28**). In contrast, for the same pairs, the radial similarity ranges between 0.190 (**32_28**) and 0.080 (**10_28**).

Some of the pairs in region I of the landscape of *T. vaginalis* were also in region I of the landscape of *G. intestinalis*. Examples were **8_28** and **28_32** with activity similarly values of 0.910 and 0.887, respectively (Table 4 and Fig. 6A). These results suggest a similar SAR for both parasites. Notable exceptions were **4_28** and **7_12** which have low activity similarity for *G. intestinalis* (0.18 and 0.342, respectively) indicating that in some instances the same change in the structure of the benzimidazoles produces different effects in the activity of *T. vaginalis* and *G. intestinalis*.

We also identified several consensus pairs in region II of the landscape of *T. vaginalis*. To note, a number of these pairs, with high structure similarity and high activity similarity (>0.84), included the active compound **16** (IC_{50} = 19 nM). Examples are pairs **16_20**, **16_24** and **16_28** (Fig. 6B). Interestingly, several fingerprint representations including MACCS keys, TGD and piDAPH3 were unable to distinguish the positional isomer **16_20** (similarity of 1.0). However, the radial fingerprint did differentiate this pair demonstrating the high resolution of this type of fingerprint.[39] All compounds in these pairs have an ethyl ester at R_2 and are in a smooth region of landscape; changes in the substitution pattern with chlorine at positions 5 and 6 of the benzimidazole scaffold produce small changes in the activity (activity similarity between 0.844 and 0.964).

The pairs **16_15**, **16_19**, **16_30** and **16_31** are also located in region II of the landscape of *T. vaginalis* (Fig. 6B). The structural similarity of **15**, **19**, **30** and **31** with respect to **16** decreases (as captured by several molecular representations), and the activity similarity also decreases (down to 0.739–0.799). These results, schematically illustrated in Figure 6B, are in agreement with the 'similarity principle'[41] and further illustrate the smooth SAR associated with region II. To note, none of these compounds have an ethyl ester at R_2 emphasizing the importance of this substituent in the activity against *T. vaginalis*.

Several pairs with high structural similarity and high activity similarity for *T. vaginalis* were also located in region II of the landscape of *G. intestinalis* as illustrated by the pairs containing compound **16** (IC_{50} = 48 nM, Table 4). These results indicate that, in general, substitution with ethyl ester at R_2 increases the activity against both parasites. Similarly, substitution with one or two chlorine atoms at positions 5 and 6 of the benzimidazole scaffold does not affect significantly the activity against either parasite.

3.3.5. Consensus deep and shallow activity cliffs, and apparent cliffs

The importance of activity cliffs has been discussed in the literature.[15,17,19] Activity cliffs are valuable for detecting specific structural changes important for activity. Furthermore, consensus activity cliffs have been conceptualized as those cliffs that occur across different molecular representations.[26] Figure 6C depicts examples of consensus cliffs (region IV) for different molecular representations.

For *T. vaginalis*, we found only one *consensus deep cliff*, pair **9_12** (cliff for all molecular representations with at least two log units in potency difference, Table 4). The structural difference between **9** and **12** is a CF_2 group at R_2 (CF_3 vs CF_2–CF_3). This change in structure produces a dramatic decrease in activity against *T. vaginalis* from IC_{50} = 2 nM (**9**) to 10,100 nM (**12**). It is anticipated that either

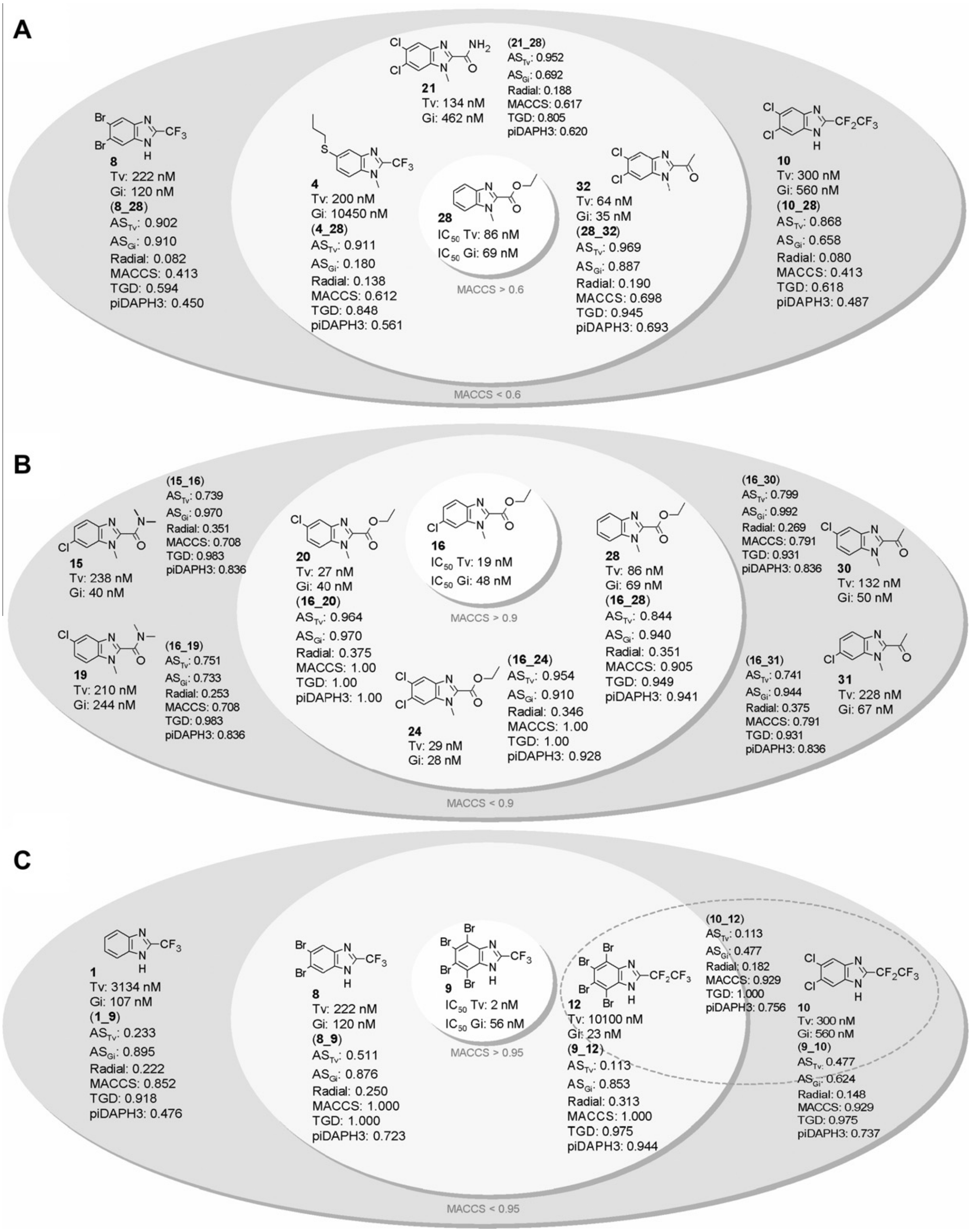

Figure 6. Representative consensus pairs in the activity landscapes of *T. vaginalis* and *G. intestinalis* arranged in concentric similarity ovals. Compounds in the inner ring are more structurally similar to the reference (center) than compounds in the outer ring: (A) region I, side chain hopping; (B) region II, smooth SAR, and (C) activity cliffs.

of these two compounds or both would be apparent outliers in a QSAR study.[15] Interestingly, **9_12** was not an activity cliff for *G. intestinalis* (IC_{50} = 56 nM vs 23 nM, respectively). A second consensus deep cliff in the landscape of *T. vaginalis* was the pair **1_9**, detected by radial and MACCS keys only. This is an example of an *apparent cliff* conceptualized as cliff identified just for some molecular representations.[26] One more example of an apparent cliff is the pair **9_10** identified by MACCS keys and TGD. A borderline case between deep and shallow cliff for *T. vaginalis* is **8_9** (activity similarity value of 0.511) identified as cliff by radial, MACCS and TGD (but not by piDAPH3). Note, however, that MACCS and TGD could not distinguish this pair (similarity = 1).

Few consensus activity cliffs were identified in the landscape of *G. intestinalis*. For example, the pair **10_12** (Table 4 and Figs. 4 and

6C) was identified as a deep cliff by MACCS and TGD only (e.g., apparent cliff). As discussed above, when the activity similarity threshold is set to less restrictive changes in potency difference, the number of activity cliffs increases. An example of a shallow (and also apparent) cliff was the pair **9_10** identified by MACCS and TGD. This pair was also identified as an apparent cliff in the landscape of *T. vaginalis* (Table 4).

The presence of pairs of compounds in continuous and discontinuous regions of the landscape for *T. vaginalis* and *G. intestinalis* revealed the heterogeneous SAR for both parasites. Heterogeneous SARs has been reported for other activity classes.[14,26,27] The landscape of *T. vaginalis* is characterized by the presence of two consensus deep activity cliffs and several data points in continuous regions of the SAR. In contrast, the landscape of *G. intestinalis* did not show consensus deep activity cliffs but a larger number of shallow cliffs as compared to *T. vaginalis* (Table S1).

3.3.6. Consensus models of the activity landscape: Consensus SAS maps

The activity landscape depends on the molecular representation.[26,27] However, the consensus pairs found in several regions of the landscape suggests the possibility to derive, at least approximately, consensus models of the activity landscape. To this end, we employed in this work the principles of data fusion[38] producing Consensus SAS maps. For each pair of compounds, we calculated the mean and standard deviation of radial, MACCS, TGD and piDAPH3 similarity values (four selected orthogonal fingerprints) as detailed in Section 2. Figures 3E and 4E show the consensus SAS maps for *T. vaginalis* and *G. intestinalis*, respectively. In these figures, data points are colored by the most active compound in the pair using the same color scheme as in the SAS maps. Consensus SAS maps showing data points sized by standard deviation are in Figure S5 of the Supporting information. Figures 3E and 4E show the position of representative consensus pairs previously identified by radial, MACCS, TGD and piDAPH3 fingerprints (Figs. 3A–D and 4A–D). The mean fingerprint similarity values and standard deviation is provided in Table 4. In general, the pair of compounds in the consensus SAS map in Figure 3E occupies a relatively similar position (regions I–IV) in the SAS maps obtained separately with the radial, MACCS, TGD and piDAPH3 fingerprints (Fig. 3A–D). Similar results were obtained comparing the consensus SAS map of *G. intestinalis* (Fig. 4E) with the SAS maps obtained independently with the four fingerprints (Fig. 4A–D). Therefore, the consensus SAS maps effectively capture the information obtained with the different independent fingerprint representations and provide a good approximation of the overall activity landscape of the benzimidazoles tested against *T. vaginalis* and *G. intestinalis*. These results suggested that consensus SAS maps could provide valuable information for other data sets with other biological endpoints.[42]

3.5. Dual-parasite SAR and consensus selectivity cliffs

We compared the activity of the 32 benzimidazoles against the two parasites. The difference of pIC_{50} values is indicated in Table 1. Several compounds showed a similar activity (low ΔpIC_{50}) with *T. vaginalis* and *G. intestinalis*. For example, compounds **13**, **14**, **17**–**19**, **20**, **22**–**24**, **27** and **28** have a $|\Delta pIC_{50}| < 0.20$. These results suggest that these non 2-methylcarbamates have a common mechanism of action against the two protozoan.[43] In addition, these results encourage the simultaneous lead optimization of compounds active against both *T. vaginalis* and *G. intestinalis*.

We also identified molecules with large potency difference against the two parasites. Compounds **1**, **6** and **12** are selective for *G. intestinalis*, whereas **4** and **9** are selective for *T. vaginalis* with more than one log unit in potency difference, respectively (Table 1). Interestingly, the pair of compounds **9_12** has a high structural similarity; the only difference is a CF_2 group (this difference was captured by all similarity methods, Table 4). However, the selectivity is quite different. This is an example of a selectivity cliff where a small change in the structure has a major impact in the selectivity.[25] A second example of a selectivity cliff was the pair **1_9** indicating that introducing bromine atoms in 2-(trifluoromethyl)benzimidazole has an opposite effect in the selectivity profile (Table 1). The pair **8_9** also has high structural similarity (Table 4; the difference is two bromine atoms at positions 4 and 7) but produces an opposite change in the activity against the two parasites. However, the effect for the pair **8_9** is less dramatic than for the pairs **9_12** and **1_9**. To note, **1_9** and **9_12** are deep cliffs while **8_9** is a shallow cliff in the landscape of *T. vaginalis*. However, the same pairs are in a smooth region of the landscape of *G. intestinalis*.

4. Conclusions and perspectives

We report a systematic characterization of the SAR of 32 (non 2-methylcarbamate) benzimidazoles with activity against *T. vaginalis* and *G. intestinalis*. The analysis was based on pairwise comparisons of the activity similarity and molecular similarity using different molecular representations. We found that radial, MACCS keys, TGD and piDAPH3 fingerprints showed low correlation (<0.80) and similar (approximately normal) distributions. These fingerprints, along with molecular properties, were used to characterize the activity landscape for each parasite. To note, the purpose of using multiple molecular representations was not to identify the 'best' representation but to identify the set of fingerprint representations that best identifies consensus data pairs. The landscape was portrayed using structure–activity similarity (SAS) maps which were quantitatively compared using the degree of consensus. The overall good consensus between structural representations allowed for the development of consensus models and consensus SAS maps to represent the activity landscape of *T. vaginalis* and *G. intestinalis*. For both parasites, several consensus pairs of compounds were identified in the smooth region of the landscape. Also a number of pairs were identified in the side chain hopping region. For *T. vaginalis*, we identified two deep consensus activity cliffs (**1_9** and **9_12**). It is anticipated that these compounds will be apparent 'outliers' in traditional computational models such as QSAR. In contrast, for *G. intestinalis*, we identified apparent cliffs and shallow cliffs. In conclusion, a heterogeneous SAR was found for both parasites. Comparison of the compounds' selectivity for each parasite revealed that several compounds are active against *T. vaginalis* and *G. intestinalis* showing similar SAR. We concluded that these molecules may have similar mechanism of action in both parasites and encourage simultaneous lead optimization efforts against both organisms. However, we also detected molecules with opposite selectivity profile and consensus selectivity cliffs.

Despite the fact that the data set of molecules studied in this work share the same core scaffold, whole-molecule fingerprint-based similarity methods were able to study the activity landscape, derive a consensus model of the landscape and, in particular, detect activity cliffs. Radial fingerprints were able to distinguish the molecules in great detail and differentiate positional isomers.

We want to emphasize that the present study is a systematic description of the activity landscape of a data set of 32 benzimidazole derivatives. This systematic study of the SAR will be expanded to larger data sets including compounds currently synthesized and tested. A second major perspective of this work is to explore the predictive capabilities of the activity landscape models to anticipate the SAR of new molecules in a prospective manner. This is an area of intense research in our and other

research groups. The systematic approach presented here to develop consensus models of the activity landscape of benzimidazole analogues against *T. vaginalis* and *G. intestinalis* is general. The approach can be applied to other larger data sets with other biological end points.

Acknowledgements

The authors would like to express their sincere thanks to Dr. Gerald M. Maggiora, for insightful discussions. We are also very grateful to Karen Gottwald for proofreading the manuscript. This work was supported by the State of Florida, Executive Office of the Governor's Office of Tourism, Trade, and Economic Development. J.P-V is supported by CONACyT and Carso Health Institute PhD studentships. J.P-V also thanks the State of Florida for partial support of his research visit at TPIMS.

Supplementary data

Total number of shallow cliffs according to different molecular representations (Table S1); active regions in the SAS maps for *T. vaginalis* (Fig. S1); active regions in the SAS maps for *G. intestinalis* (Fig. S2); examples of binned SAS maps (Fig. S3); number of consensus pairs between different molecular representations (Fig. S4); consensus SAS maps showing data points sized by standard deviation (Fig. S5). Supplementary data associated with this article can be found, in the online version, at doi:10.1016/j.bmc.2010.09.019.

References and notes

1. World Health Organization. Global Prevalence and Incidence of Selected Curable Sexually Transmitted Infections: Overview and Estimates, Geneva, 2001.
2. Upcroft, P.; Upcroft, J. A. *Clin. Microbiol. Rev.* **2001**, *14*, 150.
3. Berkman, D. S.; Lescano, A. G.; Gilman, R. H.; Lopez, S.; Black, M. M. *Lancet* **2002**, *359*, 564.
4. Navarrete-Vazquez, G.; Rojano-Vilchis, M. D.; Yepez-Mulia, L.; Melendez, V.; Gerena, L.; Hernandez-Campos, A.; Castillo, R.; Hernandez-Luis, F. *Eur. J. Med. Chem.* **2006**, *41*, 135.
5. Navarrete-Vázquez, G.; Yépez, L.; Hernández-Campos, A.; Tapia, A.; Hernández-Luis, F.; Cedillo, R.; González, J.; Martínez-Fernández, A.; Martínez-Grueiro, M.; Castillo, R. *Bioorg. Med. Chem.* **2003**, *11*, 4615.
6. Andrzejewska, M.; Yépez-Mulia, L.; Cedillo-Rivera, R.; Tapia, A.; Vilpo, L.; Vilpo, J.; Kazimierczuk, Z. *Eur. J. Med. Chem.* **2002**, *37*, 973.
7. Valdez-Padilla, D.; Rodríguez-Morales, S.; Hernández-Campos, A.; Hernández-Luis, F.; Yépez-Mulia, L.; Tapia-Contreras, A.; Castillo, R. *Bioorg. Med. Chem.* **2009**, *17*, 1724.
8. Ooms, F. *Curr. Med. Chem.* **2000**, *7*, 141.
9. Medina-Franco, J. L.; Lopez-Vallejo, F.; Castillo, R. *Educ. Quím.* **2006**, *17*, 452.
10. Kubinyi, H. *Drug Discovery Today* **1997**, *2*, 457.
11. Kubinyi, H. *Drug Discovery Today* **1997**, *2*, 538.
12. Scior, T.; Medina-Franco, J. L.; Do, Q. T.; Martinez-Mayorga, K.; Yunes Rojas, J. A.; Bernard, P. *Curr. Med. Chem.* **2009**, *16*, 4297.
13. Klebe, G. *J. Mol. Med.* **2000**, *78*, 269.
14. Peltason, L.; Bajorath, J. *Chem. Biol.* **2007**, *14*, 489.
15. Maggiora, G. M. *J. Chem. Inf. Model.* **2006**, *46*, 1535.
16. Guha, R.; VanDrie, J. H. *J. Chem. Inf. Model.* **2008**, *48*, 646.
17. Bajorath, J.; Peltason, L.; Wawer, M.; Guha, R.; Lajiness, M. S.; Van Drie, J. H. *Drug Discovery Today* **2009**, *14*, 698.
18. Schneider, G.; Neidhart, W.; Giller, T.; Schmid, G. *Angew. Chem., Int. Ed.* **1999**, *38*, 2894.
19. Eckert, H.; Bajorath, J. *Drug Discovery Today* **2007**, *12*, 225.
20. Medina-Franco, J. L.; Martinez-Mayorga, K.; Giulianotti, M. A.; Houghten, R. A.; Pinilla, C. *Curr. Comput.-Aided Drug Des.* **2008**, *4*, 322.
21. Peltason, L.; Bajorath, J. *J. Med. Chem.* **2007**, *50*, 5571.
22. Guha, R.; Van Drie, J. H. *J. Chem. Inf. Model.* **2008**, *48*, 1716.
23. Shanmugasundaram, V.; Maggiora, G. M. 222nd ACS National Meeting, Chicago, IL, United States; American Chemical Society, Washington, D. C: Chicago, IL, United States, 2001.
24. Wawer, M.; Peltason, L.; Weskamp, N.; Teckentrup, A.; Bajorath, J. *J. Med. Chem.* **2008**, *51*, 6075.
25. Peltason, L.; Hu, Y.; Bajorath, J. *ChemMedChem* **2009**, *4*, 1864.
26. Medina-Franco, J. L.; Martínez-Mayorga, K.; Bender, A.; Marín, R. M.; Giulianotti, M. A.; Pinilla, C.; Houghten, R. A. *J. Chem. Inf. Model.* **2009**, *49*, 477.
27. Peltason, L.; Iyer, P.; Bajorath, J. *J. Chem. Inf. Model.* **2010**, *50*, 1021.
28. Navarrete-Vázquez, G.; Cedillo, R.; Hernández-Campos, A.; Yépez, L.; Hernández-Luis, F.; Valdez, J.; Morales, R.; Cortés, R.; Hernández, M.; Castillo, R. *Bioorg. Med. Chem. Lett.* **2001**, *11*, 187.
29. Valdez, J.; Cedillo, R.; Hernandez-Campos, A.; Yepez, L.; Hernandez-Luis, F.; Navarrete-Vazquez, G.; Tapia, A.; Cortes, R.; Hernandez, M.; Castillo, R. *Bioorg. Med. Chem. Lett.* **2002**, *12*, 2221.
30. Jaccard, P. *Bull. Soc. Vaudoise Sci. Nat.* **1901**, *37*, 547.
31. Willett, P.; Barnard, J. M.; Downs, G. M. *J. Chem. Inf. Comput. Sci.* **1998**, *38*, 983.
32. Molecular Operating Environment (MOE), version 2009.10, Chemical Computing Group, Montreal, Quebec, Canada. http://www.chemcomp.com (accesssed August, 2010).
33. Canvas, version 1.3, Schrödinger, LLC, New York, NY, 2010.
34. Sastry, M.; Lowrie, J. F.; Dixon, S. L.; Sherman, W. *J. Chem. Inf. Model.* **2010**, *50*, 771.
35. Conformational analysis for molecules with rotatable bonds was conducted by means of systematic search with torsions of 60° using MMFF94 force field. Equilibrium geometry calculations were carried out for the minimum energy conformer using PM3 semiempirical method.
36. Spartan, version 2002, Wavefunction, Irvine, CA. http://www.wavefun.com (accessed August, 2010).
37. A similar term, 'R-hopping' was proposed by Prof. Jürgen Bajorath (personal communication).
38. Willett, P. *Drug Discovery Today* **2006**, *11*, 1046.
39. Rogers, D.; Hahn, M. *J. Chem. Inf. Model.* **2010**, *50*, 742.
40. The only exception were regions II and III of the SAS map for *T. vaginalis* obtained with TGD; both regions have similar population.
41. Johnson, M. A.; Maggiora, G. M. *Concepts and Applications of Molecular Similarity*; Wiley: New York, 1990.
42. By analogy with the SAS maps, consensus SAS maps could be analyzed quantitatively dividing the maps into four quadrants by imposing thresholds for activity and molecular similarity.
43. Related methyl 1*H*-benzimidazole-2-yl-carbamate derivatives are strong selective inhibitors of tubulin polymerization. However, it is required that the benzimidazole scaffold has a 2-methylcarbamate group and hydrogen at position 1. The mechanism of non 2-methylcarbamates remains to be determined.
